# Genome of *Paspalum vaginatum* and the role of trehalose mediated autophagy in increasing maize biomass

**DOI:** 10.1038/s41467-022-35507-8

**Published:** 2022-12-13

**Authors:** Guangchao Sun, Nishikant Wase, Shengqiang Shu, Jerry Jenkins, Bangjun Zhou, J. Vladimir Torres-Rodríguez, Cindy Chen, Laura Sandor, Chris Plott, Yuko Yoshinga, Christopher Daum, Peng Qi, Kerrie Barry, Anna Lipzen, Luke Berry, Connor Pedersen, Thomas Gottilla, Ashley Foltz, Huihui Yu, Ronan O’Malley, Chi Zhang, Katrien M. Devos, Brandi Sigmon, Bin Yu, Toshihiro Obata, Jeremy Schmutz, James C. Schnable

**Affiliations:** 1 Quantitative Life Sciences Initiative, University of Nebraska-Lincoln, Lincoln, NE 68588 USA; 2 Center for Plant Science Innovation, University of Nebraska-Lincoln, Lincoln, NE 68588 USA; 3 Department of Agronomy and Horticulture, University of Nebraska-Lincoln, Lincoln, NE 68588 USA; 4 Department of Biochemistry, University of Nebraska-Lincoln, Lincoln, NE 68588 USA; 5 Biomolecular Analysis Facility. School of Medicine, University of Virginia, Charlottesville, VA 22903 USA; 6 Department of Energy Joint Genome Institute, Lawrence Berkeley National Laboratory, Lawrence, CA 94720 USA; 7 HudsonAlpha Institute for Biotechnology, Huntsville, AL 35806 USA; 8 School of Biological Sciences, University of Nebraska-Lincoln, Lincoln, NE 68588 USA; 9 Institute of Plant Breeding, Genetics and Genomics, Department of Crop and Soil Sciences, University of Georgia, Athens, GA 30602 USA; 10 Department of Crop and Soil Sciences, University of Georgia, Athens, GA 30602 USA; 11 Department of Plant Biology, University of Georgia, Athens, GA 30602 USA; 12 Department of Plant Pathology, University of Nebraska-Lincoln, Lincoln, NE 68588 USA

**Keywords:** Genomics, Plant physiology, Abiotic, Agricultural genetics

## Abstract

A number of crop wild relatives can tolerate extreme stress to a degree outside the range observed in their domesticated relatives. However, it is unclear whether or how the molecular mechanisms employed by these species can be translated to domesticated crops. Paspalum (*Paspalum vaginatum*) is a self-incompatible and multiply stress-tolerant wild relative of maize and sorghum. Here, we describe the sequencing and pseudomolecule level assembly of a vegetatively propagated accession of *P. vaginatum*. Phylogenetic analysis based on 6,151 single-copy syntenic orthologues conserved in 6 related grass species places paspalum as an outgroup of the maize-sorghum clade. In parallel metabolic experiments, paspalum, but neither maize nor sorghum, exhibits a significant increase in trehalose when grown under nutrient-deficit conditions. Inducing trehalose accumulation in maize, imitating the metabolic phenotype of paspalum, results in autophagy dependent increases in biomass accumulation.

## Abstract (web summary)

*Paspalum vaginatum* is a stress tolerant wild relative of maize and sorghum. Here, the authors assemble its genome at pseudomolecule level and reveal the role of trehalose mediated autophagy in increasing maize biomass productivity under nutrient-deficit conditions.

## Introduction

Domesticated crops from the grass family provide, directly or indirectly, the majority of the total calories consumed by humans around the globe. Among domesticated grasses, the yields of three crops dramatically increased as part of the green revolution: rice (*Oryza sativa*), wheat (*Triticum aestivum*) and maize (*Zea mays*). These yield increases resulted from both breeding and greater availability and application of fertilizer. From 1960 to 2014, the amount of nitrogen (N) and phosphorus (P) fertilizer applied worldwide increased nine- and five-fold, respectively^1–3^. Today these three crops account for approximately one half of the total harvested staple crop area and total global calorie production as well as greater than one half of total global fertilizer consumption. Manufacturing N fertilizer is an energy-intensive process^4^ and the production of P from mineral sources may peak as early as 2030^5^. Fertilizer costs are often the second largest variable input after seed in rain-fed agricultural systems. In the United States Corn Belt alone, 5.6 million tons of N and 2.0 million tons of P have been applied annually to maize fields since 2010^6^. In the 2015 growing season, these fertilizers accounted for an estimated $5 billion in input costs^7,8^. Fertilizer runoff resulting from inefficient uptake or over application can result in damage to both aquatic ecosystems and drinking water quality^9–12^.

Improving the productivity of crop plants per unit of fertilizer applied would increase the profitability of agriculture while decreasing its environmental impact^13–15^. A significant portion of the overall increase in maize yields appears to be explained by selection for increased stress tolerance and yield stability since the 1930s^16,17^. The observations from maize suggest it may be possible to increase the stress tolerance and resource-use efficiency of crops in a manner that is either neutral or beneficial to overall yield potential. Some crop wild relatives exhibit degrees of stress tolerance well outside the range observed in their domesticated relatives, and therefore may employ mechanisms not present in the primary germplasm of crops^14^.

*Paspalum vaginatum* (seashore paspalum—or simply paspalum) is a relative of maize and sorghum (*Sorghum bicolor*). It is currently found on saltwater beaches and in other regions of high salinity around the globe^18,19^. Reports suggest that paspalum is tolerant of drought^20–23^, cold stress^24–26^, low light^27^, and crude oil contamination^28^. Paspalum grows primarily in the wild, but breeding efforts have led to the development of turfgrass cultivars for use in areas with high soil salinity, limited access to freshwater, or where turf is irrigated with wastewater^27,29^. Paspalum requires less N to maintain visible health than other grasses employed as turfgrasses in environments where it thrives^29^. Historically few genetic resources have been available for this species, although a set of genetic maps were recently published^30^. The paucity of genetic and genomic investigations may in part result from the challenging reproductive biology of this species; paspalum is self-incompatible and is primarily propagated as heterozygous vegetative clones^29,31^.

Here, we generate a pseudomolecule level genome assembly for a reference genotype of paspalum (PI 509022), enabling comparative transcriptomic and genomics analysis. The paspalum genome exhibits a high degree of conserved collinearity with that of sorghum. Changes in trehalose accumulation in response to multiple nutrient-deficit stresses are observable in paspalum, but not in paired datasets collected from sorghum and maize under identical conditions. Inhibiting the enzyme responsible for degrading trehalose in maize increases trehalose accumulation, mimicking the biochemical phenotype observed in paspalum under nutrient deficit stress, and results in increased biomass accumulation. Increased biomass accumulation associated with induced accumulation of trehalose is abolished in maize plants homozygous for mutations of *atg12*, which encodes a core component of the autophagy system. Collectively these results suggest autophagy as a potential mechanism for the increased productivity observed in maize plants accumulating additional trehalose.

## Results

### Characteristics of the paspalum genome

**Fig. 1:**
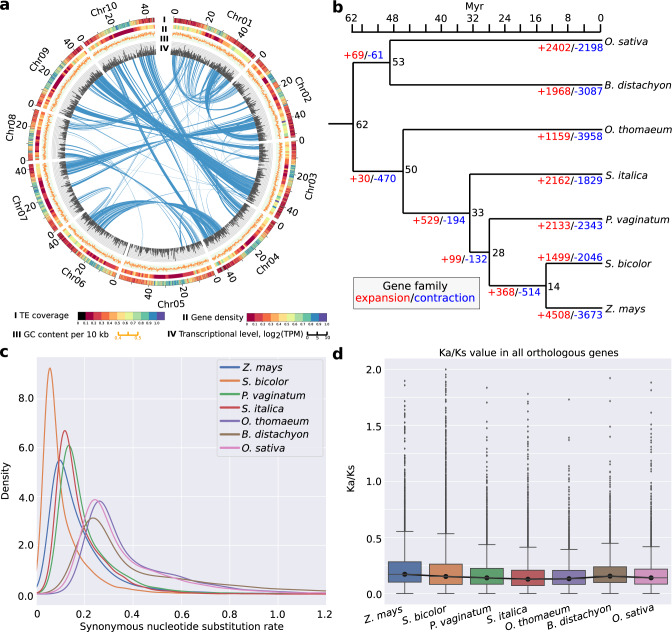
Paspalum (*Paspalum vaginatum*) genome and evolution. **a** Properties of the paspalum genome. Layers in circos plots are (I): TE (transposable element) coverage per 100 Kb, (II): gene density (proportion of sequence covered by annotated genes) per 1 Mb (megabase), (III): GC content per 10 kb (kilobase) region, (IV): transcription represented by log_2_(TPM (transcripts per million)) per 100 kb. Green lines indicate inter- and intra- chromosomal synteny. **b** Phylogeny and estimated divergence times among maize (*Zea mays*), sorghum, paspalum, foxtail millet (*Setaria italica*), *Oropetium* (*Oropetium thomaeum*), *Brachypodium* (*Brachypodium distachyon*), and rice (*Oryza sativa*). Numbers in black indicate the estimated divergence time in millions of years before present (Myr) for each node. Numbers in blue and red indicate the number of gene families predicted to have experienced copy number expansion or contraction along each branch of the phylogeny, respectively. **c** Distribution of the estimated lineage-specific synonymous substitution rates for syntenically conserved genes in each of the seven species shown in panel **a** (see Methods). **d** Distribution of the estimated lineage-specific ratios of nonsynonymous substitution rates to synonymous substitution rates for syntenically conserved genes among each of the seven species shown in panel **a**. *N* = 16,633 distinct genes. For box plots in this figure and all subsequent box plots shown in this paper center lines indicate medians, box limits indicate 25th and 75th percentiles if the data distribution and whiskers the closer to the median of the minimum and maximum values or 1.5x the interquartile range, with data points outside this range plotted as individual points. Colors correspond to the legend provided in panel **c**. Source data are provided as a Source Data file.

We generated 5,021,142 PacBio reads with a median length of 9523 bp from genomic DNA isolated from dark-treated tissue of the heterozygous paspalum clone PI 509022. The reads were assembled into 1902 main genome scaffolds with an N50 of 44.5 Mbp and a total length of 646.9 Mbp (Supplementary Table 1). This is modestly larger than the estimated haploid gene size of the Paspalum genome of 593 Mbp (See Methods)^32^. Flow cytometry carried out within this study confirmed that the genome size of the paspalum clone employed in this study was ~590 Mbp (Supplementary Fig. 1a–c). This modest over-assembly may represent haplotype-specific sequences which is supported by the bimodel distribution of read coverage mapped to the current genome assembly (Supplementary Fig. 1d). We used published sequence data from markers genetically mapped in an F1 population generated from a cross between two heterozygous paspalum individuals^30^ to integrate a set of 347 scaffolds into ten pseudomolecules spanning >82% of the estimated total haploid paspalum genome (Supplementary Data 1). Scaffolds that were not anchored in a chromosome were classified into bins depending on sequence content. Contamination was identified using BLASTN against the NCBI nucleotide collection (NR/NT) and BLASTX using a set of known microbial proteins. Additional scaffolds were classified as repetitive (>95% masked with 24 mers that occur more than 4 times in the genome) (197 scaffolds, 12.4 Mb), alternative haplotypes (unanchored sequence with >95% identity and >95% coverage within a chromosome) (3276 scaffolds, 187.9 Mb), and low quality (>50% unpolished bases, post polishing) (9 scaffolds, 204.5 Kb) (Supplementary Table 1). A set of 45,843 gene models were identified and annotated using a combination of approaches (see Methods). A total of 22,148 syntenic orthologous gene pairs were identified between the paspalum and sorghum genomes (Supplementary Fig. 2a, b). The large inversions observed on chromosome 4 and chromosome 7 were previously validated by a study where a genetic map was constructed using GBS genotyping technology^30^. Small translocations were also observed between chromosome 8 and chromosome 4 (Supplementary Fig. 2a, b). The predicted protein sequences of annotated paspalum genes tended to cover the full length of the most closely related protein in sorghum, and vice versa, indicating most annotated gene models in the paspalum genome assembly are likely full length (Supplementary Fig. 2c). On a macro level, the paspalum genome displays many features common to other grass genomes: a higher gene density in the distal chromosome regions than pericentromeric regions and, conversely, a higher frequency of transposable elements and other repetitive sequences in pericentromeric regions than distal chromosome regions, and syntenic evidence of the pre-grass (rho) whole-genome duplication (Fig. 1a).

### Comparative genomics analysis of paspalum and its relatives

Paspalum belongs to the grass tribe Paspaleae, a group which, together with the Andropogoneae (which includes maize and sorghum)—and the Arundinelleae, forms a clade sister to the Paniceae—which includes foxtail millet (*Setaria italica*). Paspaleae, Andropogoneae and Paniceae are all members of the grass subfamily Panicoideae, while *Oropetium* belongs to the grass clade Cynodonteae^33^. We constructed phylogenic trees using DNA alignments for 6151 single-copy syntenic orthologous genes present in *Zea mays*, *Sorghum bicolor*, *Setaria italica*, *Oropetium thomaeum*, *Brachypodium distachyon*, *Oryza sativa*, and *Paspalum vaginatum*. A total of 5,859 trees placing *B. distachyon* and *O. sativa* in a monophyletic outgroup survived quality filtering (see Methods for filtering criteria). Forty nine unique topologies were supported by these trees with the most common topology shown in DensiTree(v2.2.6)—represented by 4,265 individual gene trees (73%)—being consistent with the previous consensus placement of paspalum (Supplementary Fig. 3a and Fig. 1b). The second most common topology, represented by 762 individual gene trees (13%), placed paspalum sister to foxtail millet (Supplementary Fig. 3a).

Dating placed the split of the Chloridoideae (represented by *Oropetium thomaeum*) from the Panicoideae at 50 million years before the present and indicated that, within the Panicoideae, the Paniceae shared a common ancestor with the Andropogoneae/Paspaleae clade (represented by paspalum, sorghum, and maize) at 33 million years (Myr) before present, a date modestly earlier than previous estimates (~26 Myr ago)^34,35^ (Fig. 1b). The divergence of the lineage leading to paspalum from that leading to maize and sorghum—(the split between the Andropogoneae and Paspaleae)—was estimated to have occurred ~28 million years before present. We calculated branch-specific synonymous (Ks) and nonsynonymous (Ka) nucleotide substitution rates for syntenic orthologous gene groups based on known species relationships (Fig. 1c; Supplementary Data 2). Consistent with previous reports, maize exhibited greater modal synonymous substitution rates than sorghum, even though these are sister lineages in the phylogeny^36^ (Fig. 1c). The modal synonymous substitution rates in paspalum were modestly higher than those observed in foxtail millet (Fig. 1d).

Annotated protein sequences for sorghum, foxtail millet, *Oropetium*, *Brachypodium* (*Brachypodium distachyon*), and paspalum were grouped into 25,675 gene families. Of these families, 16,038 were represented by at least one gene copy in each of the five species, with the remainder being present in 1–4 species (Supplementary Fig. 3b). A set of 721 gene families were unique to paspalum. This number was modestly less than the number of species-specific gene families identified in brachypodium and modestly more than the number of species-specific gene families observed in sorghum and foxtail millet (Supplementary Fig. 3b). Of the 21,091 gene families present in paspalum, 75% (15,769) were represented by only a single copy in the paspalum genome and 17 % (3524) were represented by two copies. These values are similar to those observed in sorghum and foxtail millet which shared the same most recent common pre-grass (rho) whole-genome duplication (Supplementary Fig. 3c). A set of 149 gene families were identified as undergoing copy number expansion with a significantly different evolution rate (lambda) in the paspalum lineage. These included families of genes annotated with gene ontology (GO) terms related to homeostatic processes such as telomere maintenance and DNA repair, protein modification, stress response, nutrient reservoir activity, and oxidation-reduction process (Supplementary Data 3; Fisher’s exact test with Benjamini-Hochberg false discovery control^37^).

### General and paspalum-specific physiological responses to nutrient-deficit stress

**Fig. 2:**
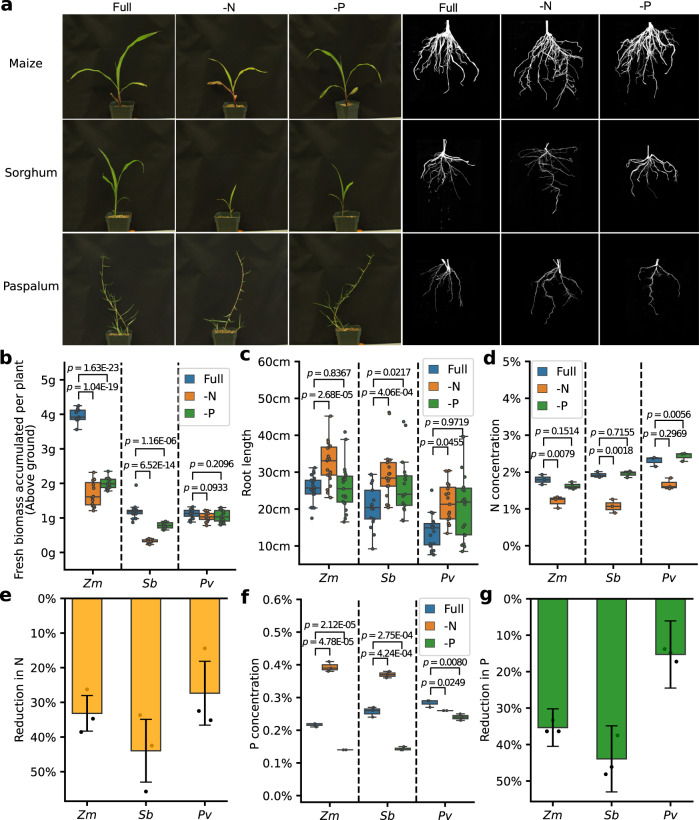
Responses of three studied species to nutrient-deficit stress. **a** Representative images of above and below ground organs of maize, sorghum, and paspalum ramets at 21 days after panting (dap) under optimal (Full), nitrogen-deficit (-N), or phosphorus-deficit conditions (-P). **b** Fresh biomass accumulated in the first 21 days after planting for maize and sorghum seedings and paspalum ramets under control, -N or -P conditions (Data shown are from three independent replicates each with ≥5 samples; *N* = 15 individually measured plants). **c** Changes in root length relative to Full at 21 dap under -N or -P conditions in maize, sorghum, and paspalum (*N* = 15 individually measured plants, repeated in triplicates). **d**, **e** Abundance (**d**) and reduction (**e**) of N as a proportion of total dry biomass in the shoots of maize, sorghum seedlings, and paspalum ramets at 21 dap. Error bars in panel **e** indicate standard deviation across biological replicates. *N* = 3 biological replicates, each consisting of a measurement of N abundance from the pooled tissue of five plants per condition. **f**, **g** Abundance (**f**) and reduction (**g**) of P as a proportion of total dry biomass in the shoots of maize, sorghum seedlings, and paspalum ramets at 21 dap. Error bars in panel **g** indicate standard deviation across biological replicates. *N* = 3 biological replicates, each consisting of measurements of P abundance from the pooled tissue of five or more plants per condition. For **b**, **c**, **d** and **f**, *p* values are determined by two-sided Wilcoxon signed-rank test. (*Zm* = maize, *Sb* = sorghum and *Pv* = paspalum). Source data are provided as a Source Data file.

Paspalum requires little fertilizer in order to remain visibly healthy^29^, however, it was unclear whether paspalum is actually more efficient at producing biomass under nitrogen-limited conditions. A comparison was conducted of the growth of paspalum, sorghum, and maize plants under nutrient-sufficient, nitrogen-limited, and phosphorus-limited conditions. Visible effects were apparent in maize and sorghum seedlings under N- or P-deficient conditions but not in clonally-propagated paspalum plants (ramets) three weeks after planting (Fig. 2a). Both maize and sorghum exhibited significant decreases in above ground fresh biomass accumulation when grown under N- or P- deficient conditions whereas paspalum did not (Fig. 2b). This result should be interpreted with the caveat that, despite of a similar level of fresh biomass accumulation as sorghum, paspalum accumulated the least biomass per plant of the three species under nutrient-optimal conditions. In both maize and sorghum, N-deficit was associated with significant increases in root length (Fig. 2a, c). However, a statistically significant increase in root length in response to P-deficit stress was only observed for sorghum (Fig. 2c). Paspalum ramets grown under N-deficient conditions showed a modest but statistically significant increase in root length compared to optimal nutrient conditions, while P-deficit stress did not produce any statistically significant increase in root length in this species (Fig. 2c). In time series data collected at 9, 15, 21, and 28 days after planting, sorghum accumulated substantially less biomass under nitrogen-deficient conditions relative to full-nutrient conditions (Supplementary Fig. 4a, b) while no significant difference in biomass accumulation was observed between treatments for paspalum at any time point and paspalum accumulated substantially more biomass than did sorghum (Supplementary Fig. 4c, d).

One potential explanation for the relative lack of plasticity observed in paspalum in response to N-deficit or P-deficit stress is that paspalum has limited potential to utilize N under nutrient-sufficient conditions and hence did not experience substantial internal declines in N availability in response to N-deficient conditions. We therefore measured the contents of N and P in plants of all three species included in this study. Substantial decreases in N as a proportion of total biomass were observed in all three species grown under N-deficient conditions relative to the full-nutrient controls (Fig. 2d, e). P-deficient treatments produced significant declines in P as a proportion of dry biomass for all three species (Fig. 2f), although the decline in P abundance for paspalum was notably smaller in magnitude than the declines in maize and sorghum, with sorghum exhibiting the greatest reduction in P content (43%), followed by maize (36%) and paspalum (15%; Fig. 2g). N-deficient treatment produced significant increases in P as a proportion of dry biomass in maize and sorghum (Fig. 2f), which is consistent with previous reports of enhanced P uptake in plants grown under N-deficient conditions^38^. Taken as a whole, these results indicate that the external N-deficient treatment protocol employed here was sufficient to produce declines in internal N levels and N-deficit stress in paspalum.

### Comparisons of primary metabolic responses to nutrient-deficit stress

**Fig. 3:**
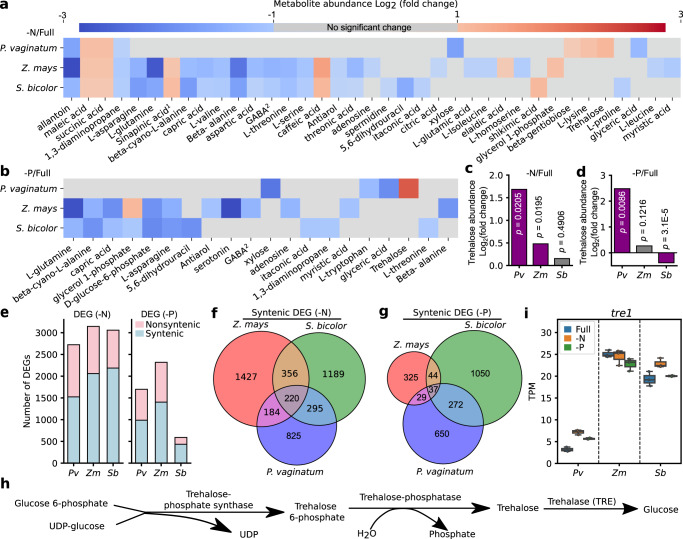
Primary metabolic and transcriptomic responses of three studied to nutrient-deficit stress. **a**, **b** Changes in the abundance of metabolites in the roots of paspalum (*Pv*), maize (*Zm*) and sorghum (*Sb*) under -N conditions (**a**) and -P condition (**b**) at 21 dap relative to plants grown under Full condition. Only the metabolites with a statistically significance change in abundance (*p* < 0.05; *t*-test; two *t*ailed) and an absolute fold change >2 in at least one of the three species evaluated are shown. Raw data and exact p-values are included in Supplementary Data 4. **c**, **d** Change in trehalose abundance in the roots of 3-week-old plants under -N (**c**) and -P (**d**) conditions relative to control plants. Significant changes are defined as *p* values (*t*-test; two tailed) lower than 0.05 and log_2_ fold change higher than 1 and are indicated in purple, and non-significant changes are indicated in gray. For **a**–**d**: *N* = 5 independent biological replicates consisting of pooled tissue from multiple individual plants. **e** Number of significantly differentially expressed genes (DEGs) in the roots of three week old plants relative to controls. Identified based on three biological replicates per species per condition, each consisting of RNA extracted from a pool of multiple plants. **f**, **g** Number of syntenically conserved orthologous triplets exhibiting shared or species-specific differential expression in response to -N (**f**) and -P conditions (**g**). **h** Simplified diagram of the trehalose metabolic pathway. **i** Expression levels of the gene encoding trehalase *tre1* in the roots of 3-week-old plants, expressed in transcripts per million (TPM). *N* = 3 biological replicates per species per condition, each consisting of RNA extracted from a pool of multiple plants. Box plots shown in panel **i** is defined as in the Fig. 1 legend. Source data are provided as a Source Data file.

Numerous metabolic changes were observed between plants grown under nutrient-sufficient and nutrient-deficient conditions, with more metabolites exhibiting significant changes in abundance in response to N- or P-deficit stress in maize and sorghum than in paspalum (Fig. 3a, b, Supplementary Data 4; paired two-tailed *t*-test). Fourteen metabolites showed significant decreases in abundance in response to N-deficit stress in both sorghum and maize, and four metabolites showed significantly increased abundance in response to N-deficit stress (18 of the 32 metabolic responses were shared between the two species) (Fig. 3a). A smaller number of statistically significant metabolic changes were observed in response to P-deficit stress, which is consistent with the less severe phenotype observed for P deficiency in the experimental design employed (Figs. 2a, 3b). A number of metabolic changes were again shared between maize and sorghum, with the levels of five tested metabolites decreasing in both species in response to P-deficit stress and one increasing (6 of the 16 metabolic responses were shared) (Fig. 3b). All metabolic changes associated with N-deficit stress in paspalum were either species specific or shared with both maize and sorghum while all metabolic changes associated with P-deficit stress in paspalum were species specific (Fig. 3a, b). Metabolic changes associated with N-deficit stress shared by maize and sorghum but not paspalum included decreases in the abundance of many amino acids, such as L-asparagin, L-glutamine, L-alanine and L-threonine (Fig. 3a). This observation is consistent with the decreases in amino acid metabolism observed under N-limited conditions^39^. A conserved increase in the abundance of caffeic acid was detected in both maize and sorghum in response to N-deficit conditions, which is consistent with previous reports from rice grown under similar N-limited conditions^40^ (Fig. 3a).

All three species exhibited decreases in 1,3-diaminopropane and allantoin abundance under N-deficit conditions (Fig. 3a). Allantoin acts as a pool of relocalizable N that can be catabolized into ammonia for N assimilation and amino acid biogenesis^41^. In addition, the abundance of both succinic acid and maleic acid (MaA) increased in all three species in response to N-deficit stress (Fig. 3a). Maleic acid is produced and secreted in response to another abiotic stress (drought) in holm oak (*Quercus ilex*)^42^. As we examined internal metabolite abundance but did not profile root exudates in the current study, it is not possible to determine whether the internal accumulation of maleic acid resulted in additional secretion in these three grass species. Succinate, the anion of succinic acid, forms part of the tricarboxylic acid (TCA) cycle. The increase in succinic acid levels, combined with the decreased abundance of gamma-aminobutyric acid (GABA), is consistent with these species employing the GABA shunt pathway, which was proposed to act as an additional energy source to support cellular metabolism under stress conditions^43–45^.

We observed changes in metabolite abundance in maize and sorghum grown under P-deficient conditions relative to the nutrient optimal conditions, including L-asparagine, GABA, L-glutamine, L-alanine, capric acid, D-glucose-6-phosphate and glycerol-1-phosphate. However, none of these metabolites exhibited significant changes in abundance in paspalum plants grown under P-deficient vs. nutrient optimal conditions (Fig. 3b). The abundance of D-glucose-6-phosphate (D-G6P), the primary entry molecule for glycolysis was significantly lower in maize and sorghum plants grown under P-deficient conditions vs. those grown under nutrient-sufficient conditions (Fig. 3b). The reduction in D-G6P level might reflect the lack of free phosphate available to produce adenosine triphosphate (ATP) to drive the phosphorylation of glucose, as P is a major component of ATP. The abundance of D-G6P did not decrease in paspalum plants grown under P-deficient conditions (Fig. 3b). None of the metabolites that exhibited significant changes in abundance in paspalum between nutrient optimal and P-deficient conditions, including tryptophan, xylose, glyceric acid, and trehalose exhibited changes in abundance in maize or sorghum (Fig. 3a–d). The abundance of trehalose, a di-saccharide that predominantly functions as a signaling molecule in plants in response to abiotic stresses, was significantly higher in paspalum plants grown under N-deficient or P-deficient conditions vs. the nutrient-sufficient (Full) conditions, but this difference was not observed in maize or sorghum (Fig. 3c, d).

### Conserved and differential transcriptomic responses of paspalum to nutrient-deficit conditions

The sequencing, assembly, and annotation of the paspalum genome provided the opportunity to quantify differences and commonalities in how maize, sorghum, and paspalum transcriptionally respond to nutrient deficit stress. We collected RNA from the root tissues of three biological replicates of each species and used it to generate an average of ~40 million high-quality reads per sample. Principal component analysis based on the transcriptomes of each sample showed a clear separation based on growth conditions in maize (Supplementary Fig. 5a), sorghum (Supplementary Fig. 5b) and paspalum (Supplementary Fig. 5c). We identified 3057, 3144, and 2723 genes with significantly differential expression levels between Full and -N stress conditions in maize, paspalum, and sorghum, respectively. In addition, 591, 2318, and 1698 genes showed significantly differential expression levels between Full and -P stress conditions in maize, paspalum, and sorghum, respectively (Fig. 3e).

Most differentially expressed genes (DEGs) identified for each treatment in each species were themselves syntenically conserved (Fig. 3e). Members of a number of paspalum specific expanded gene families showed significant transcriptional responses to N-deficit stress (Supplementary Fig. 6a) and/or P-deficit stress (Supplementary Fig. 6b). However, consistent with a previous study of transcriptional responses to abiotic stress^46^, the conservation of transcriptional responses was much less common than the conservation of the genes themselves; syntenic genes that showed significant fold changes varied across the three species under N-deficient and P-deficient conditions (Fig. 3f, g).

The set of 220 syntenically conserved orthologous gene groups that responded transcriptionally to N-deficit stress in a consistent fashion among maize, sorghum, and paspalum was disproportionately enriched in GO terms related to response to nutrient levels, nitrate assimilation, metal ion transporter activities and divalent inorganic cation transmembrane transporter activity (Fig. 3f; Supplementary Fig. 7a). The set of 37 syntenically conserved orthologous gene groups that responded transcriptionally to P-deficit stress in a consistent fashion among the three grasses was disproportionately enriched in GO terms related to lipid metabolic process, phosphate ion transport, response to nutrient levels and cell communication (Fig. 3g; Supplementary Fig. 7a). Syntenically conserved orthologous gene groups where a transcriptional response to N-deficit stress was unique to paspalum were enriched in genes involved in proton transport, glycoside biosynthetic process and serine family amino acid metabolic process (Fig. 3f; Supplementary Fig. 7b). By contrast, the syntenically conserved orthologous gene groups that were uniquely differentially expressed in paspalum in response to P-deficit stress were involved in processes related to antioxidation, gene regulation and primary metabolism (Fig. 3g  Supplementary Fig. 7b).

The significant accumulation of trehalose in paspalum in response to nitrogen-deficient conditions motivated us to examine the expression of genes involved in the trehalose metabolic pathway including the genes encoding enzymes that catalyze three steps in trehalose metabolism: trehalose-6-phosphate synthase, trehalose-6-phosphate phosphatase, and trehalase (Fig. 3h). Two maize genes encoding trehalose6-phosphate synthase 1 and 12 are syntenic homeologs resulting from the maize whole-genome duplication are co-orthologous to single gene copies in sorghum and paspalum. These genes formed a clade sister to the well characterized *Arabidopsis thaliana* gene *AtTPS1*^47^ which is consistent with the previous study that characterized *ZmTPS1*^48^ (Supplementary Fig. 8a). Copies of this gene in both sorghum and paspalum showed a significant increase in expression level in response to N-deficient treatment, as did the maize gene encoding trehalose6-phosphate synthase 1 (*ZmTPS1*), which possesses all catalytic domains of TPS (Supplementary Fig. 8b).

Plots of the detectable transcriptional responses of other trehalose-6-phosphate synthase homologs are shown in Supplementary Fig. 8c–i. Genes annotated as encoding trehalose-6-phosphate phosphatase 6 (*ZmTPP6*) and trehalose-6-phosphate phosphatase 11 (*ZmTPP11*) were phylogenetically clustered with Arabidopsis *AtTPPA* (Supplementary Fig. 9a), and both tended to be differentially expressed between control and stress conditions in all three species. Similar transcriptional responses of homologs encoding other trehalose-6phosphate phosphatases were observed across all three species (Supplementary Fig. 9c–i). Trehalase, an enzyme that breaks trehalose down into two molecules of glucose, is encoded by a single gene copy in maize, with conserved syntenic orthologs in sorghum and paspalum. Trehalase encoding gene (*tre1*) in paspalum exhibited a lower level of expression than its syntenic orthologs in sorghum or maize (Fig. 3i).

### Inhibiting trehalase activity in maize and sorghum recapitulates the paspalum phenotype

As shown above, paspalum exhibited a significant accumulation of trehalose in response to the two types of nutrient-deficit stress while maize and sorghum did not (Fig. 3c, d). However, as equivalent P-deficient treatments introduced notably smaller changes in P abundance in paspalum relative to maize and sorghum, and N-deficient treatments produced larger changes, we elected to focus exclusively on N-deficit stress in all subsequent experiments.

**Fig. 4:**
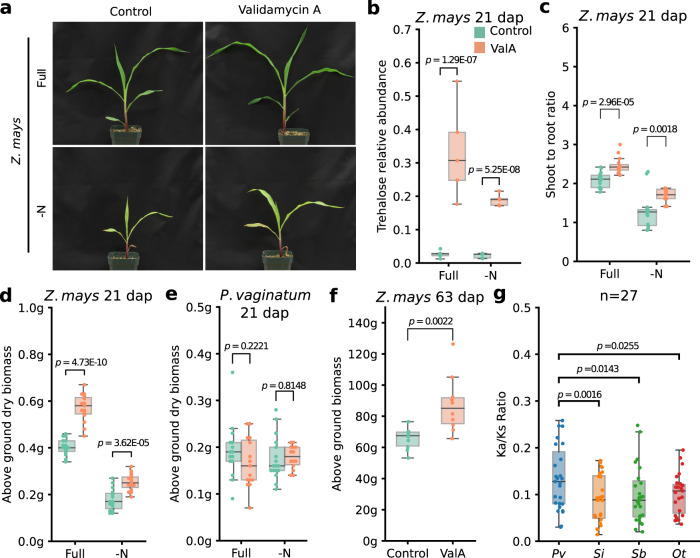
Validamycin A treatment is associated with increased trehalose accumulation and greater biomass production in maize. **a** Representative images showing maize seedlings at 21 days after planting (dap) grown under Full and -N conditions combined with validamycin A treatment (ValA) or without (Control). **b** Changes in trehalose abundance in root tissue in response to validamycin A treatment across conditions. *N* = 5 biological replicates each consisting of pool tissue. **c** Changes in the ratio of shoot-to-root dry biomass in 3-week-old maize seedlings grown across treatments and conditions. *N* = 15 independently measured plants grown across three distinct experiments. **d**, **e** Changes in the above ground dry weight of three week old maize and paspalum plants across treatments and conditions. *N* = 15 independently measured plants grown across three distinct experiments. **f** Biomass accumulation of ValA treated and control maize plants at the late vegetative stage. *N* = 10 independently measured plants. **g** Distribution of observed Ka/Ks (the ratio of nonsynonymous to synonymous substitution rates) for a set of 27 trehalose metabolism genes syntenically conserved across paspalum (*Pv*, *Paspalum vaginatum*, blue), foxtail millet (*Si*, *Setaria italica*, orange), sorghum (*Sb*, *Sorghum bicolor*, green), and Oropetium (*Ot*, *Oropetium thomaeum*, red). Statistical comparisons were conducted using two-sided Wilcoxon signed-rank test. Box plots shown in panels **b**–**g** are defined as in the Fig. 1 legend. Source data are provided as a Source Data file.

In an attempt to phenocopy the reduced plasticity in response to N-deficient treatment originally observed in paspalum, we treated maize and sorghum plants with validamycin A (*β*-d-glucopyranosilvalidoxylamine, ValA)—a specific inhibitor of trehalase activity^49–51^. A visible increase in growth was observed under N-deficit and control conditions among maize plants treated with ValA relative to untreated plants (Fig. 4a). Sorghum plants treated with ValA exhibited a modest but visible increase in growth and biomass accumulation under nitrogen-deficient conditions (Supplementary Fig. 10a). No obvious growth changes in paspalum under either conditions were observed (Supplementary Fig. 10b). Metabolic profiling of treated and untreated plants confirmed that a treatment with 30 µM ValA significantly increased the accumulation of trehalose under both Full and N-deficient nutrient conditions in maize and sorghum (Fig. 4b; Supplementary Fig. 10c) but failed to increase trehalose accumulation in paspalum (Supplementary Fig. 10d).

Nutrient-deficit stress is known to alter shoot-to-root biomass ratios, increasing root biomass as a percentage of the total biomass^52,53^. Root biomass made up a smaller proportion of the total biomass for both maize and sorghum seedlings treated with ValA than untreated seedlings under both Full and N-deficient conditions (Fig. 4c; Supplementary Fig. 10e). However, no significant changes in shoot-to-root ratio were observed in paspalum upon ValA treatment irrespective of nutrient conditions (Supplementary Fig. 10f).

Maize seedlings treated with ValA accumulated greater amounts of dry biomass under both N-deficit and full-nutrient conditions (Fig. 4d). ValA treated sorghum seedlings accumulated more dry biomass under N-deficit conditions than untreated sorghum seedlings but not significant difference was observed under full-nutrient conditions (Supplementary Fig. 10g). In contrast, ValA treatment did not significantly alter biomass accumulation in paspalum under either treatment (Fig. 4e). To extend our observations beyond the late seedling stage, we grew a cohort of maize plants for 63 days (until the late vegetative stage) under either control or ValA treated conditions. ValA treated plants accumulated significantly more biomass than control plants grown as part of the same experiment (control mean = 65.6 grams/plant, ValA mean = 87.3 grams/plant; *p* = 0.002; *t*-test; two tailed) (Fig. 4f). In a preliminary experiment, a smaller number of maize plants were grown under either control or ValA treated conditions to reproductive stage (Supplementary Fig. 11a, b). ValA treated plants flowered earlier (Supplementary Fig. 11c) and produced larger tassels (Supplementary Fig. 11b, d) and leaves than their untreated siblings (Supplementary Fig. 11e). In previous studies, genetically modifying trehalose metabolic pathway altered photosynthesis and nutrient partitioning in maize reproductive tissues, thereby positively affecting yields, via its effect on SNRK1 activity^54,55^. However, in the current smaller scale experiment, ValA induced differences in above ground biomass accumulation, including both tassels and ear shoots as well as vegetative tissues at the reproductive stage, were not statistically significant (Supplementary Fig. 11f). This difference may be explained by an earlier transition to reproductive development in ValA treated plants, but could also be explained by the limited statistical power resulting from limited replication. Moreover, a set of 27 genes associated with trehalose metabolism exhibited significantly more rapid rates of protein sequence evolution in paspalum (*Pv, Paspalum vaginatum*) than did the orthologs of these same genes in foxtail millet (*Si, S. italica*, *p* = 0.002), sorghum (*Sb, S. bicolor*, *p* = 0.014) and *Oropetium* (*Ot, O. thomaeum*, *p* = 0.025) (Fig. 4g). Given the previously reported roles of trehalose metabolism in stress mitigation in plants^56–61^, these data are consistent with, but not conclusive evidence for, selection action on trehalose metabolism in paspalum, whether as a result of the low nutrient abundance of the sandy soils where the species typically grows, the high salinity of the water in the place the species grows, the many other abiotic stresses to which paspalum exhibits high tolerance such as drought, cold, heat and crude oil contamination^20–23,28,56^, or some other unknown selective pressure acting on paspalum but not on the majority of other grasses tested.

### ValA treatment is associated with increased autophagy and nitrogen metabolism in maize

**Fig. 5:**
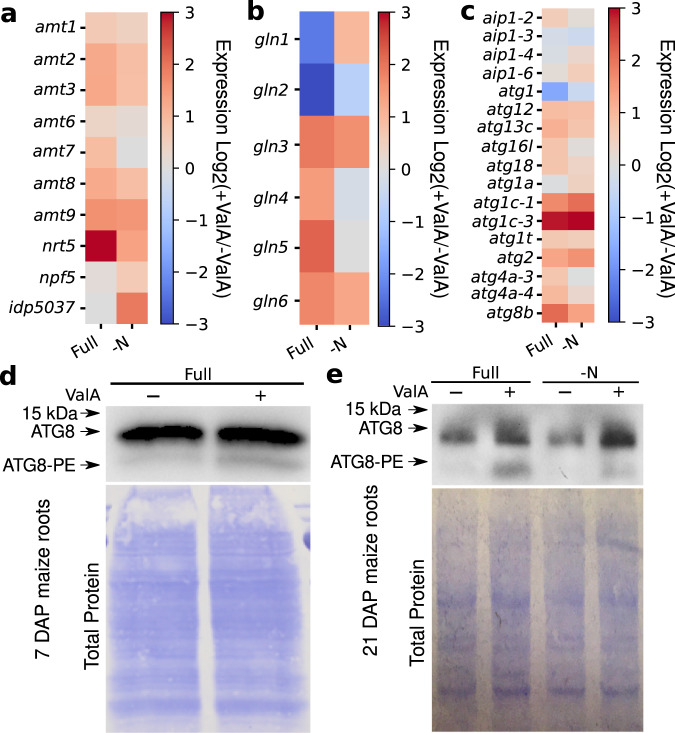
Evidence for increased autophagy in maize seedlings treated with validamycin A. **a** Change in the expression of genes encoding ammonium (*amt1*, *amt2*, *amt3*, *amt6*, *amt7*, *amt8*, *amt9*) and nitrate (*nrt5*, *npf5*, *idp5037*) transporters in response to ValA treatment. **b** Change in the expression of genes encoding glutamine synthetase root isozymes (*gln1*,*gln2*,*gln3*,*gln4*,*gln5*, *gln6*) in response to ValA treatment. **c** Change in the expression of genes related to autophagy in response to ValA treatment. **d** Abundance of free ATG8 (upper band) and ATG8-PE conjugate (lower band) in the roots of one week old maize plants measured via immunoblot. Total protein loading control is shown in the lower panel. **e** Abundance of free ATG8 (upper band) and ATG8-PE conjugate (lower band) in the roots of three week old maize plants measured via immunoblot. The assays shown in panels **d** and **e** were repeated twice with consistent results. Biological replicates of panels **d** and **e** are shown in Supplementary Fig. 13. kDa = kiloDaltons. Source data are provided as a Source Data file.

In order to better understand the changes occurring in maize seedlings treated with validamycin A, gene expression was quantified in ValA treated and untreated seedlings grown under both N-deficient and full-nutrient conditions. In agreement with the observation that genes encoding ammonium transporters and glutamate synthetase were significantly upregulated (Fig. 5a, b), genes annotated as involved in autophagosome assembly, genes annotated as having ATG8 activating enzyme activity, genes annotated as ammonium transporters and genes annotated as playing roles in antioxidation were found to be over represented among genes significantly upregulated in response to ValA treatment (Supplementary Fig. 12a, b). These results suggested at least three potential mechanisms to explain the increased biomass accumulation of ValA treated plants: increased nitrogen uptake resulting from increased expression of nitrogen transporters, increased nitrogen recycling as a result of increased autophagy activation, and/or the amelioration oxidative stress caused by nitrogen deficiency. While increases expression of nitrogen transporters does not help plants in a completely nitrate and ammonium free environment one could plausibly imagine that in nitrogen-constrained but not nitrogen-starved conditions upregulation of ammonium and nitrate transporters might allow plants to obtain sufficient N from lower abundance substrates.

SNRK1 is an upstream promoter of autophagy^62–66^ and more rapid turnover of damaged or unneeded cellular components and proteins allows for more growth with a fixed quantity of N supply. The precursor to trehalose, trehalose-6-phosphate, has been shown to regulate cell growth by inhibiting SNRK1 activity^67–70^. Hence, one potential model to explain the observed result is that treatment with ValA, which inhibits trehalase activity and increases trehalose accumulation^49^ might lead to change of trehalose-6-phosphate. It has been shown that feeding Arabidopsis with trehalose caused downregulation of TPP and rapid trehalose-6-phosphate accumulation^68,71^. Although downregulation of TPS1 was observed in ValA treated seedlings relative to untreated seedlings, no uniform patterns of change in expression levels of these genes were observed in the high copy number of the gene families encoding TPS and TPP in maize genome (Supplementary Fig. 13a). Two maize genes encoding the SNRK1 catalytic subunit (Zm00001d038745 & Zm00001d028733)^72^ both exhibited significant increases in expression in ValA treated seedlings relative to untreated controls under both N-deficit and Full condition (Supplementary Fig. 13b, c). SNRK1 has been shown to repress malate dehydrogenase genes in maize^62,73^ so upregulation of SNRK1 would be predicted to result in a downregulation of malate dyhydrogenases. Both *ZmMDH3* (Zm00001d044042) and *ZmMDH6* (Zm00001d031899) were significantly downregulated in ValA treated seedlings related to untreated seedlings (Supplementary Fig. 13d, e). The expression of many known autophagy-related genes were also higher in ValA treated seedlings than in untreated seedlings (Fig. 5c).

An alternative mechanism by which trehalose accumulation could activate autophagy is by the inhibition of Solute Carrier family glucose transporters, resulting in SNRK1 activation, as has been previously reported in mammalian systems for AMPK, the animal equivalent of SNRK1^74^. All four non-chloroplast SLC glucose transporter genes showed statistically significant changes in expression in our expression dataset with the expression of *SLC2C-8a* (Zm00001d009603) increasing (Supplementary Fig. 13f) in response to ValA treatment and the expression of other three decreasing (*SLC23-2* (Zm00001d012693), *SLC2C-8* (Zm00001d029254) and *SCL35F1* (Zm00001d038299)) (Supplementary Fig. 13g). During autophagy, the protein ATG8 becomes conjugated to phosphatidylethanolamine (PE) of which the abundance is a promising indicator of autophagy activity^75–77^. In maize, N-deficit stress did not produce any obvious change in the accumulation of either free ATG8 or ATG8-PE; however, under both control and N-deficit stress conditions, two independent replicates of plants treated with ValA accumulated more free ATG8 and more ATG8-PE than untreated controls (Fig. 5d and Supplementary Fig. 13h). Similar results were observed in two independently replicates of one week-old-seedlings, a stage where no differences in growth were yet apparent between treatments and seedlings are expected to still be dependent on nutrients from the seed for growth (Fig. 5e and Supplementary Fig. 13i, j). We did not observe accelerated rates of protein sequence evolution in a set of 23 autophagy-regulated genes conserved at syntenic locations across the genomes of paspalum, foxtail millet, sorghum and oropetium (Supplementary Fig. 14a), although changes in protein sequence evolution for these genes would not necessarily be expected in the event tehalose induced increases also play a role in the low phenotypic plasticity of pasplaum under nutrient deficient treatments as observed for increased growth under nutrient-limited conditions in maize. Ultimately, the experiments conducted in this study are relevant to maize and suggestive, but not conclusive with regards to paspalum.

### Validamycin A induced increases in biomass accumulation in maize is autophagy dependent

**Fig. 6:**
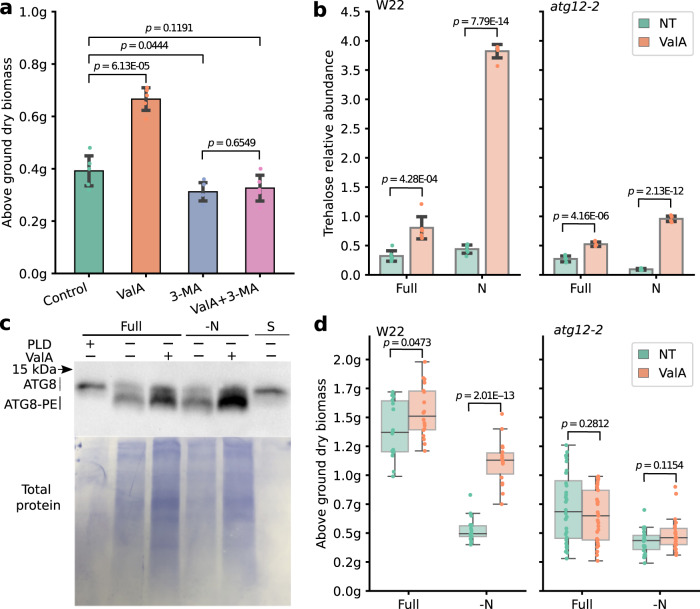
Validamycin A induced biomass productivity is dependent on trehalose-mediated autophagy. **a** Accumulation of above ground dry biomass for 3-week-old control seedlings (green), seedlings treated with 3 mM 3-MA (3-methyladenine) (orange), 30 µM ValA (purple) or both 3 mM 3-MA and 30 µM ValA (pink) measured in grams (g). (*N* = 5 individual plants). **b** Accumulation of trehalose in W22 and homozygous *atg12-2* mutant seedlings across treatments and conditions (*N* = 6 individual plants). Error bars in panels **a** and **b** indicate standard deviation. For panels **a**, **b**, statistical significance between indicated comparisons were determined by two tailed *t*-test. **c** Change in the abundance of free ATG8 (upper band) and ATG8-PE conjugate (lower band) in the roots of three week old maize plants treated with ValA relative to control plants measured via immunoblot. Total protein loading control is shown in the lower panel. PLD: phospholipase D treatment which digests ATG8-PE conjugation. S: Cellular soluable fraction separated by ultracentrifugation. The assay shown in panel **c** was repeated twice with consistent results. **d** Changes in the above ground dry weight, measured in grams (g), of W22 and homozygous *atg12-2* seedlings at 21 days after planting in response to validamycin A (ValA) treatment (*N* = 15 independent plants measured across three separate experiments). All box plots shown in panel **d** are defined as in the Fig. 1 legend. kDa = kiloDaltons. Statistical comparisons were conducted using two-sided Wilcoxon signed-rank test. Source data are provided as a Source Data file.

Changes in expression in ValA treated seedlings were consistent with increases in nitrogen uptake via upregulation of transporter proteins, reductions in oxidative stress caused by nitrogen deficit via upregulation of anti-oxidation pathways and increases in nitrogen recycling via increased activation of autophagy (Fig. 5a and Supplementary Fig. 12). To determine whether the apparent increase in autophagy in ValA treated seedling was necessary to produce the observed increase in biomass, we employed 3-methyladenine (3-MA) a phosphatidylinositol 3-kinase (PI3K) inhibitor that prevents autophagosome formation^77,78^. Seedlings treated with 3-MA along exhibited modest reductions in growth relative to untreated plants. Seedlings treated with ValA alone, as previously observed, exhibited substantial increases in biomass accumulation. However, in the presence of 3-MA, no significant increase in biomass accumulation was observed in response to ValA treatment relative to either untreated seedlings or seedlings treated with 3-MA alone (Fig. 6a). These results were consistent with autophagy activity playing an essential role in the increased biomass accumulation observed in ValA treated maize plants that accumulate greater quantities of trehalose.

To further investigate the role of autophagy in ValA mediated physiological changes, we employed a maize line homozygous for *atg12-2*, a *Mu* insertion allele of *atg12* (Supplementary Fig. 14b)^79^. ATG12 forms a ligase complex with ATG5 and ATG16 that is essential for the lipidation of ATG8^79–81^. In *atg12-2* mutant background, maize genes encoding TPS are predominately downregulated in response to N-deficient condition, while in wild-type controls, several of the same genes exhibited increased expression in response to N-deficient treatment while genes encoding TPP showed varying responses to both N-deficit stress and genetic background (Supplementary Fig. 14c). This observation is consistent with reports from Arabidopsis where defects in autophagy globally down-regulate carbon metabolism^79,82^. It should be noted that, in contrast to the experiments reported above utilizing the maize reference genotype B73, both wild-type and mutant *atg12-2* plants were evaluated in a W22 genetic background. Three independent experiments were performed to measure internal trehalose accumulation, autophagy activity and biomass accumulation of the wild-type background W22 and *atg12-2* under Full and -N conditions with or without validamycin A treatment. Consistent with results shown above for B73, validamycin A treatment led to significant increase of trehalose abundance in both wild-type W22 and *atg12-2* seedlings (Fig. 6b). Higher abundance of both free ATG8 and ATG8-PE were observed in ValA treated wild-type seedlings relative to untreated controls in both nitrogen-deficient and full-nutrient conditions in two independent biological replicates (Fig. 6c and Supplementary Fig. 14d). Wild-type W22 seedlings, like B73, showed significant larger accumulations of biomass when treated with ValA relative to untreated seedlings under both Full and -N conditions (Fig. 6d). In contrast, in *atg12-2* mutants, no significant difference was detected in biomass accumulation in response to ValA treatment (Fig. 6d). These data together suggested that the enhanced growth and biomass accumulation of maize observed in ValA treatment maize seedlings accumulating trehalose is indeed autophagy dependent.

## Discussion

In paspalum, a crop wild relative that is resilient to numerous abiotic stresses, nutrient-deficit stress was associated with substantial accumulation of trehalose. The sequencing of a reference genome for this species allowed us to perform comparative evolutionary analyses, which identified accelerated protein sequence evolution of genes involved in trehalose metabolism in paspalum (Fig. 4g). Treating maize and sorghum exposed to N-deficit stress with a specific inhibitor of trehalase resulted in higher internal trehalose accumulation and recapitulated a number of paspalum phenotypes including improved growth and biomass accumulation under N-deficient conditions relative to untreated controls, and increased allocation of biomass to shoots under N-deficient conditions (Fig. 4 and Supplementary Fig. 10).

Imposing equivalent stress treatment protocols across species presents numerous challenges. One potential concern with the initial finding that paspalum is less phenotypically plastic in response to nutrient-deficient treatment than maize is that the slower baseline accumulation of biomass in paspalum may deplete the modest reserves of nitrate and phosphate in soil more slowly than they would be used by maize. Here comparison of paspalum to sorghum may be more informative than comparison of paspalum to maize. Under nutrient replete conditions, individual paspalum ramets accumulated approximately equivalent amounts of biomass to sorghum seedlings, while sorghum exhibited greater declines in growth in response to nutrient deficit stress than did paspalum. In addition, three lines of evidence indicate that the three species experienced nutrient deficit stress in response to the nutrient deficient treatment protocols employed in this study: significant declines in the abundance of N and P in the above ground tissue of all three species when grown under N- and P-deficient conditions (Fig. 2e, g); the depletion of the nitrate storage compound allantoin under the N-deficit conditions as well as other metabolic changes (Fig. 3a, b); and the enrichment of N or P assimilation/metabolism among the core genes responding to N or P deficient conditions across all three species (Supplementary Fig. 7).

In many flowering plant species, the abundance of trehalose is quite low^83^. In Arabidopsis, trehalose accumulation was observed under high and low-temperature stress^84,85^, high light intensity^86^, high cadmium levels^87^ and dehydration^88^, but no increase was observed under N-deficient conditions^83,89^. A non-targeted metabolic profiling of six legume plants in the *Lotus* genus under drought conditions revealed a significant increase in trehalose abundance across all species tested^90^. The transgenic expression of trehalose-6-phosphate phosphatase in developing maize ears was associated with increased yield under control and drought-stressed conditions^55^. Genetically modified rice plants that over-expressed a fusion gene encoding both *Escherichia coli* Trehalose-6-phosphate synthase and Trehalose-6-phosphate phosphatase, which are responsible for trehalose biosynthesis, exhibited 200-fold greater accumulation of trehalose and significantly higher tolerance to drought, salinity, and cold stress^60^.

Associations and external applications of trehalose under nutrient-deficit stress appear to have been less investigated. One study found that *Nicotiana tabacum* accumulates more trehalose under N-deficient conditions and the exogenous foliar application of trehalose partially rescued the N-deficiency phenotypes by upregulating nitrogen metabolism throughout the plants^91^. In maize, external trehalose treatment enhanced antioxidant activities under high salinity and P-deficient conditions, thus achieving better seedling growth^92^. Paspalum is adapted to high salinity environments^19,29,93^ and trehalose accumulation in paspalum may also act to ameliorate osmotic stress caused by a larger amount of salt uptake from the soil driven by a higher transpiration when nutrient-deficient plants seek to increase nutrient uptake.

Gene expression data collected from maize seedlings treated with ValA as well as untreated seedlings under both full-nutrient and nitrogen-deficient conditions identified an enrichment of genes annotated as playing roles in different biological process such as regulation of DNA binding, cell junction organization, nitrogen uptake, nitrogen metabolism, and autophagy among genes expressed to significantly higher levels under ValA treatment (Supplementary Fig. 12). Several studies have reported that trehalose can activate autophagy in both animals and plants, including mTOR-independent activation in animals^65,94,95^. ValA treated maize seedlings contained greater amounts of ATG8 protein as well as greater amounts of ATG8 lipidation both at three weeks and one week after planting (Fig. 5d, e). Autophagy plays pivotal roles in proteome remodeling, lipid turnover^65,79^, nitrogen remobilization^96–99^, nitrogen use efficiency^100^, and abiotic stress responses in a variety of plant species (as reviewed previously^101^). In the resurrection plant *Tripogon loliiformis*, trehalose abundance was correlated with an increase in ATG8 lipidation and the number of autophagosomes during desiccation to prevent cell death^76^. It also has been shown that autophagy promotes biomass productivity and nitrogen use efficiency in rice during vegetative stage^100^.

We explored several potential mechanisms that could explain the observed ValA effect. One of them could be elevated SNRK1 activity which activates autophagy^65^. Gene expression data collected in this study includes a number of lines of evidence consistent with increased SNRK1 activity including increases expression of SNRK1 subunits and decreased expression of genes known to be repressed by SNRK1 (Supplementary Fig. 13b–e). However, other previous studies can also provide alternative mechanisms for trehalose-induced SNRK1 activity. One example is that in certain human cell types trehalose inhibits glucose import via SLC2A (aka GLUT) transporters, resulting in the induction of the mammalian homolog of SNRK1, adenosine monophosphate–activated protein kinase (AMPK) dependent autophagy^65,74,102^. Three SLC glucose transporters exhibited significant decreases in expression in seedlings treated with ValA relative to untreated seedlings (Supplementary Fig. 13f, g) consistent with a potential alternative explanation for SNRK1 activation by trehalose accumulation (Supplementary Fig. 14e).

Caution should be taken in interpreting these results as multiple genes encoding enzymes in the trehalose biosynthetic pathway were also reported to be associated with changes in autophagy activity in different systems^67,103^. Trehalase activity was initially observed in tissue cultures generated from a range of plant species more than three decades ago^104^. Inhibiting trehalase activity with ValA can control wheat *Fusarium* head blight (FHB) and inhibit Deoxynivalenol (DON) contamination^105^. Validamycin A was also proposed to be a promising fungicide or insecticide due to its inhibitory effect on trehalase^106^. Over-expression of *OsTRE1* in rice was associated with improved salt tolerance^107^ and over-expression of *AtTRE1* in Arabidopsis was associated with improved drought tolerance^108^. However, these known phenotypic consequences of alterations in trehalase activities would not necessarily predict an association between inhibition of trehalase activity and decreased plasticity in response to nutrient deficiency stress, as was observed here.

Whatever the mechanism responsible for the association between increased trehalose accumulation and increased autophagy in ValA treated plants, we sought to evaluate whether this apparent increase in autophagy was necessary for the observed increase in biomass accumulation observed in the same plants. Genes encoding ammonium and nitrate transporters were also more expressed in seedlings treated with validamycin A under both nitrogen-deficient and full-nutrient treatments (Fig. 5a, b) and increased nitrogen uptake was a potential alternative explanation for the observed increases in biomass accumulation. Pharmaceutically inhibiting autophagy via treatment with 3-MA (3-methyladenine) abolished the effect of ValA treatment on biomass accumulation (Fig. 6a) consistent with autophagy activity being necessary for the increased biomass accumulation observed in maize seedlings treated with ValA. On the other hand, the biomass reduction under 3-MA treatment could be explained by its inhibitory effect on PI3K such as the Target of Rapamycin (TOR) given that a slight but statistically significant reduction of growth of the wild-type seedlings treated with 3-MA was observed relative to untreated seedlings (Fig. 6a). The effect of ValA treatment was also evaluated in plants carrying a *Mu* insertion in *atg12*, a core component of the autophagy pathway^79,96^. Plants homozygous for *atg12-2* failed to exhibit increased growth in response to ValA treatment under both nitrogen-deficient and Full condition but still accumulated greater amounts of trehalose in ValA treated plants than untreated plants (Fig. 6b–d). The combination of chemical inhibition and mutant data confirm that autophagy is necessary for the increased growth and biomass accumulation observed in maize seedlings accumulating greater trehalose—mirroring the increased trehalose observed in paspalum under nitrogen-deficient conditions. The reversion of validamycin A treated WT seedlings to un-treated levels of biomass accumulation in the presence of an autophagy inhibitor or in autophagy mutants suggests that the role of increased nitrogen uptake, if any, is likely also autophagy dependent.

Previous reports have demonstrated the potential to increase agricultural productivity per unit of nitrate and phosphate fertilizer applied via the manipulation of the trehalose pathway^55,58,91,109^. Our results are consistent with these reports and providing evidence that increased autophagy activity explains some or all the observed increases in biomass accumulation under nutrient-limited conditions and specifically suggest the modification of trehalase activity, whether via chemical or genetic methods is an approach that is likely to be well tolerated by maize and sorghum. However, increased autophagy would not be expected to produce increases in biomass accumulation in plants where growth is not nutrient limited. In the pattern of reduced phenotypic plasticity in response to nitrogen deficit observed in wild-type paspalum and ValA treated sorghum would both be consistent with this mechanism. However, in ValA treated maize, biomass accumulation increased in both nitrogen-limited and full-nutrient conditions. One potential explanation is the rapid growth of maize seedling relative to the other two species examined in this study, fueled by the larger starch reserves of the maize kernel, exceed the early maize seedling’s capability for nitrate uptake, creating nitrogen-limited growth even in nitrogen repleted soils. However, for the moment this explanation remains purely speculative.

The maize experiments described in this paper would not have been conducted in the absence of the observation that paspalum accumulates trehalose in response to nutrient-deficient treatments. At the same time, the work linking trehalose accumulation to increased biomass accumulation via an autophagy-dependent mechanism was conducted entirely in maize. Strong tests of whether or not the mechanism observed in maize does indeed explain the limited response of paspalum to nutrient deficit will likely require new approaches such as the development of a transformation protocol for paspalum which would enable overexpression of the trehalse enzyme in that species. Until that time, it should be noted that the data presented here from maize experiments did not provide conclusive evidence that the same mechanism acts in paspalum. In any case, these results suggest that the manipulation of trehalose accumulation in maize and sorghum, as well as potentially on other domesticated grasses, whether chemically or via the modification of the expression of the endogenous trehalase enzyme, may increase agricultural productivity per unit of nitrate and phosphate fertilizer applied. Finally, the observation of autophagy-dependent increases in biomass accumulation in even maize plants grown under nutrient-replete conditions suggests that current maize lines may exhibit a suboptimal level of autophagy in roots. However, again, caution should be taken in interpreting these results as, while increases in biomass accumulation were observed not only in seedlings but in late stage vegetative plants (Fig. 4f), all data presented in this study were generated in controlled environmental conditions and changes in regulation or metabolism that are beneficial in the greenhouse may or may not generalize to the field.

## Methods

### Genome size estimation via flow cytometry and genome size estimation

One leaf per plant of paspalum (PI 509022) and sorghum (BTx623) were harvested and kept on ice until processing. A CyStain Propidium Iodide Absolute P kit (Sysmex, Milton Keynes, United Kingdom) was used to extract and stain the nuclei from a 1 cm^2^ piece of leaf tissue following the manufacturer’s instructions. To reduce the amount of cellular debris in the extracts, samples were passed through a 30 µm filter (CellTrics®-Sysmex Partec, Goerlitz, Germany) and centrifuged at 600 × *g* before final staining. Sorghum was used as an internal standard to reduce the staining variability between samples. The stained samples were then analyzed on a CytoFLEX flow cytometer (Beckman Coulter, Brea, CA, USA) following a two-hour incubation at 4 °C. The propidium iodide was excited with a yellow-green 561 nm laser and detected with a 585/42 emission filter. The genome size was calculated for a total of six samples. The 2C genome size for BTx623 is 1.67 pg DNA^110^; therefore the formula to calculate the DNA content of paspalum was (median fluorescence_sample nuclei_ /median fluorescence_standard nuclei_) × 1.67 pg. The mean and standard error of these six samples were calculated, and the mean was converted to a 1C genome size using the conversion factor 1 pg = 980 Mbp.

### Paspalum genome sequencing, assembly, and annotation

The genome of *Paspalum vaginatum* was sequenced using a whole-genome shotgun strategy and standard. Sequencing reads were generated using both Illumina and PacBio platforms. Sequencing on both platforms was conducted at Department of Energy’s Joint Genome Institute (JGI) in Walnut Creek, California. Illumina reads were generated using the Illumina HiSeq-2500 platform. One 400 bp insert 2 × 150 Illumina fragment library was sequenced (Supplementary Table 3). Prior to assembly, Illumina fragment reads were screened for PhiX contamination. Reads composed of >95% simple sequence were removed. Illumina reads *<*50 bp after trimming for adapter and quality (*q* < 20) were removed. The final read set consists of 266,209,446 reads for a total of 102.4x of high-quality Illumina bases. PacBio sequence data was generated using the SEQUEL I platform. A total of 14 PB chemistry 2.0 chips (10 hour movie time) were sequenced with a sequence yield of 52.01 Gb, with a total coverage of 74.30x with an average read length of 9,523 bp (Supplementary Table 4).

The reads were assembled using MECAT (v1.3)^111^ and polished using QUIVER(v1.1)^112^. This produced an initial assembly of 5358 scaffolds (5358 contigs), with a contig N50 of 771.9 Kb, 1099 scaffolds larger than 100 Kb, and a total assembled size of 838.4 Mb (Supplementary Table 5). Heterozygous snp/indel phasing errors were corrected using the 74.3x raw PACBIO data. A total of 102,526 (4.2%) of the 2,317,409 heterozygous SNPS/InDels were corrected. Finally, homozygous SNPs and InDels were corrected in the release consensus sequence using 78x of Illumina reads (2 × 150, 400 bp insert) by aligning the reads using bwa mem^113^ and identifying homozygous SNPs and InDels with the GATK’s UnifiedGenotyper tool^114^. A total of 1591 homozygous SNPs and 233,333 homozygous InDels were corrected in this process. Two genetic maps totaling 7680 markers^30^ (provided by the Katrien Devos group, University of Georgia), combined with data from the version 2 release of *Panicum hallii var. HAL2* genome were used to identify misjoins in the initial assembly. Misjoins were characterized as a discontinuity in the *P. vaginatum* linkage group. A total of 15 misjoins were identified and resolved. An expanded set of six F_1_ genetic maps including a total of 8861 markers were used to further refine the order/orientation of the contigs for the final pseudomolecules. The resulting sequences were then oriented, ordered, and joined together into 10 chromosomes using both the genetic map (Supplementary Figs. 15–24) and synteny with the *P. hallii* genome. Each chromosome join is padded with 10,000 Ns. Significant telomeric sequence was identified using the (TTTAGGG)n repeat, and care was taken to make sure that it was properly oriented in the production assembly. The remaining scaffolds were screened against bacterial proteins, organelle sequences, GenBank nr and removed if found to be a contaminant. The final chromosome numbering and orientation of the *Paspalum vaginatum* pseudomolecules was set to match *S. bicolor*, using global gene synteny by aligning *S. bicolor* transcripts obtained from Phytozome (https://phytozome-next.jgi.doe.gov/).

The v3.0 paspalum genome assembly described in this manuscript was annotated using a combination of an alignment of assembled transcripts from paspalum and protein sequences from other plant species. Prior to its annotation, the genome assembly was first repeat-masked using both known repeats from RepBase and de novo identified repetitive sequences from RepeatModeler^115,116^. Transcript assemblies were generated via a two-stage assembly process utilizing PERTRAN followed by PASA^117^. A total of 112,258 RNAseq transcript assemblies were generated from ~1.6 billion 2 × 150 bp strandspecific Illumina sequencing reads. Protein sequences from Arabidopsis, soybean, sorghum, Kitaake rice (*Oryza sativa* Kitaake), green foxtail (*Setaria viridis*), grape (*Vitis vinifera*), and the Swiss-Prot proteomes were aligned to the repeat-masked genome using EXONERATE^118^. Independent sets of gene models were predicted using FGENESH+, FGENESH_EST, EXONERATE and AUGUST as implemented in BRAKER1, and the in-house PASA assembly open reading frames (ORFs; in-house homology constrained ORF finder) tool from JGI^118–120^. For each locus, the prediction with the best score based on the expressed sequence tag (EST) and protein support and a lack of overlap with repeats was selected. The best prediction for each locus was further improved using PASA to add untranslated regions, correct splice sites, and add alternative transcripts. Improved transcripts were assessed based on both the C-score (ratio of the BLASTP alignment score to the mutual best hit BLASTP alignment score) and protein coverage. Transcripts were retained if any one of three criteria were met: (1) Transcripts where the C-score and protein coverage score were each ≥0.5 and <20% of the transcript overlapped with sequence annotated as repetitive. (2) Transcripts supported by EST coverage and <20% of the transcript overlapped with sequence annotated as repetitive. (3) Transcripts with a Cscore ≥ 0.9 and a protein coverage score ≥ 0.7, regardless of the proportion of overlap with annotated repeat sequences. Sequences that satisfied one or more of the above three criteria and where more than 30% of predicted protein sequence was covered by Pfam domains annotated as belonging to transposable elements were also removed. Short single exon (predicted coding sequence <300 bp) genes without protein domain support and expression data, incomplete gene models and those with low homology support (sum of Cscore and coverage <1.5 for complete, <1.8 for incomplete) and without full transcriptome support (CDS and intron coverage supported by any transcript assemblies) were removed. Gene models that passed all the criteria described above were included in the gene model annotations for paspalum. The GO terms assignment was based on the InterProScan results^121^.

### Plant materials and growth conditions

The maize (*Zea mays* ssp. *mays*), sorghum (*Sorghum bicolor*), and seashore paspalum (*Paspalum vaginatum*) genotypes used to create the reference genomes for each species were: accessions B73, BTx623, and PI 509022, respectively^122,123^. Maize and sorghum seeds were surface sterilized in 2% bleach for 40 min, rinsed, and imbibed overnight in deionized distilled water (ddiH_2_O). The seeds were sown in a mixture of 20% MitroMix200, 30% sterilized sand and 50% fine vermiculite(v/v) and grown under greenhouse conditions (temperature: 22–29 °C with a 14-h light: 10-h dark photoperiod). The heterozygous reference clone PI 509022 was obtained from the USDA National Plant Germplasm Service and propagated via rhizome cuttings using the same growth medium and conditions used for sorghum and maize. All plants were watered with sterilized ddiH_2_O until three days after emergence (usually 4–5 days after planting). For each trial, three days after emergence, the seedlings were divided evenly into three trial groups. The first group received Hoagland nutrient solution (Supplementary Table 6) and ddiH_2_O on alternating days. The second group received Hoagland nutrient solution in which the potassium nitrate and calcium nitrate were substituted with potassium sulfate and calcium chloride, respectively, to remove nitrate (Supplementary Table 7). The third group received Hoagland nutrient solution in which the monopotassium phosphate was substituted with potassium sulfate to remove phosphate (Supplementary Table 8). Micronutrients solution supplied to all three types of solutions (Supplementary Table 9). The nutrient treatments continued every other day until harvest. For the ValA treatment assay, plants grown under Full-nutrient or N-deficit conditions were treated with 30 µM ValA dissolved in nutrient solutions beginning at 7 days after planting; the plants were treated at 6 PM every other day. For 3-MA (3-methyladenine) treatment experiment, 3 µM 3-MA was applied to plant seedlings at 6 PM at the same days as the ValA treatments were administered. All of date for 3-week seedlings grown under designated conditions were collected from three trials with more than 5 replicates during the period from April to September. For the experiment where the plants reached reproductive stage, data were collected from 1 trial with three replicates. For the experiment where the plants just reached the flowering stage, data were collected from 1 trial with 10 replicates.

### Segregation and purification of *atg12-2* mutants with *Mu* insertion at *atg12* loci

Seeds for a stock carrying the *atg12-2* insertion (stock ID: 532 L + atg12-2) generated as part of the *UniformMu* project^124^ were ordered from maizeGDB^125^. Individual plants were screened for the presence of the *atg12-2* allele and the ones with the homozygous insertion were selfed to generate multiple families. DNA was extracted using the MagMAX® Plant DNA kit by applied biosystems (SKU: A32549) using the standard protocol. To detect the transposon insertion in *atg12* locus, a pair of previously reported primers^96^ LP (TGTACTTCCAAGCTCTTTACCTGAGG) and TIR6 (AGAGAAGCCAACGCCAWCGCCTCYATTTCGTC) were purchased from Europhin Genomics and used to amplify the site which yielded a band with >400 bp in *atg12-2* mutants but not in wild type. Replacing the TIR primer with the previously reported RP primer^96^ (TTAGCCCCATGCTGCCGATAAAGCA), purchased from Europhin Genomics, successfully amplified a >900 bp band in wild-type plants but not in the homozygous *atg12-2* mutant allele. DNA isolated from a known wild-type stock of W22 was employed as a positive control for the LP/RP PCR reaction, and PCR-grade water (Millipore-Sigma® SKU:3315932001) was employed as a negative control for all PCR reactions. The program employed for PCR amplification was as follows: 94 °C for 4 min, 94 °C for 30 s for 32 cycles, 60 °C for 30 s, 75 °C for 90 s, with a final extension for 5 min at 75 °C. GoTaq® reagents were used for the reaction mix. PCR products were separated in an 1% agarose gel after running for 30 min at 100 V. The gel were then imaged in a Bio-Rad imaging system. One of the resulted families from selfed plants was used to perform Nitrogen and ValA experiments (see subsection Plant materials and growth conditions inside materials and methods). To confirm that *atg12-2* plants used in the experiment above were homozygous mutants, we genotyped them using 50 mg of leaf tissue at the harvested point using the exact protocol.

### Plant phenotyping and root sampling

On the date of harvest and phenotyping, the plants were taken to a dark room illuminated solely by green light, separated from the potting media and cleaned in a two stage process. The roots were washed in a 0.05% bleach solution and then were rinsed with warm running water and dried with paper towels. The root samples used for RNA extraction and metabolite analyses were flash frozen in liquid nitrogen. The roots were scanned using an EPSON scanner (Perfection V550, setting at 120 dpi; Epson, Suwa, Japan) with a green film covering the scanning surface to avoid exposing the roots to non-green light. Fresh biomass measurements were taken for the whole seedlings, after which they were divided into shoot and root fractions and weighted separately. Dry weight measurements of shoots and roots were taken after 48 h of freeze-drying. For paspalum, the weight of the original rhizome cutting was subtracted from the final whole-plant fresh biomass to estimate biomass accumulation.

### Species phylogeny construction

A set of 7728 single-copy syntenic orthologs from the *Zea mays*, *Sorghum bicolor*, *Setaria italica*,*Oropetium thomaeum*, *Brachypodium distachyon* and, *Oryza sativa* genomes was extracted from the syntenic gene sets identified among the seven species. Of the 7728 orthologs with primary transcript CDSs longer than 500 bp, 6151 were aligned using the codon-based aligner ParaAT(v2.0)^126^. Subsequently, the 6151 multiple sequence alignments, each consisting of one gene each from each of the seven species were trimmed to remove poorly aligned or highly divergent regions using Gblocks(v0.91b)^127^ with the following parameters: minimum number of sequences for a conserved position set at 5; minimum number of sequences for a flank position set at 6; maximum number of contiguous nonconserved positions set at 8; and minimum length of a block set at 10. The resulting nucleotide alignments were used to construct phylogenies using RAxML(v8.2) with the parameters’-f a -N 1000 -m GTRGAMMA -x 1234 -p 1234’ and a clade containing rice and *Brachypodium* as an outgroup^128^. In 292 cases, it was not possible to form a monophyletic clade containing rice and *Brachypodium*. The remaining 5859 trees were analyzed using DensiTree(v2.2.6) to generate a consensus tree^129^. IQ-TREE(v1.6) was used to construct maximum likelihood phylogeny estimate branch lengths using a super gene concatenated from the trimmed nucleotide sequence alignments of the 5859 single copy syntenic genes used for consensus tree analysis^130^. Divergence time estimates were then performed using these branch lengths, a previously estimated divergence date for *B. distachyon* and *O. sativa* of 54 Myr ago^131^ and an estimated divergence date for *Z. mays* and *S. bicolor* of 12 Myr ago^132^ as a reference with r8s(v1.81) software^133^.

### Syntenic and substitution rate analysis

Syntenic orthologous gene pairs were identified between the sorghum and paspalum genomes using sequence similarity data from LAST(v1170)^134^ and a Python implementation of MCScan, JCVI(v1.0.14)^135,136^. This analysis was run using the command’python -m jcvi.compara.catalog ortholog paspalum sorghum –no_strip_names’. The LAST results were filtered using a Cscore setting of ≥−0.7. Raw synteny gene pairs were polished identifying the single most significant aligning gene within each syntenic interval, an approach found to recover larger numbers of syntenic pairs and reduce the number of cases where adjacent homologous genes were collapsed into into copies^46^. Sorghum-paspalum orthologous gene pairs were merged into a published sorghum referenced synteny list^46^ for maize (B73_RefGen_V4)^122^, sorghum v3.1^123^, foxtail millet v2.2^34^, *Oropetium* v2.0, rice v7^137^, and *Brachypodium* v3.1^138^. The final synteny list and the scripts used to generate it are hosted at https://github.com/xiaoguanghuan123/paspalumGenome. Codon-level multiple sequence alignments of syntenic orthologous gene groups were generated with ParaAT2.0^126^. Synonymous nucleotide substitution rates (Ks), and non-synonymous nucleotide substitution rates (Ka) were estimated from these multiple sequence alignments using the ‘codeml’ package implemented in PAML (v4.7)^139^. The estimation was conducted using the maximum-likelihood method and the parameters runmode = 0, Codon-Freq = 2, model = 1. The known phylogenetic relationships of the six included species were used as a known input tree. Syntenic orthologous groups containing any genes with a Ks >2, a Ka >0.5, and a Ka/Ks ratio >2 were removed.

### Gas chromatography–mass spectrometry (GC-MS) metabolite profiling

Root samples from maize, sorghum, and paspalum seedlings grown as described above were collected in a dark room illuminated solely by a green bulb and ground into a fine powder in liquid nitrogen. Approximately 50 ± 0.5 mg of the ground powder was used for metabolite extraction. Metabolites were extracted with 730 µL of methanol by mixing at 70 °C for 30 min. Following the addition of 750 µL of water and 325 µL of chloroform for phase separation, 50 µL of upper aqueous was dried and derivatized by methoxyamination (37 °C 2 h with 40 µL of 20 mg mL^−1^ methoxyamine hydrochloride in pyridine) and trimethylsilylation (37 °C for 30 min with 70 µL of N-methyl-N-(trimethylsilyl)trifluoroacetamide)^140,141^. A 1 µL sample of the derivatized material was analyzed in splitless mode using a 7200 GC-QTOF system (Agilent Technologies, Santa Clara, CA, USA). A solution of fatty acid methyl esters (C8 to C30) was added to each sample during derivatization to determine the retention index. The raw data were acquired using MassHunter Workstation v.08 (Agilent Technologies), while peak detection, deconvolution and identification were performed using MassHunter Unknown Analysis software (Agilent Technologies) using the Fiehn GC/MS Metabolomics RTL Library (Agilent Technologies) as a reference. Peak areas of the identified metabolites were computed using MassHunter Quantitative Analysis software (Agilent Technologies). Peak area was normalized by the precise sample fresh weight and the peak area of the ribitol added to each sample as an internal standard to calculate the relative levels of metabolites. Trehalose was quantified with the same procedure, and the peak area corresponding to the authentic standard (retention index, 2480; quantitation ion *m/z*, 361.1651) was quantified.

### Genetic map construction for genome assembly validation

Nine genetic maps generated from two populations were employed to order, and orient the scaffolds into pseudomolecules, and to validate the assembly. The first population employed was an F_1_ population of 184 individuals derived from a cross between paspalum accessions PI 509022 and HI33, in Qi et al.^30^. The second population was generated by crossing two F1 sibs from the PI 509022 x HI33 population. Only 52 progeny of this cross were validated and ultimately used for map construction. Essentially the same protocols were used for marker development and genetic mapping in the F2 population, except that the restriction enzymes *PstI* and *MspI* were used for GBS library preparation. SNPs in the F1 population were called from GBS reads both independently of the genome assembly and by alignment to an early draft of the paspalum genome assembly. SNPs in the F2 population were called from GBS reads aligned to seashore paspalum assembly v2.0. Because the mapping software MAPMAKER^142^ does not have an algorithm to deal with outcrossing species, the three sets of SNPs were further split into HA sets (comprising markers heterozygous in the female parent and homozygous in the male parent), AH sets (homozygous in the female parent and heterozygous in the male parent) and HH sets (heterozygous in both parents), leading to a total of six F1 datasets^30^ and three F2 datasets (Supplementary Data 1). For the F2 population, information from the grandparents was used to rescore the progeny using the rules listed in Supplementary Table 2 to ensure that all markers were in the same linkage phase.

To assist with scaffold ordering and assessment of the quality of the assembly, 500 bp on either side of mapped SNP markers were excised from the assembly used for GBS read alignment and mapped to consecutive improved versions of the assembly using BLASTN. The sequences and location of the mapped F1 and F2 markers on the seashore paspalum version 3.0 assembly reported here as determined by the top BLASTN hit are provided in Supplementary Note 1. Discrepancies between marker orders in any two of the nine maps and the order and orientation of scaffolds in the pseudomolecules triggered manual examination and in some cases error correction.

### Gene family analysis in grasses

Protein sequences of the primary transcripts for seven species were retrieved from Phytozome: *Zea mays*, *Sorghum bicolor*, *Setaria italica*, *Paspalum vaginatum*, *Oropetium thomeaum*, *Brachypodium distachyon*, and *Oryza sativa*^143^. These sequences were used as inputs for orthoFinder^144,145^ to generate clusters of genes representing gene families. Family expansion and contraction were determined with CAFE (v5) using default settings^146^. Significantly expanded gene families in paspalum were defined as those with significantly different lambda value (*p* < 0.05) that showed increases in gene copy numbers in the lineage leading to paspalum as estimated using CAFE5^146^.

### RNA isolation, sequencing, and quantification

Root samples were homogenized by grinding to a fine powder in liquid nitrogen. Approximately 50 mg of homogenized root tissue per sample was mixed with 1 mL of TRIzol (Invitrogen® SKU: 15596018) reagents by robust vortexing and, incubated at room temperature (25 °C) for 10 min. The samples were mixed with 200 µL chloroform (TCI® SKU: C0819) and incubated for 15 min at room temperature until a clear separation of three layers was observed. The tubes containing the mixtures were centrifuged at 13,800 × *g* for 15 min to achieve phase separation. The top layer was transferred to a new set of tubes containing 400 µL isopropanol (TCI® SKU: I0163) and incubated on ice for at least 30 min. RNA precipitation was achieved by centrifugation at 13,800 × *g* for 15 minutes at 4 °C. Following the removal of the supernatants, the precipitates were washed with 75% ethanol (Decon Labs,Inc® SKU:2701) three times before being dissolved in 40 µL of 65 °C DEPC treated water (Invitrogen® SKU:AM9915G).

The quality of individual RNA samples was assessed using an Agilent 2100 Bioanalyzer. Samples with RNA Integrity Number (RIN) values >5 were used to isolate mRNA and construct RNA sequencing libraries using a TrueSeq v2 kit from Illumina^147^. Paired-end sequence data (2 × 75 bp) were generated using an Illumina NextSeq 500 platform. The overall quality of the RNAseq reads was assessed using FASTQC^148^ (Supplementary Fig. 5a). Demultiplexed reads were filtered and quality trimmed using Trimmomatic (v0.33) with the parameters “-phred33 LEADING:3 TRAILING:3 slidingwindow:4:15 MINLEN:36 ILLUMINACLIP:TruSeq3-PE.fa:2:30:10”^149^. Trimmed reads were mapped to the reference genomes of their respective species using STAR (v2.7)^150^ with two rounds of mapping; the first round of mapping was run with the parameters “–alignIntronMin 20 –alignIntronMax 20000 –outSAMtype None – outSJfilterReads Unique –outSJfilterCountUniqueMin 10 3 3 3 –outSJfilterCountTotalMin 10 3 3 3” and the second round of mapping was run after a new genome index was built based on the known and novel splicing sites recognized by the first round of mapping with parameters “–alignIntronMin 20 –alignIntronMax 20000 –limitBAMsortRAM 5000000000 –outSAMstrandField intronMotif –alignSJoverhangMin 20 –outSAMtype BAM SortedByCoordinate”. Maize reads were mapped to B73_RefGen_V4^122^. Sorghum reads were mapped to v3.1 of the BTx623 reference genome downloaded from Phytozome^123^. Paspalum reads were mapped to the paspalum genome assembly described and released as a part of this paper. A Transcripts Per Million (TPM) table was generated using Kallisto (v0.46.2)^151^. Syntenic orthologous genes across paspalum, maize and sorghum with a mean TPM value higher than 50 were log transformed prior to principal component analysis (Supplementary Fig. 5b). For each individual sequencing library, the read counts were determined using the software package HTSeq (version 0.9) with the parameter settings “-r pos -s no -t exon -i gene_id”, the overlap mode used was the default (“union”)^152^. Statistically significant DEGs were identified from the read count matrix generated by HTSeq using DESeq2 (v1.22.2)^153^ (Supplementary Fig. 5b). Genes were considered to be significantly differentially expressed when an absolute log_2_ fold change >1 and an adjusted *p* value lower than 0.05 were both observed. Total RNA of paspalum shoot was extracted using the same method and sequenced using the same library preparation protocol and sequencing platform as other samples described in this study, only genes with TPM higher than 5 and syntenically conserved were examined. The statistical significance of expression level changes was calculated using DESeq2^153^.

### Gene ontology enrichment analysis

Gene ontology (GO) enrichment analysis of the DEGs was performed using GOATOOLS^37^. To ensure consistency in cross species comparisons, the same population of syntenically conserved genes in maize, sorghum, and paspalum was used as the population set for enrichment analysis in each species. Similarly, to avoid bias introduced by the use of different GO term annotation pipelines, the same set of GO terms was assigned to each syntenic ortholog in each of the three species. These annotations were taken from the GO terms assigned to the maize copy of each conserved syntenic gene group by Maize-GAMER^154^. As the whole-genome duplication in maize introduced bias into the background gene set (genes retained as duplicate homeologous gene pairs are enriched in the annotations transcription factor, “responds to X” and protein complex subunit) only a single copy from maize1 subgenome of each maize gene pair was retained for both the background population set and the DEG defined set.

### Immunoblot detection of free ATG8 and ATG8-PE conjugate

Maize seedlings were grown under full-nutrient or N-deficient conditions for three weeks with or without the 30 µM validamycin A treatment described above. Root tissues were collected in a dark room solely illuminated by green light and ground to a fine powder in liquid nitrogen. The ground root tissues were homogenized in lysis buffer (50 mM Tris-HCl, pH 8.0, 150 mM NaCl, 1 mM phenylmethylsulfonyl fluoride, 10 mM iodoacetamide, and 1× complete protease inhibitor cocktail [Sigma Aldrich, St. Louis, MO, USA)]) and centrifuged at 2000 × *g*, 4 °C for 5 min. The extracted protein samples were quantified using a Bradford assay, and 25 µg protein was loaded onto a 15% SDS-PAGE (polyacrylamide gel electrophoresis) gel containing 6 M urea. Immunoblotting was performed with affinity-purified anti-At polyclonal ATG8 antibodies (1:1000 dilution)(Agrisera, Vännäs, Sweden; Catelog Number: AS14 2769). To confirm the ATG8-PE band and a more specific blot for the W22 experiment, the supernatant from the previous step was centrifuged at 100,000 × *g* at 4 °C for 1 h. A total of 200 µL supernatant was taken as soluble protein sample as one of the ATG8-PE negative control^81,155,156^. The pellet was then solubilized in 700 µL TNPI buffer containing 0.5% (v/v) Triton X-100. The dissolved pellet was then centrifuged at 16,000 × *g* for 10 min at 4 °C and 650 µL of the supernatant was taken as membrane protein. Concentrations of all the protein samples were confirmed by Bradford assay. The ATG8-PE (lipidation) band was confirmed by incubating protein samples at 37 °C for 1 h with *Streptomyces chromofuscus* phospholipase D (Thermo Fisher Scientific, Waltham, MA, USA; 525200-250U; 250 units mL^−1^ final concentration)^75^. Full gel picture with biomass markers used in Fig. 6 was shown in Supplementary Fig. 14f. Phospholipase D (250 U/tube) was stored in −80 °C and dissolved fresh by 200 µL dilution buffer containing 10 mM Tris-HCl, pH 8.0, 0.5% Triton X-100 and 5 mg/ml BSA to one tube to make a 5X solution.

### Reporting summary

Further information on research design is available in the Nature Portfolio Reporting Summary linked to this article.

## Supplementary information

Supplementary Information

Peer Review File

Description of Additional Supplementary Files

Supplementary Data 1

Supplementary Data 2

Supplementary Data 3

Supplementary Data 4

Reporting Summary

## Data Availability

RNAseq data for root tissue of paspalum, maize and sorghum under three nutrient conditions are deposited in NCBI under BioProject PRJNA746310. RNAseq data for root tissue of maize seedlings grown under three nutrient conditions with or without validamycin A treatment are also available at NCBI under BioProject PRJNA746310. RNAseq data for paspalum shoots/rhizome are deposited in NCBI under SRA accessions SRR10230104, SRR10230108, SRR10230122, and SRR10230130. RNAseq data for *atg12-1* mutant and wild-type W22 maize plants are deposited in NCBI under BioProject PRJNA449498. The genome assembly and sequence data of *Paspalum vaginatum* are deposited in NCBI under BioProject PRJNA234783 and genome accession in Genbank under JAPDNB000000000. The genome sequence and annotation are also accessible via Phytozome v13 [https://phytozome-next.jgi.doe.gov/info/Pvaginatum_v3_1]. Source data are provided with this paper.

## Source data

Source Data

## Code availability

All the scripts and input data used for generating main figures and codes used for generating Supplementary Figures in this study have been deposited in Zenodo [https://zenodo.org/badge/latestdoi/505987272], which can also be accessed from Github [https://github.com/xiaoguanghuan123/paspalumGenome]^157^
