# Supplementary material for: Genome of Paspalum vaginatum and the role of trehalose mediated autophagy in increasing maize biomass
Source: Nat Commun. 2022 Dec 13;13:7731. doi: 10.1038/s41467-022-35507-8 (PMC9747981; doi:10.1038/s41467-022-35507-8)
Supplement: Supplementary file 1 — Supplementary Information [file 41467_2022_35507_MOESM1_ESM.pdf]

Genome of *Paspalum vaginatum* and the role of trehalose mediated autophagy in increasing maize biomass

Sun *et al.*

Supplementary Table 1. Final summary assembly statistics for chromosome scale assembly.

Scaffold total	1902
Contig total	2211
Scaffold sequence total	646.9 Mb
Contig sequence total	643.8 Mb (0.5% gap)
Scaffold L/N50	7 / 44.5 Mb
Contig L/N50	111/ 1.5 Mb
Number of scaffolds >50 Kb	1111
% main genome in scaffolds >50 Kb	95.5 %

Supplementary Table 2. Rescoring of progeny based on allele information from grandparents.

Grandparent 1	Grandparent 2	Parents	Rule (to be applied to the F2 progeny)	Marker suffix
A	H	AH or HA	Keep original scores	-
H	A	AH or HA	Change ‘A’ to ‘H’, and ‘H’ to ‘A’	R
H	H	AH or HA	Duplicate marker; keep original scores in one copy (marker suffix ‘u’), change ‘A’ to ‘H’ and ‘H’ to ‘A’ in second copy (marker suffix ‘ur’)	u / ur
A	B	HH	Keep original scores	-
A	H	HH	Keep original scores	-
H	B	HH	Keep original scores	-
B	A	HH	Change ‘A’ to ‘B’, and ‘B’ to ‘A’	r
H	A	HH	Change ‘A’ to ‘B’, and ‘B’ to ‘A’	r
B	H	HH	Change ‘A’ to ‘B’, and ‘B’ to ‘A’	r
H	H	HH	Duplicate marker; keep original scores in one copy (marker suffix ‘u’), change ‘H’ to ‘-’ and both ‘A’ and ‘B’ to ‘H’ in second copy (marker suffix ‘uD’)	u / uD

Supplementary Table 3. Genomic libraries included in the *Paspalum vaginatum* genome assembly and their respective assembled sequence coverage levels in the final release.

Library	Sequencing platform	Average read/Insert size	Read number	Assembled sequence coverage (x)
USGX	Illumina	400	266,209,446	102.4
	PacBio	9,523*	5,012,142	74.30
Total		N/A	271,221,588	176.70

Supplementary Table 4. PacBio library statistics for the libraries included in the *Paspalum vaginatum* genome assembly and their respective assembled sequence coverage levels.

Cutoff	Number of reads	Base pairs	Average read length	Coverage
0	5,021,142	52,011,227,922	9,523	74.30x
1,000	4,598,012	51,803,193,177	10,358	74.00x
2,000	4,284,360	51,337,470,200	10,963	73.34x
3,000	4,011,480	50,660,511,262	11,490	72.38x
4,000	3,772,204	49,824,505,385	11,967	71.19x
5,000	3,545,694	48,806,129,176	12,435	69.72x
6,000	3,326,274	47,599,675,919	12,908	68.00x
7,000	3,100,356	46,130,347,947	13,419	65.91x
8,000	2,871,056	44,410,763,109	13,968	63.44x
9,000	2,637,734	42,426,702,674	14,563	60.61x
10,000	2,390,849	40,080,198,809	15,239	57.26x
11,000	2,132,551	37,367,664,499	16,009	53.38x
12,000	1,878,010	34,441,849,335	16,844	49.20x
13,000	1,642,413	31,498,764,753	17,705	45.01x
14,000	1,429,052	28,620,342,486	18,575	40.89x
15,000	1,238,057	25,852,785,076	19,449	36.93x
16,000	1,067,747	23,214,688,325	20,327	33.16x
17,000	916,859	20,726,586,386	21,209	29.61x
18,000	784,038	18,403,847,127	22,088	26.29x
19,000	666,673	16,233,960,809	22,979	23.19x

Supplementary Table 5. Summary statistics of the initial output of the QUIVER polished MECAT assembly. The table shows total contigs and total assembled basepairs for each set of scaffolds greater than the size listed in the left hand column.

Minimum Scaffold Length	Number of Scaffolds	Number of Contigs	Scaffold Size	Basepairs	% Non-gap Basepairs
5 Mb	4	4	30,869,566	30,869,566	100.00%
2.5 Mb	54	54	202,593,315	202,593,315	100.00%
1 Mb	165	165	377,268,994	377,268,994	100.00%
500 Kb	296	296	472,159,561	472,159,561	100.00%
250 Kb	444	444	524,257,651	524,257,651	100.00%
100 Kb	1,009	1,009	604,765,225	604,765,225	100.00%
50 Kb	3,282	3,282	755,766,926	755,766,926	100.00%
25 Kb	5,279	5,279	836,810,963	836,810,963	100.00%
10 Kb	5,358	5,358	838,436,451	838,436,451	100.00%
5 Kb	5,358	5,358	838,436,451	838,436,451	100.00%
2.5 Kb	5,358	5,358	838,436,451	838,436,451	100.00%
1 Kb	5,358	5,358	838,436,451	838,436,451	100.00%
0 bp	5,358	5,358	838,436,451	838,436,451	100.00%

Supplementary Table 6. Protocol for making Hoagland Full nutrient solution used in this study.

Hoagland full nutrient solution (1L)					
Concentration	Chemical	wt(g)/L	For 250 mL Stock	mL/L for working stock	Final concentration
2 M	KNO_3	202.2	50.55	2.5	5 mM
2 M	$Ca(NO_3)_2.4H_2O$	472.3	118.075	2.5	5 mM
1 M	KH_2PO_4(PH=6)	136.09	34.0225	1	1 mM
2 M	$MgSO_4 .7H_2O$	492.94	123.3235	1	2 mM
0.05 M	Fe-EDTA*	2.8	0.7	1	50 uM
	Micro Nutrients			1	

*3.72 g DETA in 70 mL ddiH_2O mixed with 2.78 g $FeSO_4.7H_2O$ in 70 mL ddiH_2O and then volume up to 200 mL

Supplementary Table 7. Protocol for making Hoagland -N nutrient solution used in this study

Hoagland -nitrogen nutrient solution (1L)					
Concentration	Chemical	wt(g)/L	For 250 mL stock	mL/L for working stock	Final concentration
2 M	K_2SO_4	87	21.75	10	5 mM
2 M	$CaCl_2.2H_2O$	294.02	73.505	2.5	5 mM
1 M	KH_2PO_4(PH=6)	136.09	34.0225	1	1 mM
2 M	$MgSO_4.7H_2O$	492.94	123.3235	1	2 mM
0.05 M	Fe-EDTA*	2.8	0.7	1	0.1mM
	Micro Nutrients			1	

*3.72 g DETA in 70 mL ddiH_2O mixed with 2.78 g $FeSO_4.7H_2O$ in 70 mL ddiH_2O and then volume up to 200 mL

Supplementary Table 8. Protocol for making Hoagland -P nutrient solution used in this study.

Hoagland -phosphorus nutrient solution (1L)					
Concentration	Chemical	wt(g)/L	For 250 mL stock	mL/L for working stock	Final concentration
2 M	KNO_3	202.2	50.55	2.5	5 mM
2 M	$Ca(NO_3)_2.4H_2O$	472.3	118.075	2.5	5 mM
1 M	K_2SO_4	87	21.75	10	10 mM
2 M	$MgSO_4$ $.7H_2O$	492.94	123.3235	1	2 mM
0.05 M	Fe-EDTA*	2.8	0.7	1	0.1mM
	Micro Nutrients			1	

*3.72 g DETA in 70 mL ddiH_2O mixed with 2.78 g $FeSO_4.7H_2O$ in 70 mL ddiH_2O and then volume up to 200 mL

Supplementary Table 9. Protocol for making micro nutrients used for Full, -N and -P nutrient solution used in this study.

Micro nutrients (1L)		
Final concentration	Chemical	wt(g)/L
0.046 M	H_3BO_3	2.86
0.009 M	$MnCl_2.4H_2O$	1.81
7.5X10-4 M	$ZnSO_4.7H_2O$	0.22
3.2X10-4 M	$CuSO_4$	0.08
1.11X10-4 M	$H_2MoO_4.4H_2O$	0.02

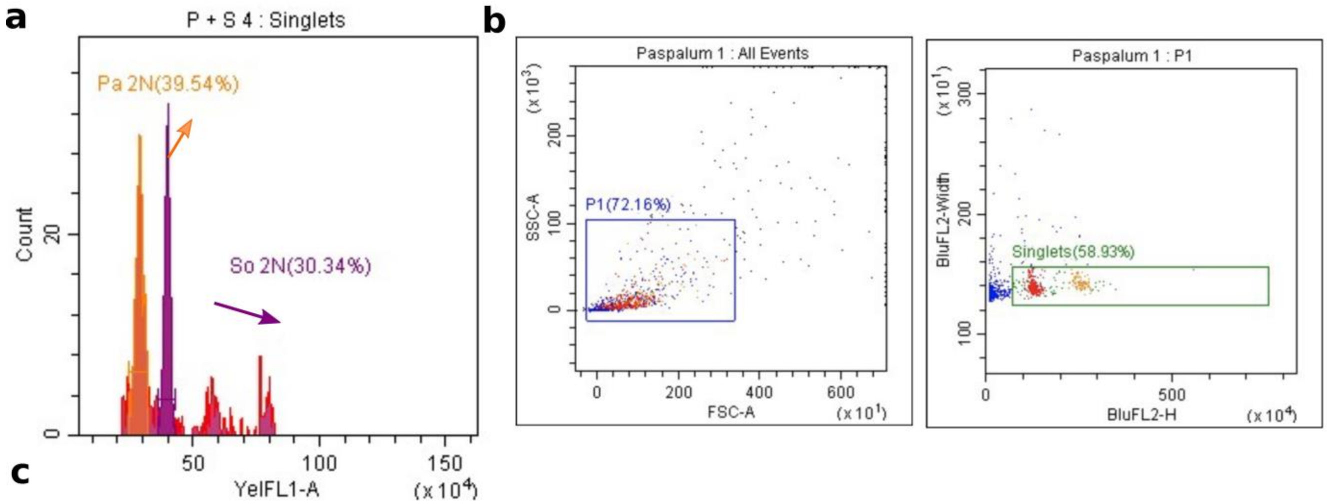

c

Population	Events	% Total	% Parent	Median YelFL1-A
All Events	2290	100.00%	100.00%	35328.1
P1	1463	63.89%	63.89%	49256.0
Singlets	435	19.00%	29.73%	372482.7
Pa 2N	172	7.51%	39.54%	285848.0
So 2N	132	5.76%	30.34%	396332.3

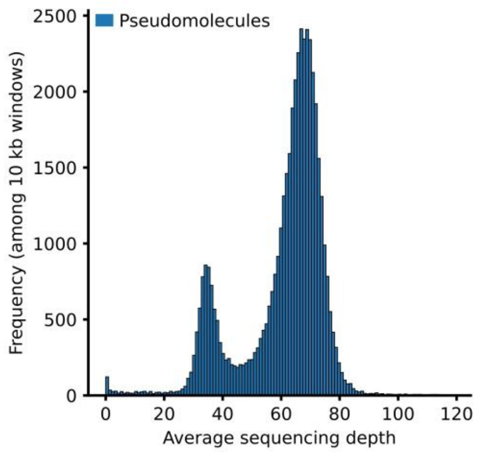

Supplementary Figure 1. Estimating the genome size of the paspalum accession employed for genome sequencing. a Estimation of genome size using flow cytometry. The x axis indicates yellow fluorescence intensity (YelFL1-A), which is linearly correlated with the genome size. *Sorghum bicolor* BTX623 nuclei (So 2N) were used as an internal control. **b** The FSC/SSC gate was drawn around the primary particle population shown on the plot, while eliminating much larger particles and smaller debris. Colors are defined in table shown in panel **c**. **c** Statistics of the flow cytometry results for one representative sample. Based on the median yellow fluorescence intensity, the ratio of genome size of *Paspalum vaginatum* (Pa) to *Sorghum bicolor*(So) is 285848:396332. The *Sorghum bicolor* genome is 818 Mbp in size; therefore, the estimated genome size of *Paspalum vaginatum* is 590 Mbp. **d** Genome wide read coverage of Illumina sequencing reads mapped to the current paspalum genome assembly (kb = kilobases). Source data are provided as a Source Data file.

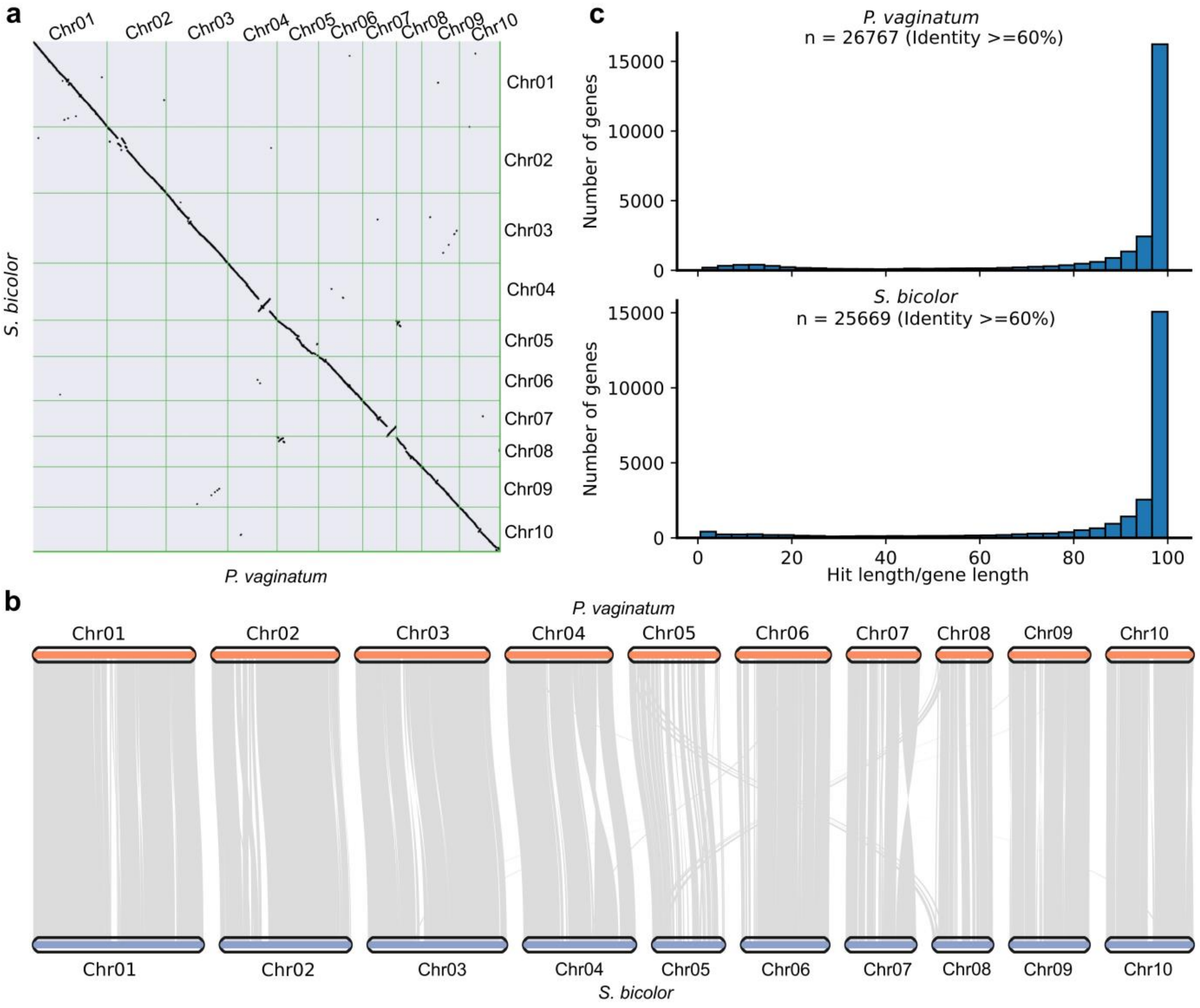

Supplementary Figure 2. Synteny between paspalum and sorghum genome. a,b Syntenic regions conserved between the paspalum genome (orange) and sorghum genome (blue). **c** Length of homologs with identity >60% over the annotated length of proteins annotated in paspalum genome when BLAST paspalum proteome against sorghum proteome (upper) using BLAST (v2.5.0+) and reversely, the length of homologs with identity >60% over the annotated length of proteins annotated in sorghum genome when BLAST sorghum proteome against paspalum proteome (lower). Source data are provided as a Source Data file

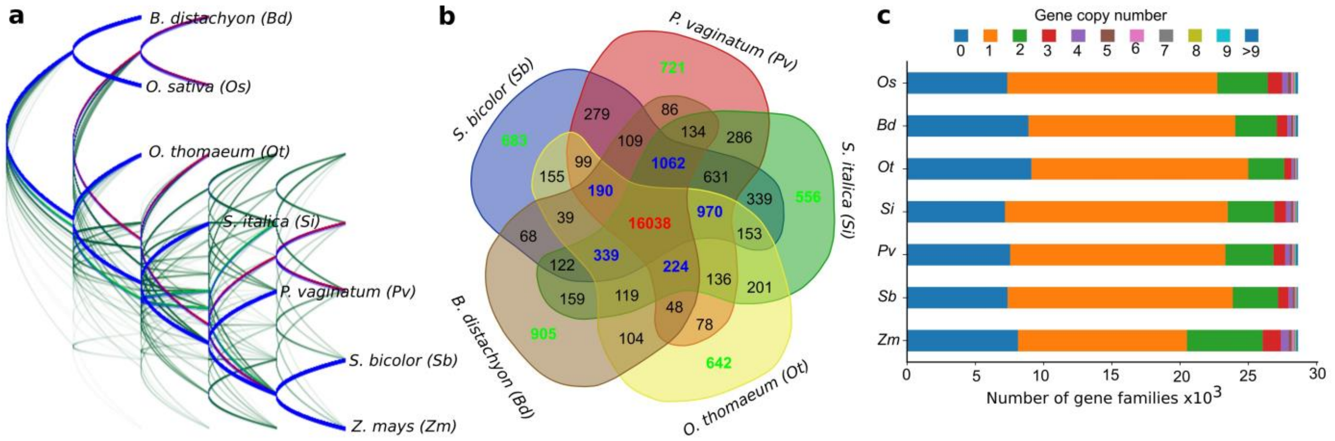

Supplementary Figure 3. Gene families identified among seven grass species. a DensiTree(v2.2.6) drawn from phylogenies constructed based on selected individual single-copy sytenic orthologous gene pairs across species: paspalum (*Paspalum vaginatum*), maize (*Zea mays*), sorghum (*Sorghum bicolor*), foxtail millet (*Setaria italica*), *Oropetium* (*Oropetium thomaeum*), *Brachypodium* (*Brachypodium distachyon*), and rice (*Oryza sativa*). The consensus tree drawn in blue was supported by 4,265 (73%) of the individual gene trees and the second most common topology drawn in purple was supported by 762 individual gene trees (13%). **b** Comparison of shared and species-specific gene families among the five grass species. Green numbers indicate species-specific gene families. Blue numbers indicate gene families shared by all but one of the five species compared, while numbers in red indicate the number of gene families shared across all five species. Gene families shared by either two or three of the five species are shown in black. Maize and rice which do not have a unique most recent common ancestor (MRCA) with paspalum (the MRCA of maize and paspalum is the MRCA of sorghum and pasaplum, and the MRCA of rice and pasplaum is the MRCA of *Brachypodium* and paspalum), were omitted to simplify visualization. **c** Distribution of copy numbers for gene families in each of the seven species shown in panel **a**. Source data are provided as a Source Data file.

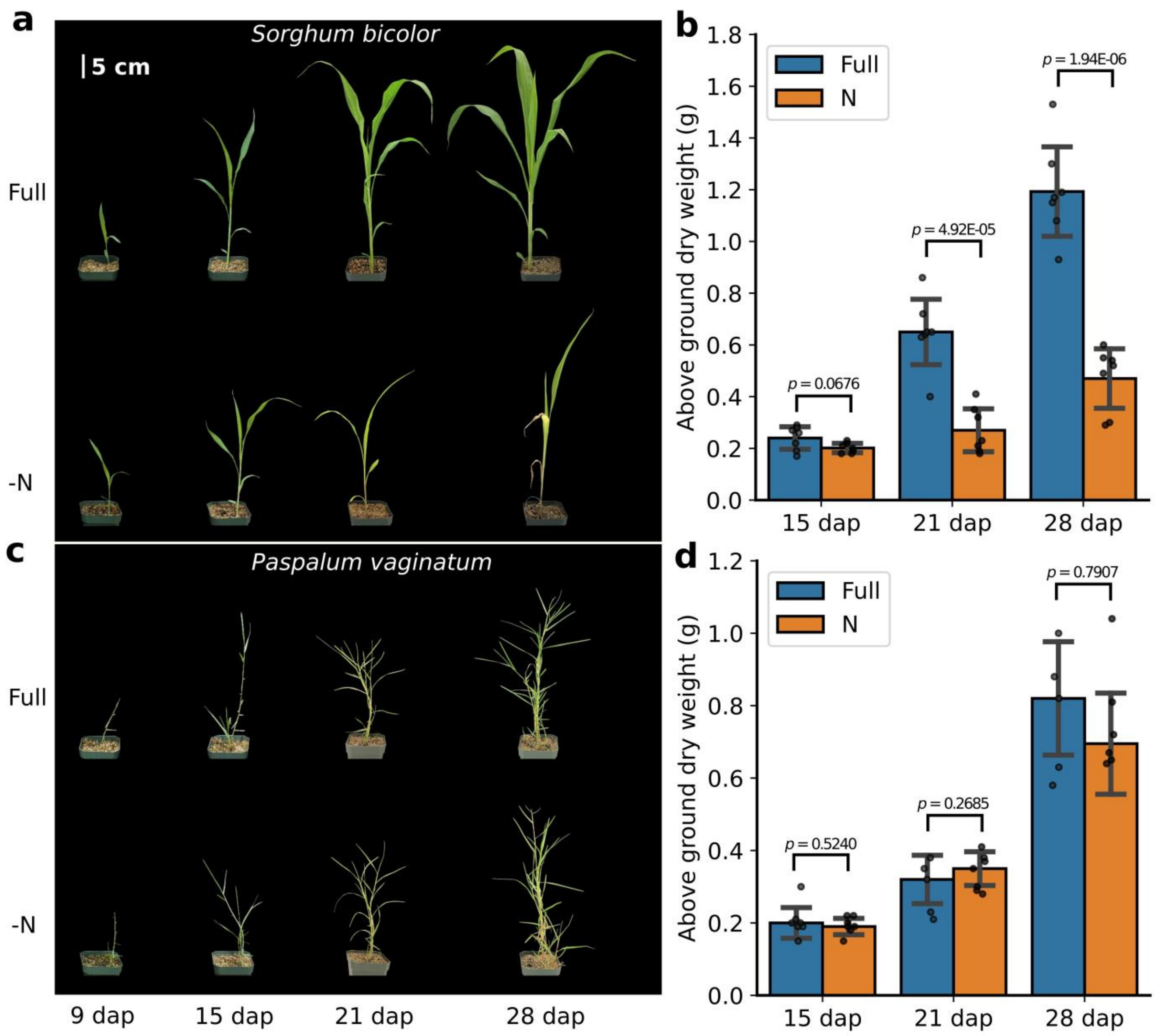

Supplementary Figure 4. Time-course biomass accumulation by paspalum and sorghum under Full and -N conditions. a Representative images of sorghum seedlings at 9, 15, 21 and 28 days after planting (dap) grown under Full or -N conditions. **b** Above ground dry biomass accumulation of sorghum at 9, 15, 21 and 28 days after planting (dap) grown under Full or -N conditions. **c** Representative images of paspalum seedlings at 9, 15, 21 and 28 days after planting (dap) grown under Full or -N conditions. **d** Above ground dry biomass accumulation of paspalum at 9, 15, 21 and 28 days after planting (dap) grown under Full or -N conditions. For **b-d**: significance of comparisons were determined by Student's t-test; two tailed. Bar heights for panels **b-d** are means of n = 5 independently measured plants from a single trial. Error bars for panels **b-d** indicate standard deviations centered on mean values. Source data are provided as a Source Data file.

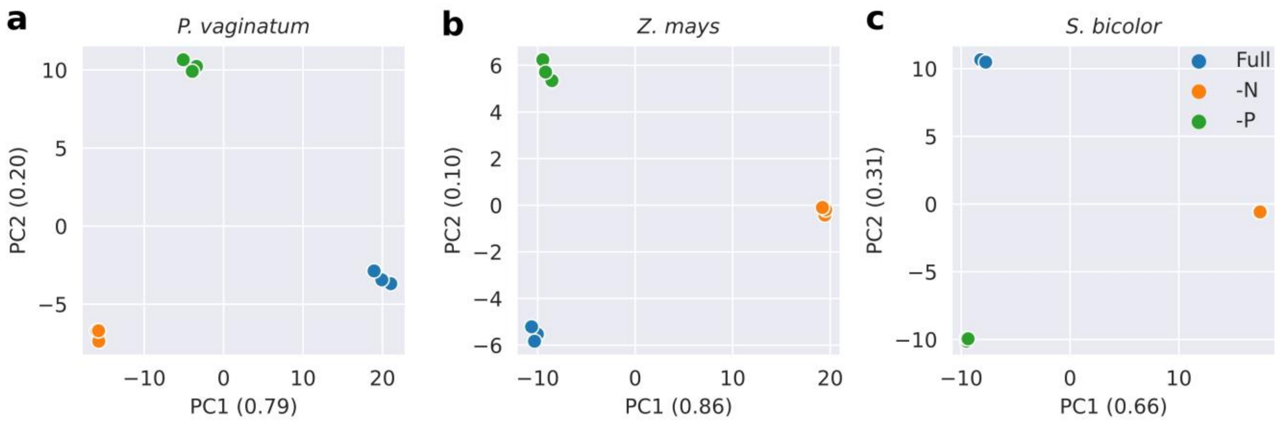

Supplementary Figure 5. Principal component analysis of biological replicates of root transcriptomes under three experimental nutrient conditions. a Principal component analysis based on log transformed expression of syntenic genes in maize (*Zea mays*). **b** Principal component analysis based on log transformed expression of syntenic genes in sorghum (*Sorghum bicolor*). **c** Principal component analysis based on log transformed expression of syntenic genes in paspalum (*Paspalum vaginatum*). For panels **a-c**: Nutrient conditions are color coded. PC, principal component. The PCs were calculated from gene expression values in each RNAseq library generated from each of three biological replicates of each species under each condition. Source data are provided as a Source Data file.

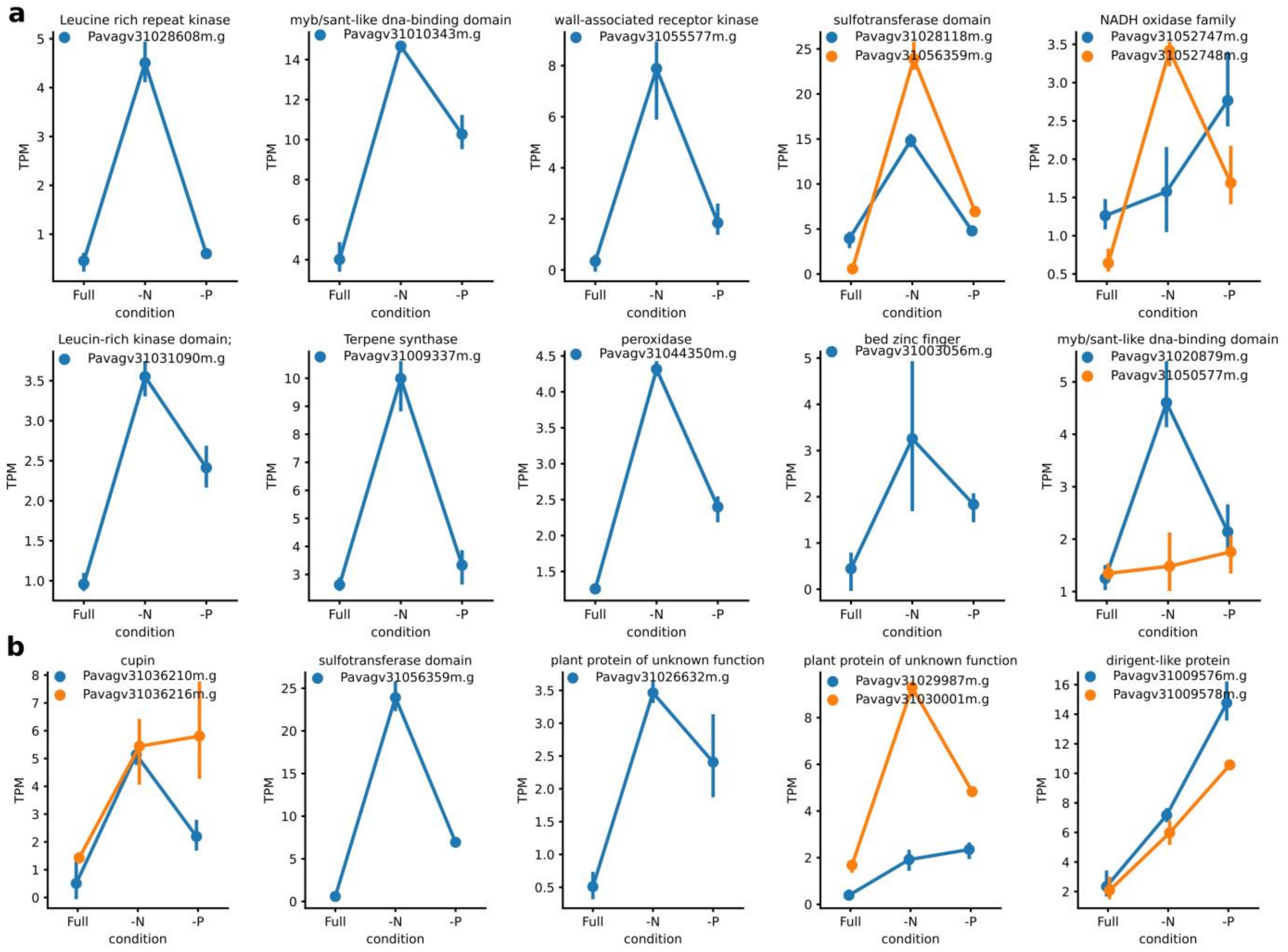

Supplementary Figure 6. Members of gene families that are transcriptionally responsive to nutrient-deficit conditions. a Members of paspalum-specific expanded gene families that are transcriptionally responsive to nitrogen deficiency. **b** Members of paspalum-specific expanded gene families that are transcriptionally responsive to phosphorus deficiency. In panels **a** and **b**, points indicate mean values and error bars indicate the standard deviation across n=3 biological replicates, each an RNA-seq library built from RNA extracted from a different pool of plants. Source data are provided as a Source Data file.

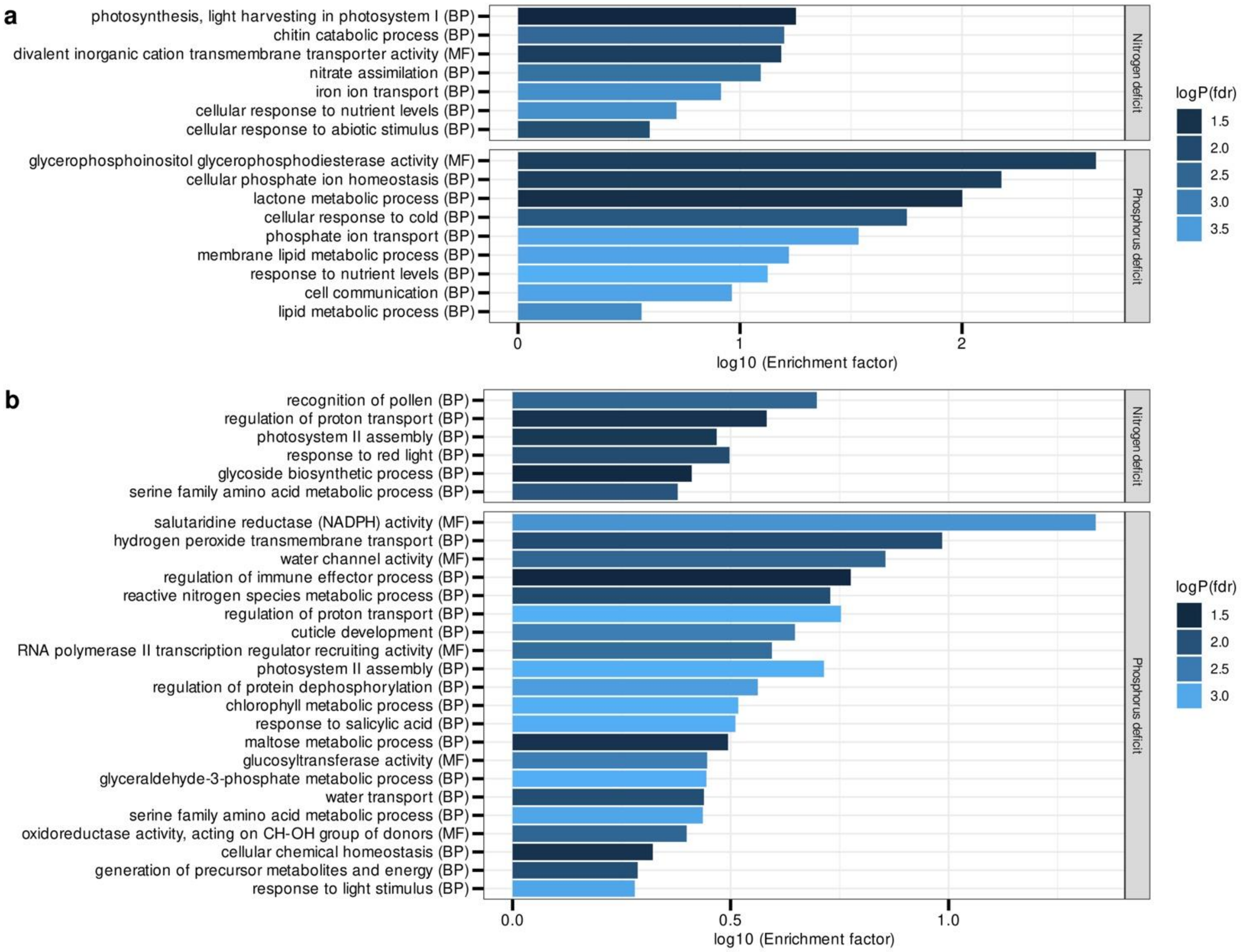

Supplementary Figure 7. Gene ontology (GO) analysis of differentially expressed syntenic orthologous genes across the three species and in papspalum alone. a Significantly enriched GO terms (false discovery rate (FDR) ≤ 0.05) for 220 and 37 syntenic orthologous genes that were differentially expressed in all the three species in response to N-deficit and P-deficit conditions, respectively. Bars indicate the log-transformed enrichment factor (number of genes associated with the overrepresented GO terms in the study gene set over the number of genes associated with the GO term in the background gene set) for enriched GO terms. Negative log-transformed multi-test corrected p values were color coded. **b** Significantly enriched GO terms (false discovery rate (FDR) ≤ 0.05) in 825 and 650 syntenic orthologous genes that were differentially expressed only in paspalum in response to N-deficit and P-deficit conditions, respectively. Bars indicate the log-transformed enrichment factor (number of genes associated with the overrepresented GO terms in the study gene set over the number of genes associated with the GO term in the background gene set) for enriched GO terms. Negative log-transformed multi-test corrected p values were color coded. Source data are provided as a Source Data file.

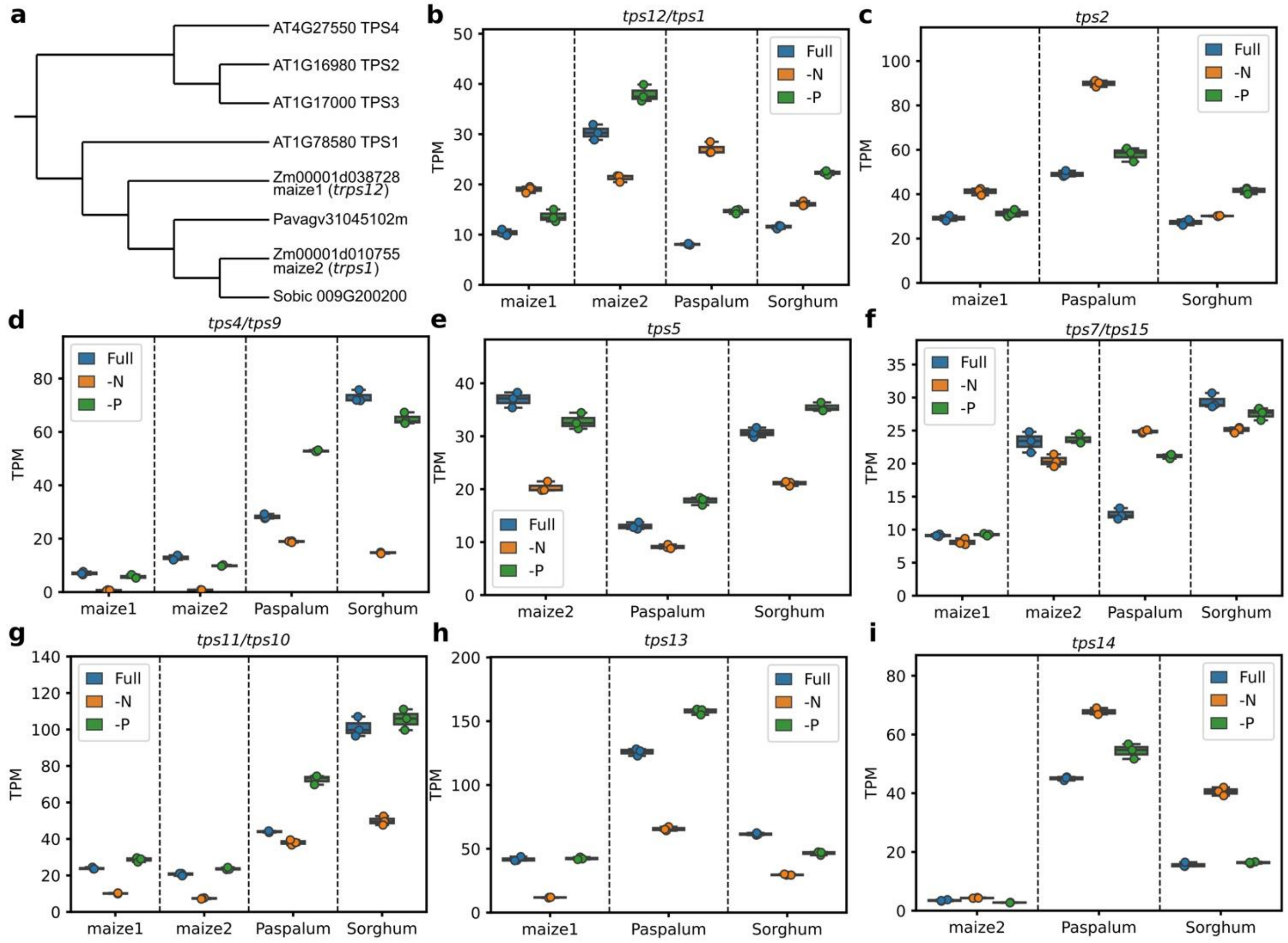

Supplementary Figure 8. Expression patterns of genes encoding trehalose-6-phosphate synthase in response to nutrient stress across three tested plant species. a Phylogeny of orthologs of Arabidopsis trehalose-6-phosphate synthase 1 (*TPS1*) genes in the three species. A full phylogeny including all annotated TPP homologues were included in source data file. **b** Expression pattern of the trehalose-6-phosphate synthase genes in the three species under nutrient-optimal (Full), nitrogen-deficit (–N), and phosphorus-deficit (–P) conditions. "maize1" and "maize2" indicate the two subgenomes that formed in maize after the recent whole-genome duplication event 12–16 million years ago. **c-i** Expression patterns of other syntenic genes annotated as encoding trehalose-6-phosphate synthase that did not cluster with Arabidopsis homologs in the three species under nutrient-optimal (Full), nitrogen-deficit (–N), and phosphorus-deficit (–P) conditions. For panels **b-i**, TPM of each gene was calculated from RNAseq libraries for three biological replicates for each species under each condition by Kallisto (v0.46.2). All box plots shown in panels **b-i** are defined as in the Fig. 1 legend. For panels **b-i**, n=3 biological replicates, each an RNA-seq library built from RNA extracted from a different pool of plants. Source data are provided as a Source Data file.

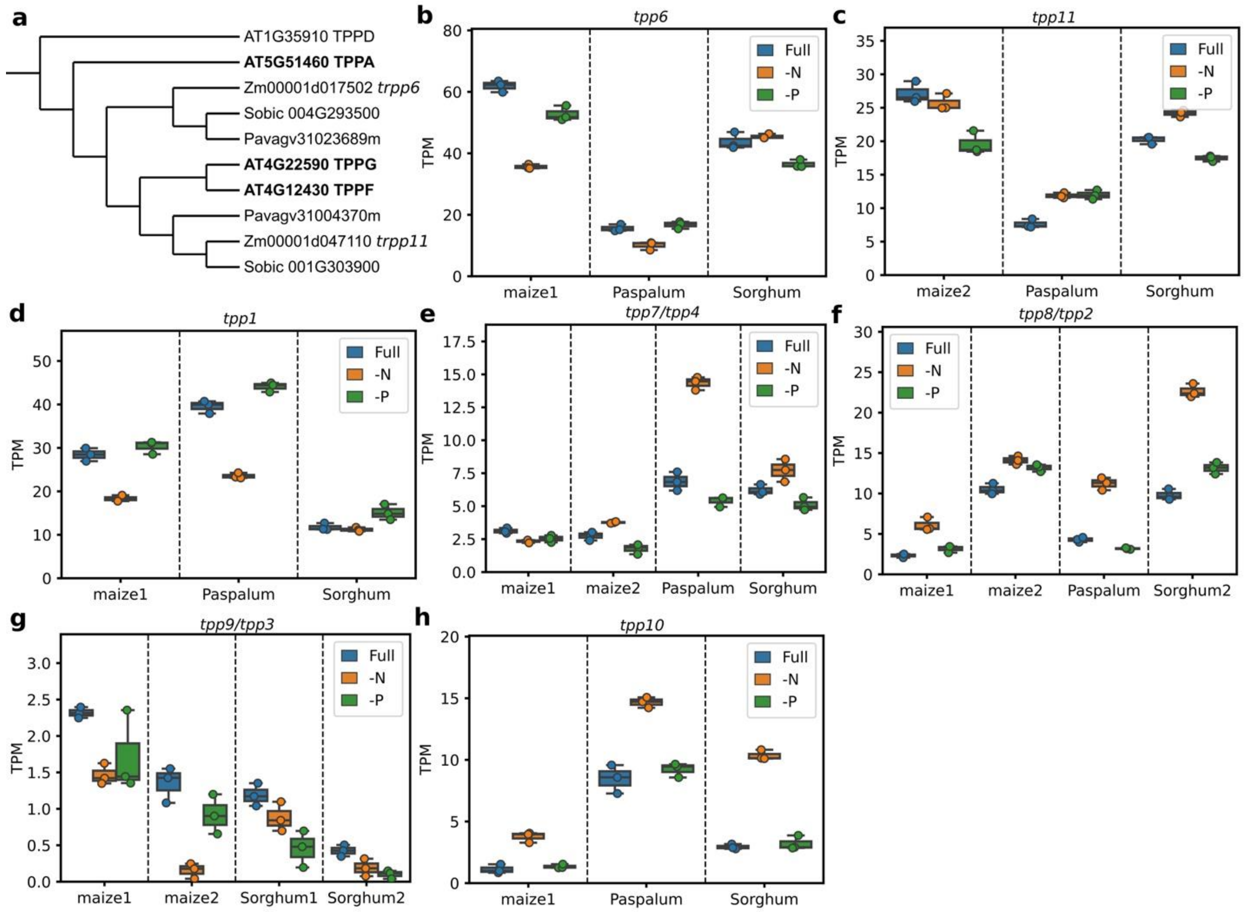

Supplementary Figure 9. Expression patterns of genes encoding trehalose-6-phosphate phosphatase enzymes in response to nutrient stress across three tested species. a Phylogeny of orthologs of characterized Arabidopsis trehalose-6-phosphate phosphatase (*TPP*) genes in the three species. **b,c** Expression patterns of the trehalose-6-phosphate phosphatase genes (*tpp6* and *tpp11*) that clustered with their Arabidopsis homologous (*tppa*, *tppg*, *tppf*) under nutrient-optimal (Full), nitrogen-deficit (–N), and phosphorus-deficit (-P) conditions. **d-h** Expression patterns of other syntenic genes annotated as trehalose-6-phosphate phosphatase that did not cluster with Arabidopsis homologs in the three species grown under nutrient-optimal (Full), nitrogen-deficit (-N), and phosphorus-deficit (-P) conditions. For panels **b** to **i,** "maize1" and "maize2" indicate the two subgenomes that formed in maize after the recent whole-genome duplication event 12–16 million years ago. All box plots shown in panels **b-h** are defined as in the Fig. 1 legend. For panels **b-h**, n=3 biological replicates, each an RNA-seq library built from RNA extracted from a different pool of plants. Source data are provided as a Source Data file.

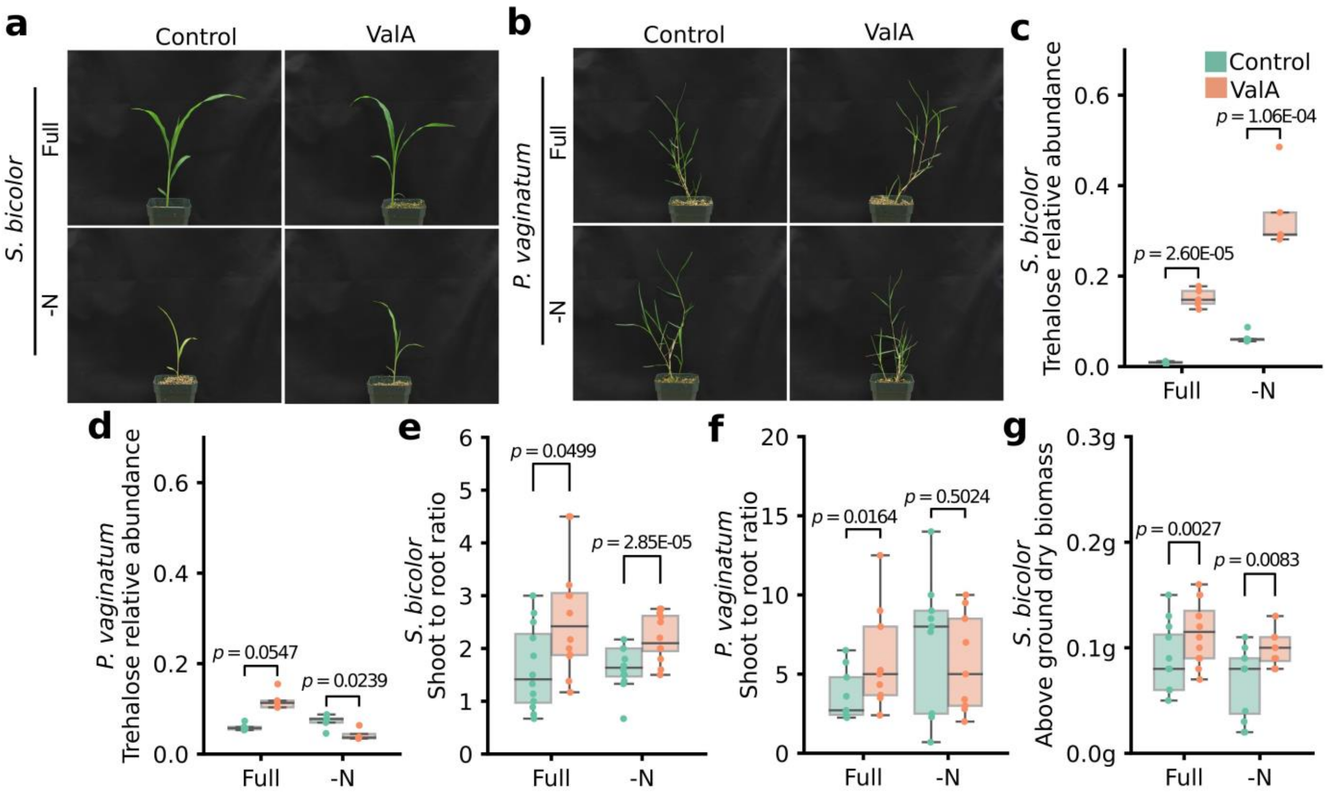

Supplementary Figure 10. ValA treatment alters biomass accumulation and nutrient reallocation to sorghum (*Sorghum bicolor*) grown under nutrient-deficient conditions. a Representative images of sorghum seedlings grown under nutrient optimal and N-deficit conditions with or without validamycin A (ValA) treatment. Images were taken 21 days after planting. For the ValA treatment, a 30µM solution was added at 6 PM on the day that the plants were watered with the indicated nutrient solutions. **b** Representative images of paspalum seedlings at 3 weeks after planting grown under nutrient optimal (Full) and nitrogen-deficient (-N) conditions with (ValA) or without (Control) validamycin A treatment. **c-d** Changes in observed trehalose abundance in five samples collected from three independent trials – normalized to an internal reference (ribitol) – in response to validamycin A and/or nutrient conditions in sorghum root tissues (**c**) and paspalum root tissues (**d**).**e-f** Shoot-to-root ratio calculated from the dry weight of above-ground tissues and roots of the same sorghum (**e**) and paspalum (**f**) seedlings. **g** Dry weight of the above-ground tissue of sorghum seedlings grown under nutrient-optimal and nitrogen-deficit conditions harvested at 3 weeks after planting. Plant tissues were freeze-dried for 48 hours after harvesting. For panels **c-g**: significance of comparisons were determined by two-sided Wilcoxon signed-rank test. All box plots shown in panels **c-g** are defined as in the Fig. 1 legend. Source data are provided as a Source Data file.

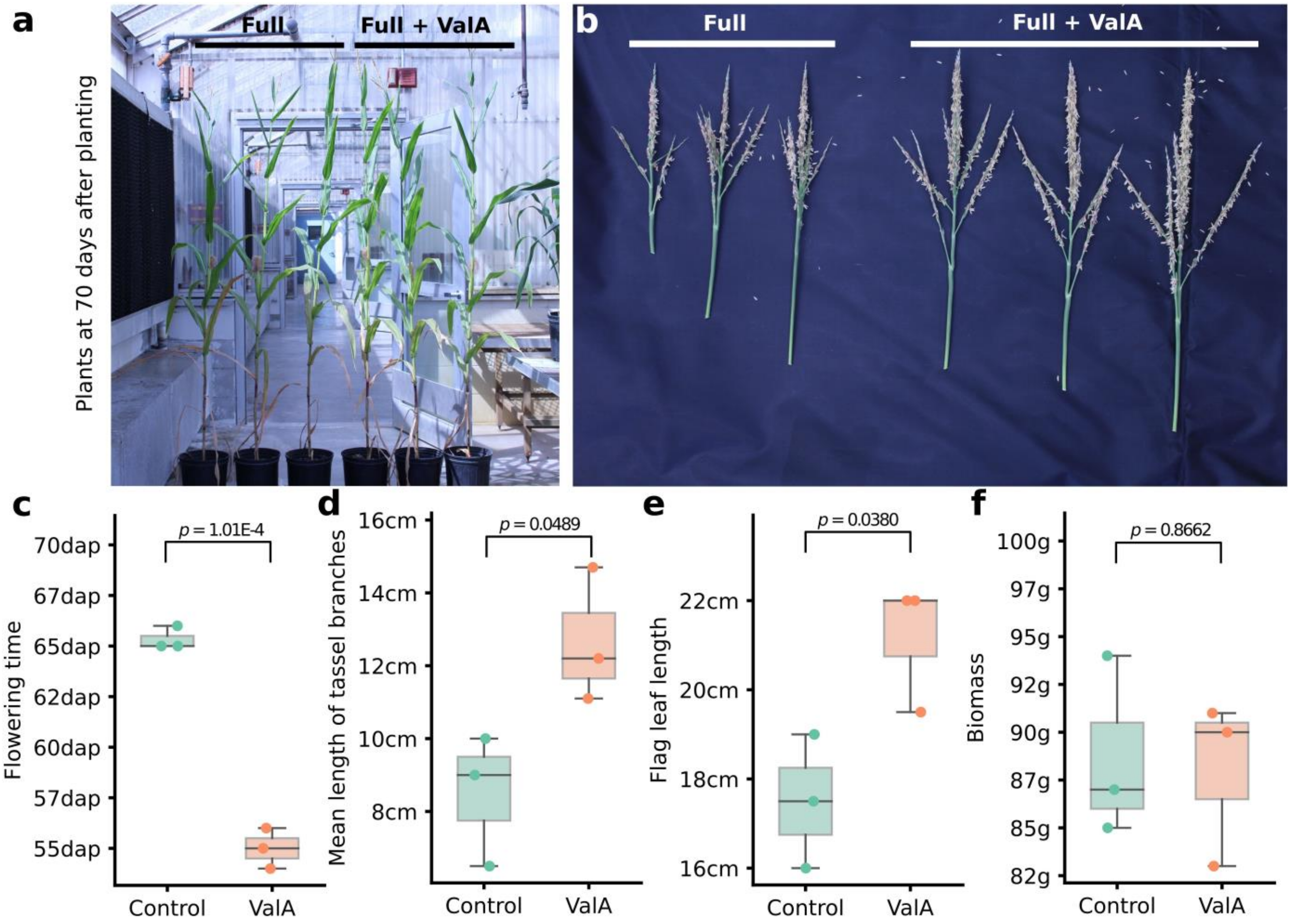

Supplementary Figure 11. Validamycin A treatment improves agronomic traits in adult maize plants. a,b Images of whole plants (**a**) and tassels (**b**) taken 70 days after planting. The left three plants were grown under full-nutrient conditions and the right three were grown under full-nutrient conditions with a weekly 30μM ValA treatment. **c-f** Flowering time (**c**), mean length of tassel branches (**d**), flag leaf length (**e**) and above ground dry biomass (**f**) of plants with or without ValA treatment. P values were determined by Student's t-test; two tailed; n=3 plants). Data were collected from a single trial with three replicates. All box plots shown in this figure are defined as in the Fig. 1 legend. Source data are provided as a Source Data file.

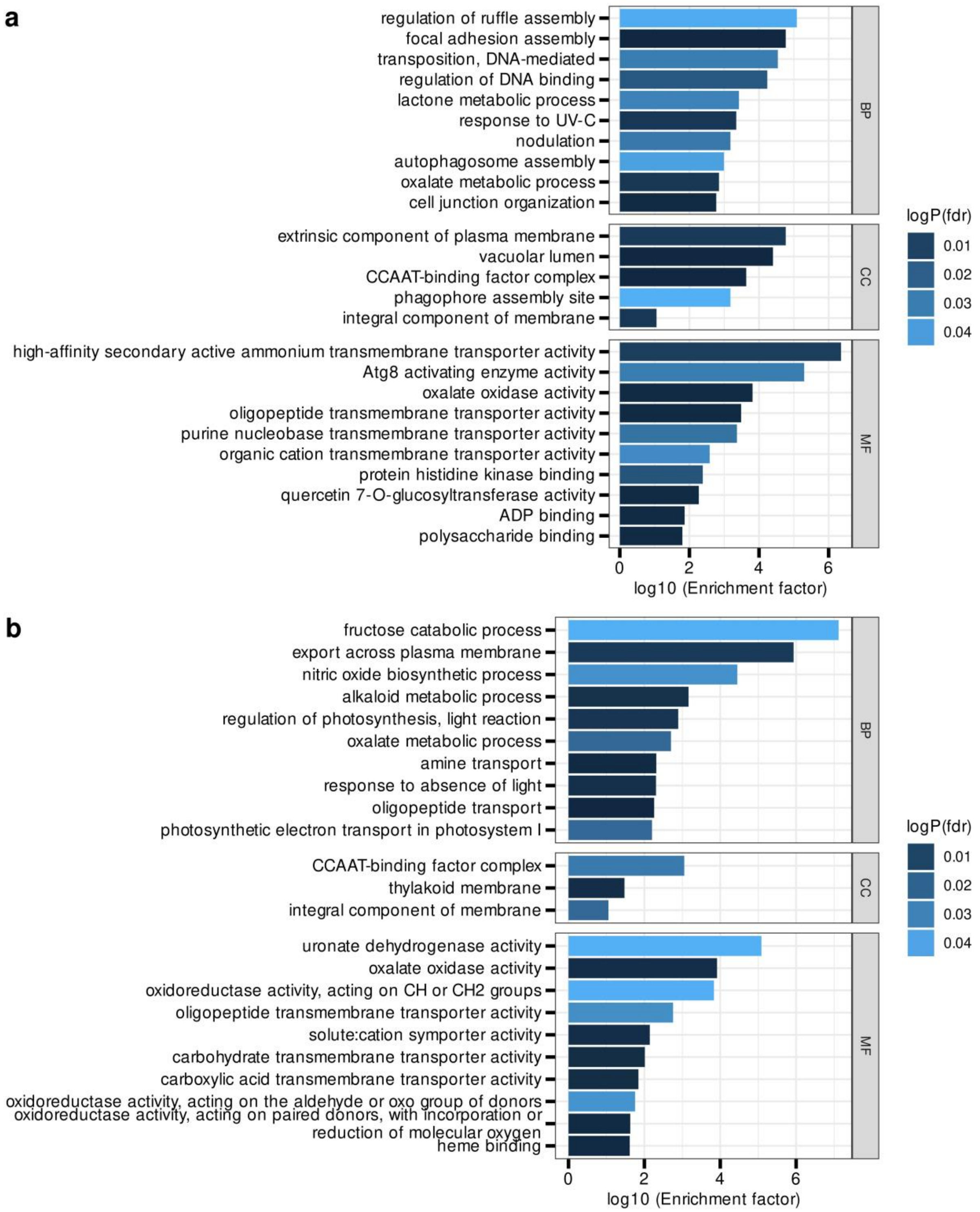

Supplementary Figure 12. GO enrichment of genes that were upregulated due to validamycin A treatment in maize seedlings grown under Full and -N conditions. **a** Top 10 GO terms ranked by enrichment factor among the up-regulated genes in seedlings grown under Full conditions with validamycin A treatment. **b** Top 10 GO terms in each category ranked by enrichment factor among the up-regulated genes in seedlings grown under -N conditions with validamycin A treatment.BP: Biological process; CC: Celullar compoment; MF: Molecular function; p_fdr_BH: p values calulated initially using Fisher’s exact test and controlling false discovery using the Benjamini Hochberg Procedure. Source data are provided as a Source Data file.

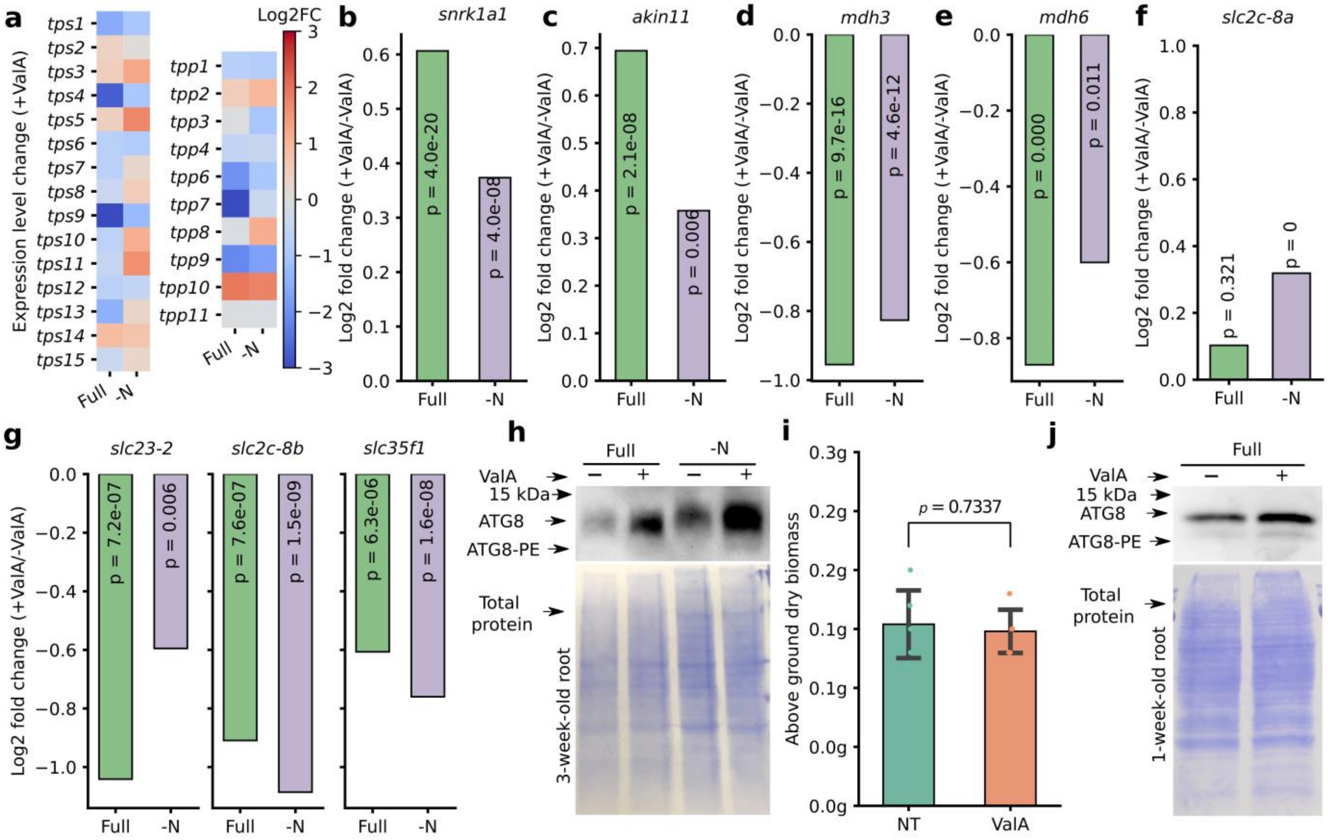

Supplementary Figure 13. Transcriptional responses of SNRK1 target genes to Validamycin A treatment. a Expression level fold changes of the genes encoding trehalose-6-phosphate synthases (TPS) and trehalose-6-phosphate phosphatases (TPP) relative to the seedlings treated with validamycin A under Full and -N conditions. **b,c** Change in the expression of the SNRK1 alpha subunit encoding maize genes *snrk1a1* (**b**) and *akin11* (**c**) in roots from three week old ValA treated maize seedlings in response to ValA treatment. **d,e** Change in the expression of SNRK1 repressed genes encoding malate dehydrogenase 3 (*mdh3*) (**d**) and malate dehydrogenase 6 (*mdh6*) (**e**) in roots from three week old ValA treated maize seedlings in response to ValA treatment. **f,g** One of the four genes encoding non-chloroplast Solute Carrier family glucose transporters SLC2C-8A (Zm00001d009603) is slightly upregulated (**f**) in response to ValA treatment while all other three SLC23-2 (Zm00001d012693), SLC2C-8 (Zm00001d029254) and SLC35F1 (Zm00001d038299) were significant down-regulated under Full and -N conditions with ValA treatment (**g**). All expression fold changes and corrected p values (*fdr* ≤ 0.05) shown are values calculated by DESeq2(v1.22.2) across three biological replicates per condition. **h** A biological replicate of the immunoblot measuring the abundance of both free ATG8 (upper band) and the ATG8-PE conjugate (lower band) in root samples collected from 3-week-old maize seedlings shown in Fig. 4e. Total protein loading control is shown in the lower panel. **i** Above around dry biomass of 1-week-old maize seedling grown under full nutrient conditions when treated with ValA (orange) or without ValA treatment (green) p values are determined by t-test; two tailed; Data were collected from one trial with N = 5 independent plants measured.) Bar heights are mean value across all plants. Error bars indicate standard deviation centered on mean values. **j** A biological replicate of the immunoblot shown in Fig. 4d measuring the abundance of both free ATG8 (upper band) and the ATG8-PE conjugate (lower band) in root samples collected from root samples from 1-week-old maize seedlings grown under optimal nutrient conditions with or without ValA treatment. Total protein loading control is shown in the lower panel. This procedure was repeated twice with equivalent results. *kDA = kiloDaltons*. Source data are provided as a Source Data file.

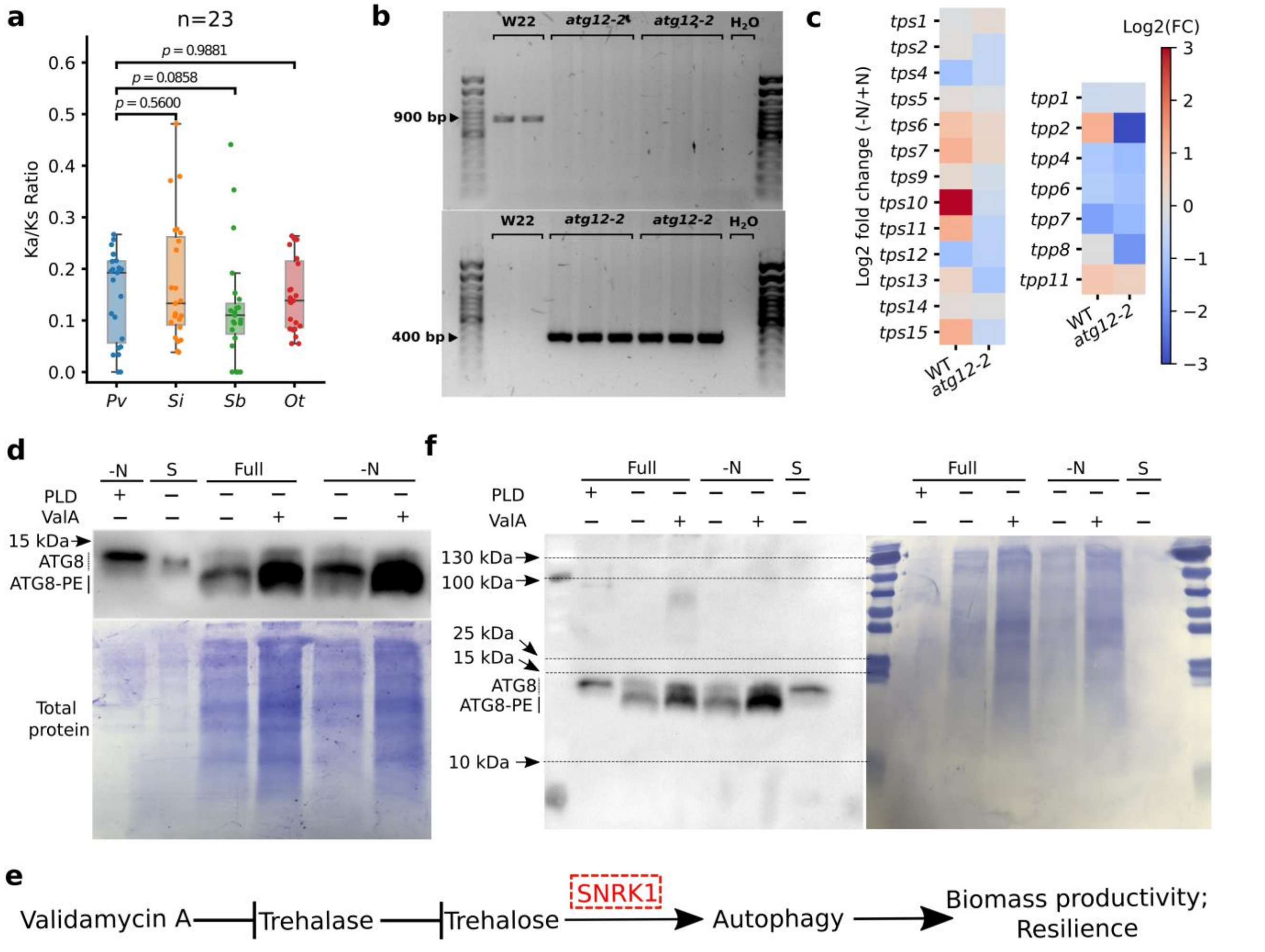

Supplementary Figure 14. Autophagy knock down mutant *atg12-2* and the role of autophagy in the ValA mediated increase biomass accumulation. a Distribution of observed Ka/Ks ratios (ratios of nonsynonymous to synonymous substitution rates) for a set of N=23 autophagy-related genes (ATG) syntenically conserved across paspalum (Pv, *Paspalum vaginatum*, blue), foxtail millet (Si, *Setaria italica*, orange), sorghum (Sb, *Sorghum bicolor*, green), and Oropetium (Ot,*Oropetium thomaeum*, red). Statistical comparisons were conducted using Wilcoxon signed-rank test. Box plots in this panel are defined as described in Fig. 1. **b** Amplification of the wild type *atg12* gene and *mu* insertion event from the DNA template extracted from six randomly selected *atg12-2* seedlings and two randomly selected wild type background W22 seedlings. **c** Changes in the expression levels of annotated maize genes encoding trehalose-6-phosphate synthase (TPS) or trehalose-6-phosphate phosphatase (TPP) in response to nitrogen deficient treatment in both the wild type and *atg12-2* mutant backgrounds. Expression fold changes were calculated from RNAseq libraries for three independent biological replicates for each species under each conditions by DESeq2(v1.22.2) with p values corrected for fdr <0.05 using raw RNA sequence data retrieved from NCBI BioProject PRJNA449498. **d** A replicate of the immunoblot shown in Fig. 6 measuring the abundance of both free ATG8 (upper band) and the ATG8-PE conjugate (lower band) in root samples collected from 3-week-old maize plants. Total protein loading control is shown in the lower panel. Mem: membrane protein; S: Cellular soluable fraction separated by ultracentrifugation in which no ATG8-PE exists. PLD: phospholipase D treatment. kDa : kiloDalton. This procedure was repeated twice with equivalent results. **e** A diagram showing internal accumulation of trehalose in root induced by trehalase inhibitor validamycin A activates SNRK1 which promotes autophagy and lead to enhanced biomass productivity in maize under both nutrient optimal and nitrogen deficit conditions. **f** Full original gel image of the membranes incubated in anti-ATG8 anitbody and the total loading of membrane proteins of the data shown in Fig. 6. The ladders are biomass markers indicating the biomass of the bands shown in the membrane. The ATG8 and ATG8-PE bands are between 10kDa and 15 kDa. Source data are provided as a Source Data file.

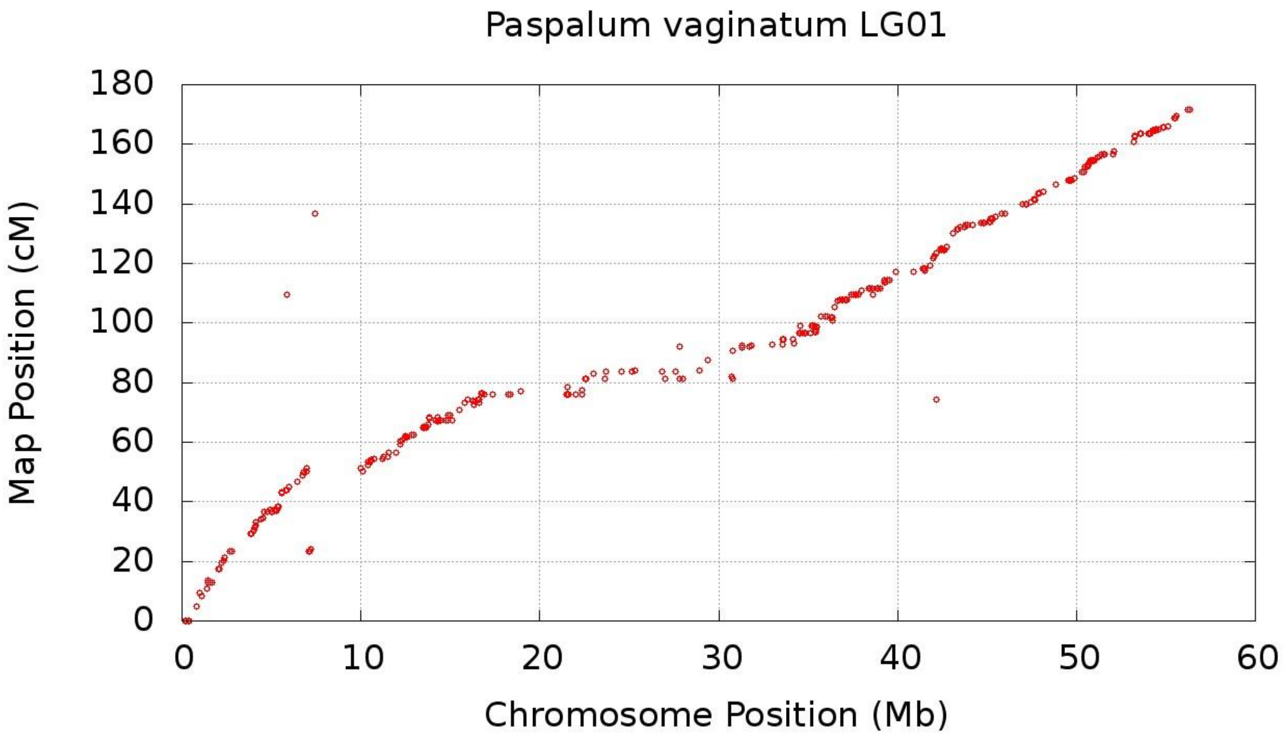

Supplementary Figure 15. Marker map placements on the *Paspalum vaginatum* chromosome 1.

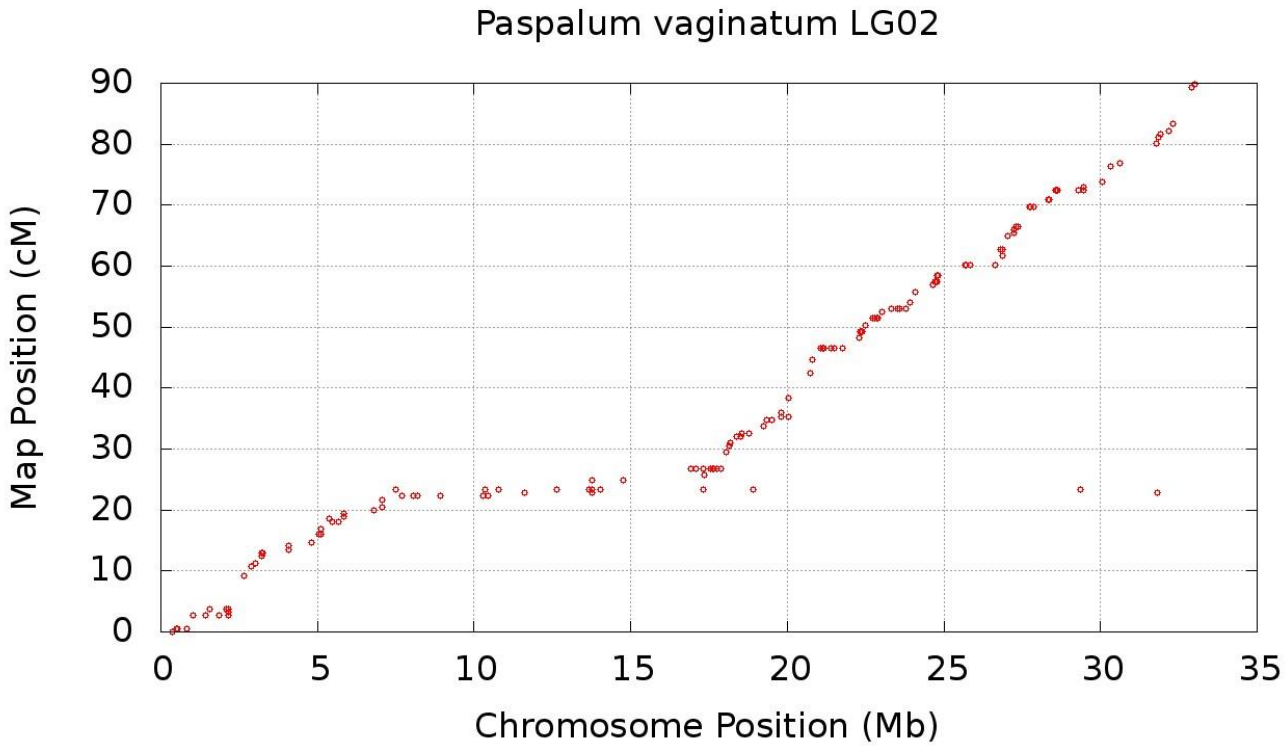

Supplementary Figure 16. Marker map placements on the *Paspalum vaginatum* chromosome 2.

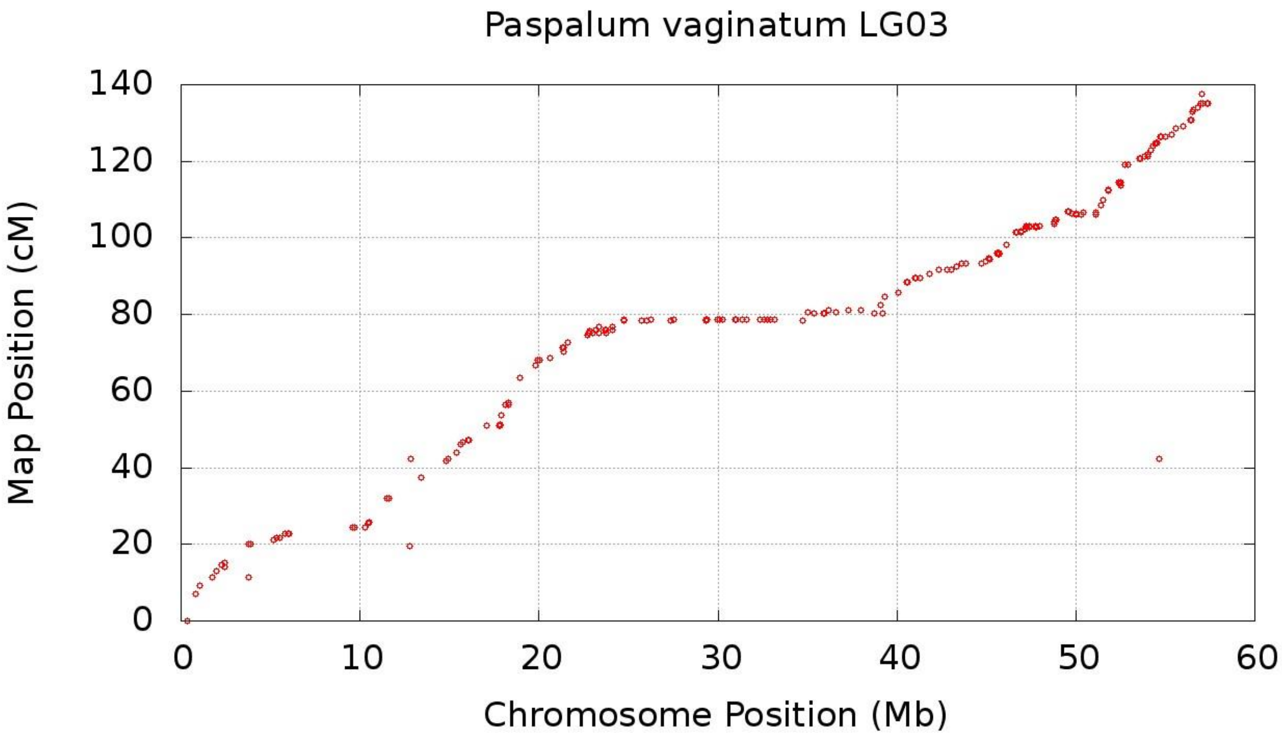

.

Supplementary Figure 17. Marker map placements on the *Paspalum vaginatum* chromosome 3.

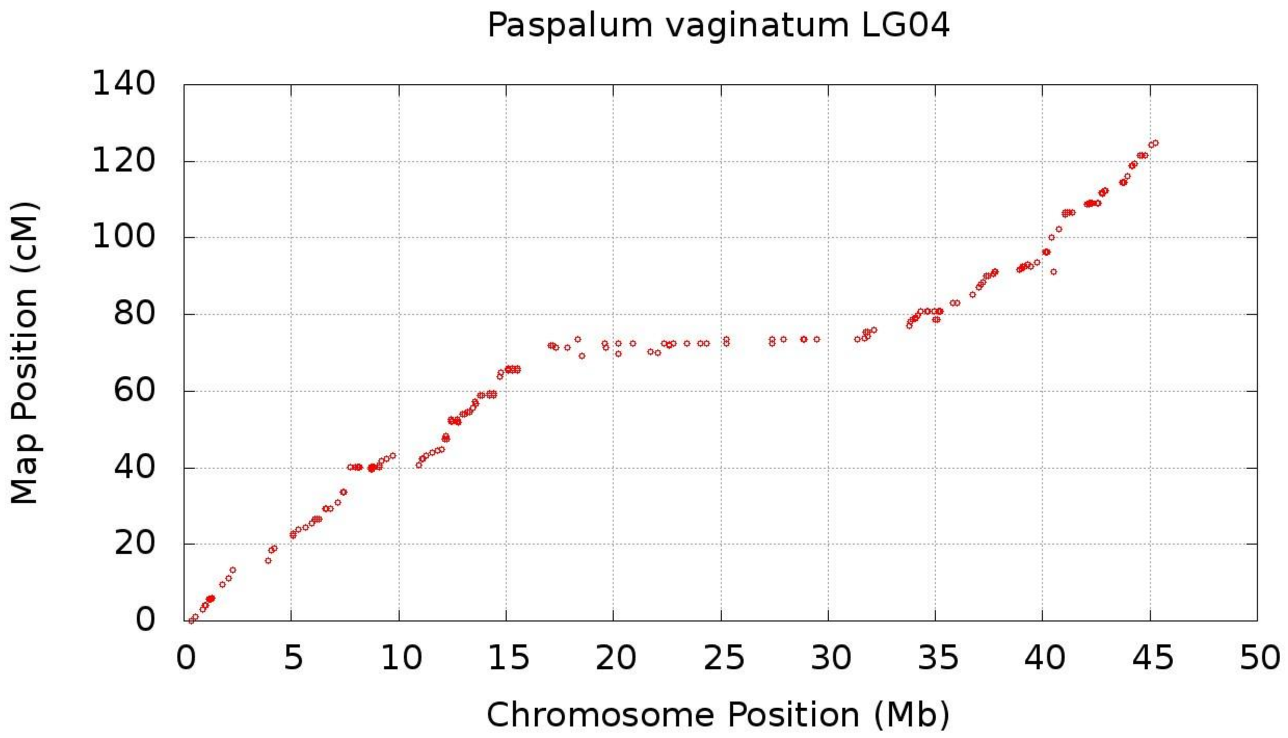

Supplementary Figure 18. Marker map placements on the *Paspalum vaginatum* chromosome 4.

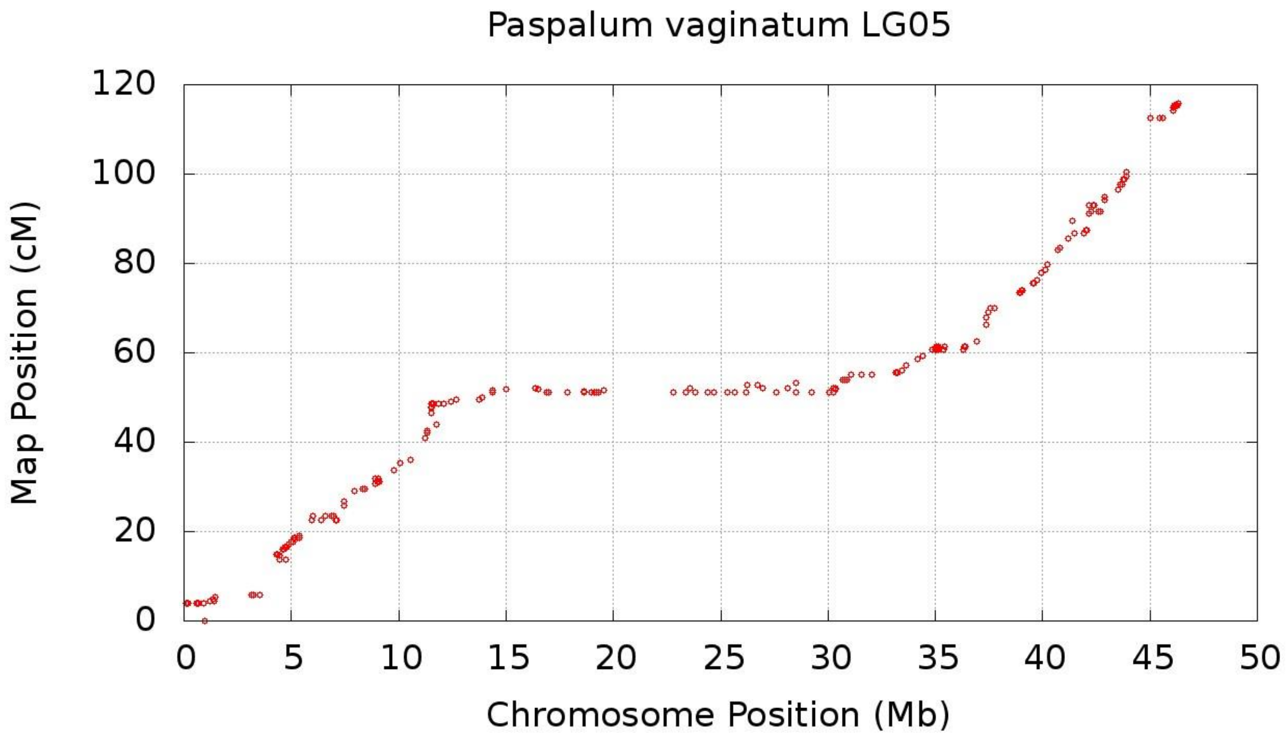

Supplementary Figure 19. **Marker map placements on the *Paspalum vaginatum* chromosome 5.**

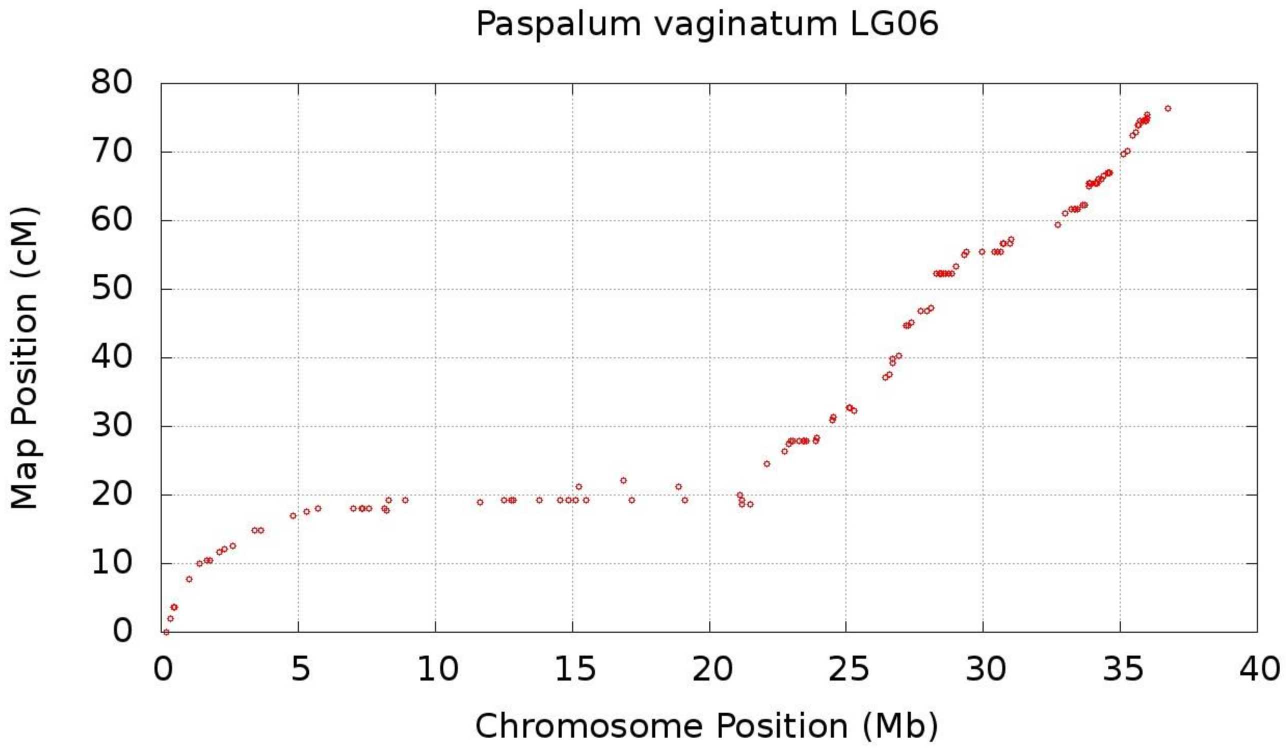

Supplementary Figure 20. Marker map placements on the *Paspalum vaginatum* chromosome 6.

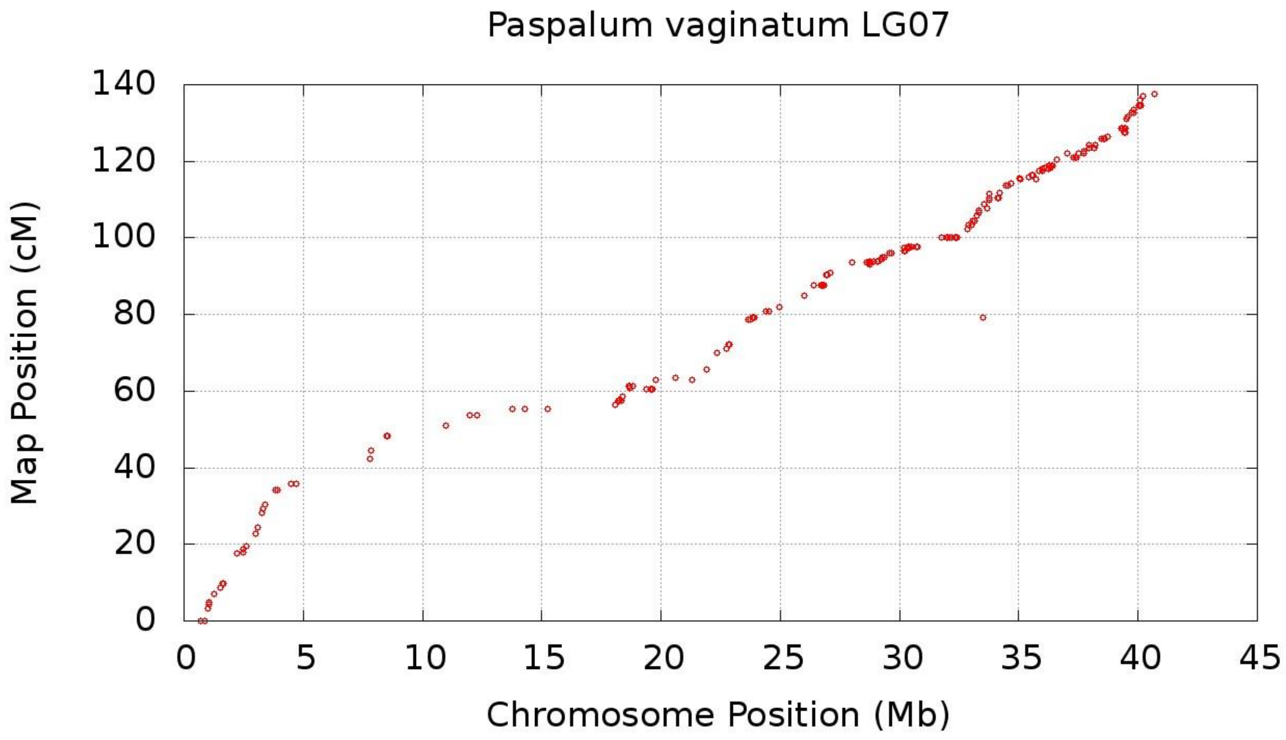

Supplementary Figure 21. Marker map placements on the *Paspalum vaginatum* chromosome 7.

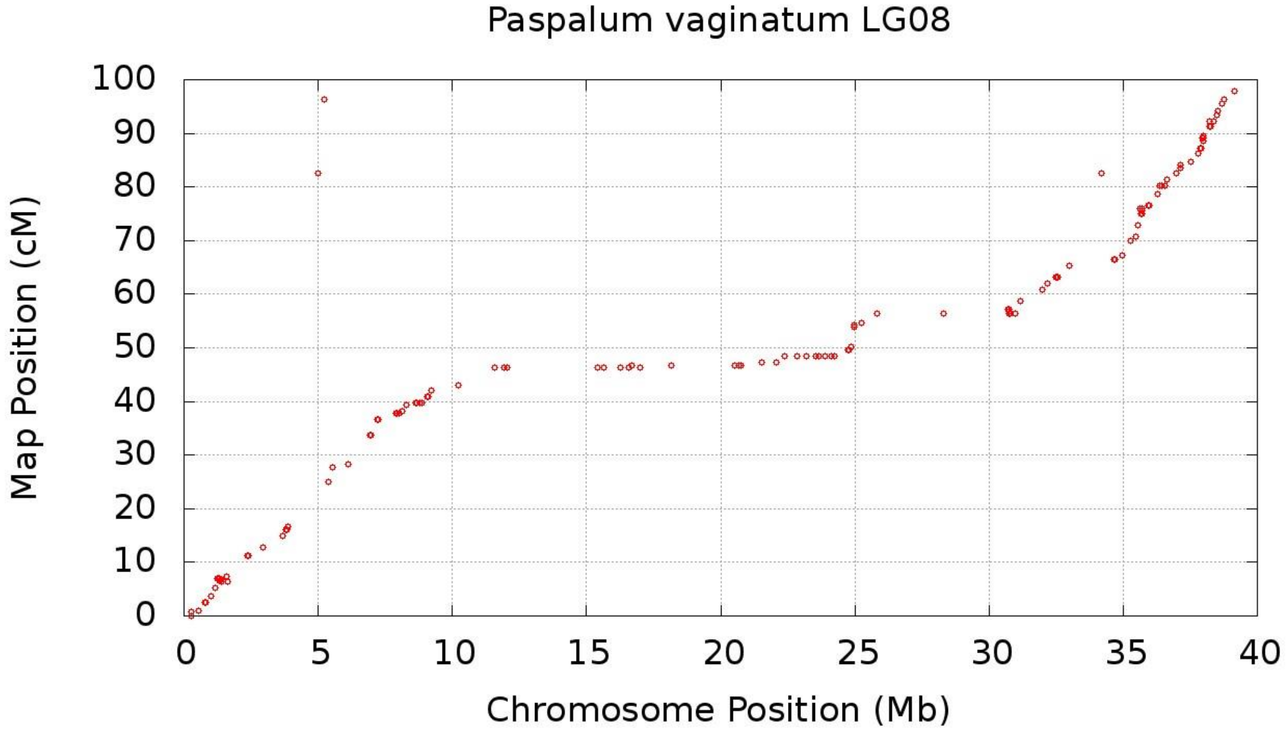

Supplementary Figure 22. Marker map placements on the *Paspalum vaginatum* chromosome 8.

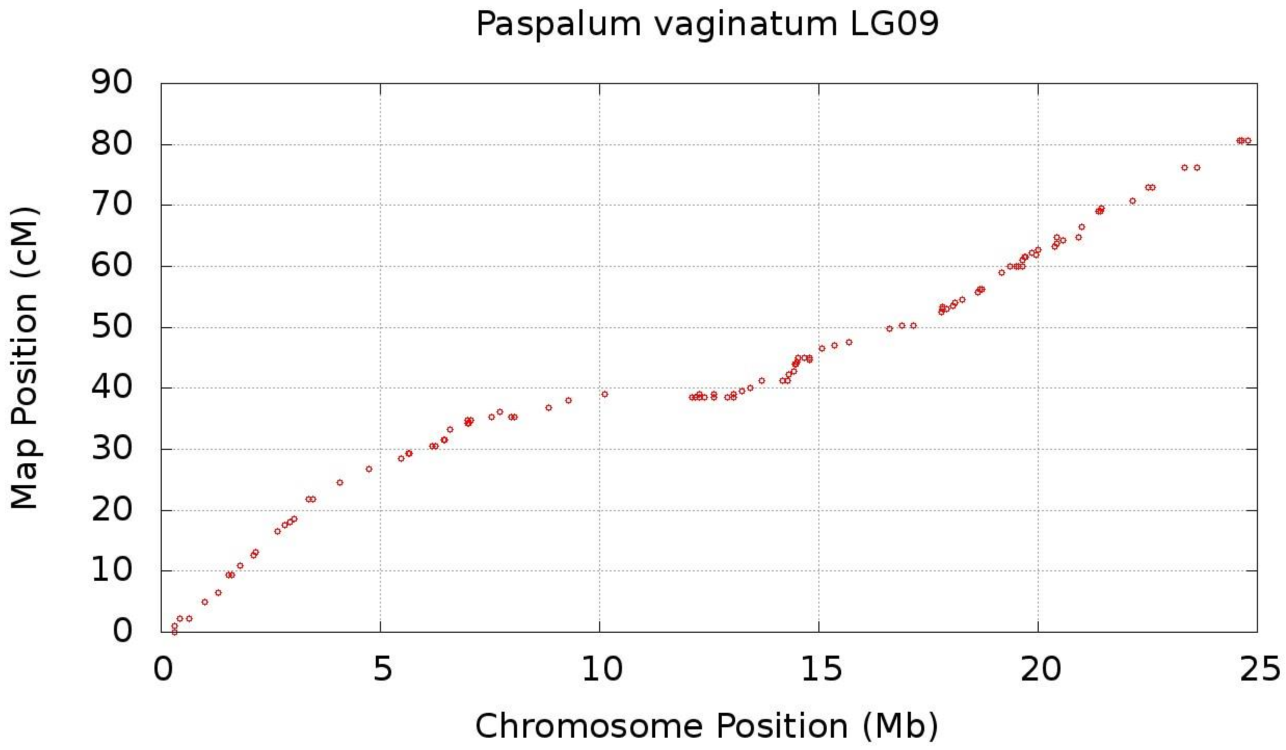

Supplementary Figure 23. Marker map placements on the *Paspalum vaginatum* chromosome 9.

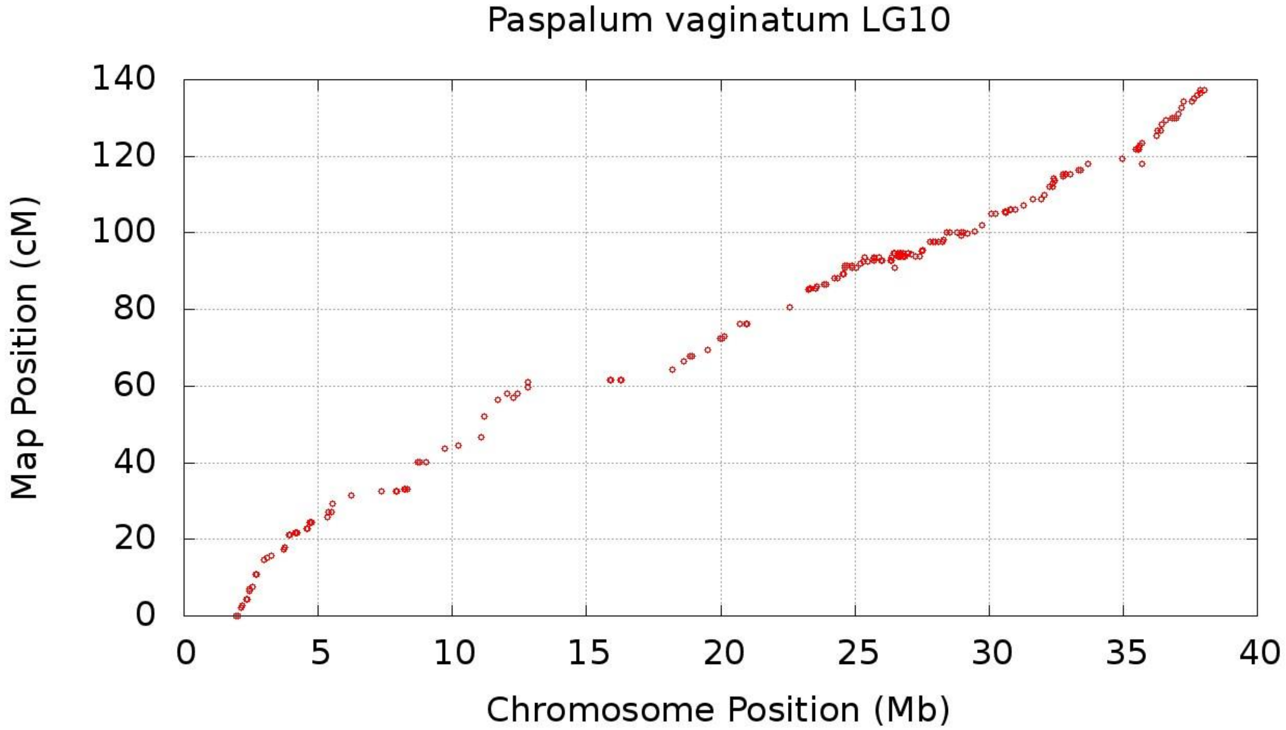

Supplementary Figure 24. Marker map placements on the *Paspalum vaginatum* chromosome 10.
