# Supplementary material for: Genome of Paspalum vaginatum and the role of trehalose mediated autophagy in increasing maize biomass
Source: Nat Commun. 2022 Dec 13;13:7731. doi: 10.1038/s41467-022-35507-8 (PMC9747981; doi:10.1038/s41467-022-35507-8)
Supplement: Supplementary file 3 — Description of Additional Supplementary Files [file 41467_2022_35507_MOESM3_ESM.pdf]

Description of Additional Supplementary Files

File Name: Supplementary Data 1
Description: Markers used for paspalum genetic map construction.

File Name: Supplementary Data 2
Description: Calculated Ka, Ks, and Ka/Ks ratios for each grass gene employed in this study.

File Name: Supplementary Data 3
Description: Genes from the paspalum specific expanded gene families and GO terms enriched among these genes. Gene family expansion was identified using CAFE5 which models rates of change among gene families with a a discrete approximation of the gamma (γ) distribution[7,8]

File Name: Supplementary Data 4
Description: Raw fold change values for each metabolite plotted in Figure 3; P values are determined by two-sided t test.
