# Supplementary material for: Genome of Paspalum vaginatum and the role of trehalose mediated autophagy in increasing maize biomass
Source: Nat Commun. 2022 Dec 13;13:7731. doi: 10.1038/s41467-022-35507-8 (PMC9747981; doi:10.1038/s41467-022-35507-8)
Supplement: Supplementary file 8 — Reporting Summary [file 41467_2022_35507_MOESM8_ESM.pdf]

nature portfolio

Corresponding author(s): James Schnable

Last updated by author(s): Nov 30, 2022

Reporting Summary

Nature Portfolio wishes to improve the reproducibility of the work that we publish. This form provides structure for consistency and transparency in reporting. For further information on Nature Portfolio policies, see our Editorial Policies and the Editorial Policy Checklist.

Statistics

For all statistical analyses, confirm that the following items are present in the figure legend, table legend, main text, or Methods section.

n/a	Confirmed	
☐	☒	The exact sample size (*n*) for each experimental group/condition, given as a discrete number and unit of measurement
☐	☒	A statement on whether measurements were taken from distinct samples or whether the same sample was measured repeatedly
☐	☒	The statistical test(s) used AND whether they are one- or two-sided<br>*Only common tests should be described solely by name; describe more complex techniques in the Methods section.*
☒	☐	A description of all covariates tested
☐	☒	A description of any assumptions or corrections, such as tests of normality and adjustment for multiple comparisons
☐	☒	A full description of the statistical parameters including central tendency (e.g. means) or other basic estimates (e.g. regression coefficient) AND variation (e.g. standard deviation) or associated estimates of uncertainty (e.g. confidence intervals)
☐	☒	For null hypothesis testing, the test statistic (e.g. *F*, *t*, *r*) with confidence intervals, effect sizes, degrees of freedom and *P* value noted<br>*Give P values as exact values whenever suitable.*
☒	☐	For Bayesian analysis, information on the choice of priors and Markov chain Monte Carlo settings
☒	☐	For hierarchical and complex designs, identification of the appropriate level for tests and full reporting of outcomes
☒	☐	Estimates of effect sizes (e.g. Cohen's *d*, Pearson's *r*), indicating how they were calculated

Our web collection on statistics for biologists contains articles on many of the points above.

Software and code

Policy information about availability of computer code

Data collection	No Software is used for data collection
Data analysis	The reads were assembled using MECAT(v1.1) and polished using QUIVER(v2.3.3); SNPs were called using GATK(v3.6)'s UnifiedGenotyper; Repeat sequences were masked by RepeatModeler; Transcript assemblies were generated using PERTRAN followed by PASA (v2.0.2); Proteomies were aligned to the repeat-masked genome using EXONERATE (v2.4.0); Independent sets of gene models were predicted using FGENESH+ (v3.1.1), FGENESH_EST (v2.6), EXONERATE and AUGUST (v3.1.0) as implemented in BRAKER1(v1.6) and the in-house PASA assembly open reading frames finder tool from JGI; The GO germs were assigned to genes based on the InterProScan (5.26-65.0) results. Orthologs were were aligned using condon-based aligner ParaAT(v2.0); alignments were removed and trimmed using Gblocks(v0.91b); phylogenies were constructed based on the alignments using PAxML(v8.2);Tree topologies were visualize and classified using DensiTree(v2.2.6). IQ-TREE(v1.6) was used to construct ML phylogeny based on concatenated alignment. Divergence time estimates was performed using the r8s(v1.81);Syntenic orthologous gene pairs were identified and visualized using LAST(v1170) and JCVI(v1.0.14) respectively. Branch specific KaKs ratio was estimated using the 'codeml' package implemented in PAML(v4.7). MAPMAKER is used to produce the genetic map from the HA, AH and HH sets. ; Gene family clusters were identified using orthoFinder and the families under contraction and expansion were identified using CAFE5; Quality control of the RNAseq reads were performed using FASTQC(v0.11); RNAseq reads were trimmed using trimmomatic (v0.33); Trimmed reads were mapped to the reference genome using STAR (v2.7); Transcripts Per Million (TPM) table was generated using Kallisto (v0.46.2); Read counts for each RNAseq library were determined using the software HTSeq (version 0.9); Differential gene expression analysis was performed using DESeq2; GO term enrichment analysis was performed using GOATOOLS(v1.2.3); Codes used to generate graphs included in this manuscript are freely available via https://github.com/xiaoguanghuan123/paspalumGenome

For manuscripts utilizing custom algorithms or software that are central to the research but not yet described in published literature, software must be made available to editors and reviewers. We strongly encourage code deposition in a community repository (e.g. GitHub). See the Nature Portfolio guidelines for submitting code & software for further information.

Data

Policy information about availability of data

All manuscripts must include a data availability statement. This statement should provide the following information, where applicable:

- Accession codes, unique identifiers, or web links for publicly available datasets
- A description of any restrictions on data availability
- For clinical datasets or third party data, please ensure that the statement adheres to our policy

The genome sequence and annotation is accessible via Phytozome v13: https://phytozome-next.jgi.doe.gov/info/Pvaginatum_793 RNAseq data for root tissues of paspalum, maize and sorghum under three nutrient conditions are available at NCBI under the BioProject: PRJNA746310 (https://www.ncbi.nlm.nih.gov/bioproject/PRJNA746310). RNAseq data for root tissues of maize seedlings under three nutrient conditions with or without validamycin A treatment are also available at NCBI under the BioProject: PRJNA746310 (https://www.ncbi.nlm.nih.gov/bioproject/PRJNA746310). RNAseq data for paspalum shoots/rhizome is available at NCBI with SRA accession SRR10230104(https://www.ncbi.nlm.nih.gov/sra/SRR10230104/); SRR10230108 (https://www.ncbi.nlm.nih.gov/sra/?term=SRR10230108); SRR10230122 (https://www.ncbi.nlm.nih.gov/sra/?term=SRR10230122); SRR10230130 (https://www.ncbi.nlm.nih.gov/sra/?term=SRR10230130). RNAseq data of maize wild type and atg12-1 mutant is available at NCBI with BioProject accession ID: PRJNA449498 (https://www.ncbi.nlm.nih.gov/bioproject/?term=PRJNA449498).The genome assembly and sequence data of Paspalum vaginatum are deposited in NCBI: BioProject PRJNA234783 (https://www.ncbi.nlm.nih.gov/bioproject/?term=PRJNA234783); genome accession in Genbank:JAPDNB000000000 (https://www.ncbi.nlm.nih.gov/nuccore/JAPDNB000000000). Source data are provided with this paper. All of the scripts and raw data used for generating main figures and codes for generating supplementary figures in this study have been deposited in Zenodo (https://zenodo.org/badge/latestdoi/505987272) and can be accessed from Github (https://github.com/xiaoguanghuan123/paspalumGenome)

Human research participants

Policy information about studies involving human research participants and Sex and Gender in Research.

Reporting on sex and gender	NA
Population characteristics	NA
Recruitment	NA
Ethics oversight	NA

Note that full information on the approval of the study protocol must also be provided in the manuscript.

Field-specific reporting

Please select the one below that is the best fit for your research. If you are not sure, read the appropriate sections before making your selection.

☒ Life sciences ☐ Behavioural & social sciences ☐ Ecological, evolutionary & environmental sciences

For a reference copy of the document with all sections, see nature.com/documents/nr-reporting-summary-flat.pdf

Life sciences study design

All studies must disclose on these points even when the disclosure is negative.

Sample size	In this study, the three biological replicates for seedling roots from different experimental conditions were used to profile transcriptomes. For plant greenhouse experiments, all of the experiments were conducted using 5 or more than 5 plants in three independent replicates. Experiments for adult plants grown for 67 days under -N and Full conditions with or without validamycin A was conducted for one trial with 3 plants under each conditions. A similar study conducted in rice by Wada, Shinya, et al., 2015 investigating role of autophagy in plant biomass and NUE used measurements from three independent biological replicates each with 4 technical replicates. Li, Pinghua, et al., 2010 used two biological replicates from four developmental zones of maize leaves to profile their transcriptomes.
Data exclusions	No data is excluded
Replication	Repeatability of transcriptomes are shown in figure S5 and all other replicated experiments described in the manuscript were successful
Randomization	We randomized the position of the pots for the plants received the different nutrient solutions and treatments to avoid the potential light coverage and intensity variations in the greenhouse.
Blinding	All of the plants need to be clearly labeled and avoid any cross-contamination during the harvesting, this is critical for testing the difference between the ones grown under different nutrient conditions with or without treatments. Therefore, blinding sample collection is impossible for this study.

Reporting for specific materials, systems and methods

We require information from authors about some types of materials, experimental systems and methods used in many studies. Here, indicate whether each material, system or method listed is relevant to your study. If you are not sure if a list item applies to your research, read the appropriate section before selecting a response.

Materials & experimental systems

n/a	Involved in the study
☐	☒ Antibodies
☒	☐ Eukaryotic cell lines
☒	☐ Palaeontology and archaeology
☒	☐ Animals and other organisms
☒	☐ Clinical data
☒	☐ Dual use research of concern

Methods

n/a	Involved in the study
☐	☐ ChIP-seq
☐	☒ Flow cytometry
☐	☐ MRI-based neuroimaging

Antibodies

Antibodies used	Anti-At ATG8 antibodies (1:1000 dilution)(Agrisera, Vännäs, Sweden; AS14 2769).
Validation	This antibody has been used and the results have been published in many high-profiled journals. Such as Li et al, 2015, The Plant Cell; McLoughlin et al, 2018, Nature Plants

ChIP-seq

Data deposition

☐ Confirm that both raw and final processed data have been deposited in a public database such as GEO.

☐ Confirm that you have deposited or provided access to graph files (e.g. BED files) for the called peaks.

Data access links<br>*May remain private before publication.*	*For "Initial submission" or "Revised version" documents, provide reviewer access links. For your "Final submission" document, provide a link to the deposited data.*
Files in database submission	*Provide a list of all files available in the database submission.*
Genome browser session<br>(e.g. UCSC)	*Provide a link to an anonymized genome browser session for "Initial submission" and "Revised version" documents only, to enable peer review. Write "no longer applicable" for "Final submission" documents.*

Methodology

Replicates	*Describe the experimental replicates, specifying number, type and replicate agreement.*
Sequencing depth	*Describe the sequencing depth for each experiment, providing the total number of reads, uniquely mapped reads, length of reads and whether they were paired- or single-end.*
Antibodies	*Describe the antibodies used for the ChIP-seq experiments; as applicable, provide supplier name, catalog number, clone name, and lot number.*
Peak calling parameters	*Specify the command line program and parameters used for read mapping and peak calling, including the ChIP, control and index files used.*
Data quality	*Describe the methods used to ensure data quality in full detail, including how many peaks are at FDR 5% and above 5-fold enrichment.*
Software	*Describe the software used to collect and analyze the ChIP-seq data. For custom code that has been deposited into a community repository, provide accession details.*

Flow Cytometry

Plots

Confirm that:

☒ The axis labels state the marker and fluorochrome used (e.g. CD4-FITC).

☒ The axis scales are clearly visible. Include numbers along axes only for bottom left plot of group (a 'group' is an analysis of identical markers).

☒ All plots are contour plots with outliers or pseudocolor plots.

☒ A numerical value for number of cells or percentage (with statistics) is provided.

Methodology

Sample preparation	One leaf per plant of paspalum (PI 509022) and sorghum (BTx623) were harvested and kept on ice until processing. A CyStain Propidium Iodide Absolute P kit (Sysmex, Milton Keynes, United Kingdom) was used to extract and stain the nuclei from a 1 cm2 piece of leaf tissue following the manufacturer’s instructions. To reduce the amount of cellular debris in the extracts, samples were passed through a 30 µm filter (CellTrics®-Sysmex Partec, Goerlitz, Germany) and centrifuged at 600Å~g before final staining. Sorghum was used as an internal standard to reduce the staining variability between samples.
Instrument	The stained samples were then analyzed on a CytoFLEX flow cytometer (Beckman Coulter, Brea, CA, USA) following a two-hour incubation at 4 °C. The propidium iodide was excited with a yellow-green 561 nm laser and detected with a 585/42 emission filter.
Software	n/a
Cell population abundance	*Describe the abundance of the relevant cell populations within post-sort fractions, providing details on the purity of the samples and how it was determined.*
Gating strategy	*Describe the gating strategy used for all relevant experiments, specifying the preliminary FSC/SSC gates of the starting cell population, indicating where boundaries between "positive" and "negative" staining cell populations are defined.*

☒ Tick this box to confirm that a figure exemplifying the gating strategy is provided in the Supplementary Information.

Magnetic resonance imaging

Experimental design

Design type	*Indicate task or resting state; event-related or block design.*
Design specifications	*Specify the number of blocks, trials or experimental units per session and/or subject, and specify the length of each trial or block (if trials are blocked) and interval between trials.*
Behavioral performance measures	*State number and/or type of variables recorded (e.g. correct button press, response time) and what statistics were used to establish that the subjects were performing the task as expected (e.g. mean, range, and/or standard deviation across subjects).*

Acquisition

Imaging type(s)	*Specify: functional, structural, diffusion, perfusion.*
Field strength	*Specify in Tesla*
Sequence & imaging parameters	*Specify the pulse sequence type (gradient echo, spin echo, etc.), imaging type (EPI, spiral, etc.), field of view, matrix size, slice thickness, orientation and TE/TR/flip angle.*
Area of acquisition	*State whether a whole brain scan was used OR define the area of acquisition, describing how the region was determined.*

Diffusion MRI ☐ Used ☐ Not used

Preprocessing

Preprocessing software	*Provide detail on software version and revision number and on specific parameters (model/functions, brain extraction, segmentation, smoothing kernel size, etc.).*
Normalization	*If data were normalized/standardized, describe the approach(es): specify linear or non-linear and define image types used for transformation OR indicate that data were not normalized and explain rationale for lack of normalization.*
Normalization template	*Describe the template used for normalization/transformation, specifying subject space or group standardized space (e.g. original Talairach, MNI305, ICBM152) OR indicate that the data were not normalized.*

Noise and artifact removal

Describe your procedure(s) for artifact and structured noise removal, specifying motion parameters, tissue signals and physiological signals (heart rate, respiration).

Volume censoring

Define your software and/or method and criteria for volume censoring, and state the extent of such censoring.

Statistical modeling & inference

Model type and settings

Specify type (mass univariate, multivariate, RSA, predictive, etc.) and describe essential details of the model at the first and second levels (e.g. fixed, random or mixed effects; drift or auto-correlation).

Effect(s) tested

Define precise effect in terms of the task or stimulus conditions instead of psychological concepts and indicate whether ANOVA or factorial designs were used.

Specify type of analysis: ☐ Whole brain ☐ ROI-based ☐ Both

Statistic type for inference
(See Eklund et al. 2016)

Specify voxel-wise or cluster-wise and report all relevant parameters for cluster-wise methods.

Correction

Describe the type of correction and how it is obtained for multiple comparisons (e.g. FWE, FDR, permutation or Monte Carlo).

Models & analysis

n/a	Involved in the study
☐	☐ Functional and/or effective connectivity
☐	☐ Graph analysis
☐	☐ Multivariate modeling or predictive analysis

Functional and/or effective connectivity

Report the measures of dependence used and the model details (e.g. Pearson correlation, partial correlation, mutual information).

Graph analysis

Report the dependent variable and connectivity measure, specifying weighted graph or binarized graph, subject- or group-level, and the global and/or node summaries used (e.g. clustering coefficient, efficiency, etc.).

Multivariate modeling and predictive analysis

Specify independent variables, features extraction and dimension reduction, model, training and evaluation metrics.
