# Supplementary material for: Genome of Paspalum vaginatum and the role of trehalose mediated autophagy in increasing maize biomass
Source: Nat Commun. 2022 Dec 13;13:7731. doi: 10.1038/s41467-022-35507-8 (PMC9747981; doi:10.1038/s41467-022-35507-8)
Supplement: Supplementary file 9 — Source Data [file 41467_2022_35507_MOESM9_ESM.zip › Supplementary Figure 1_1.pdf]

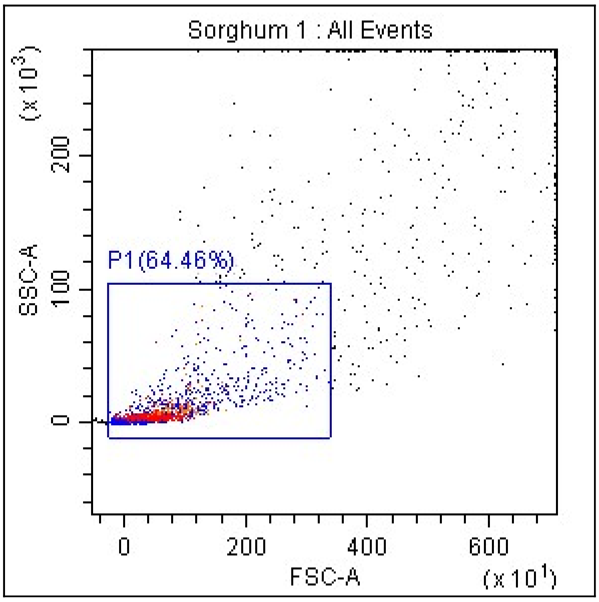

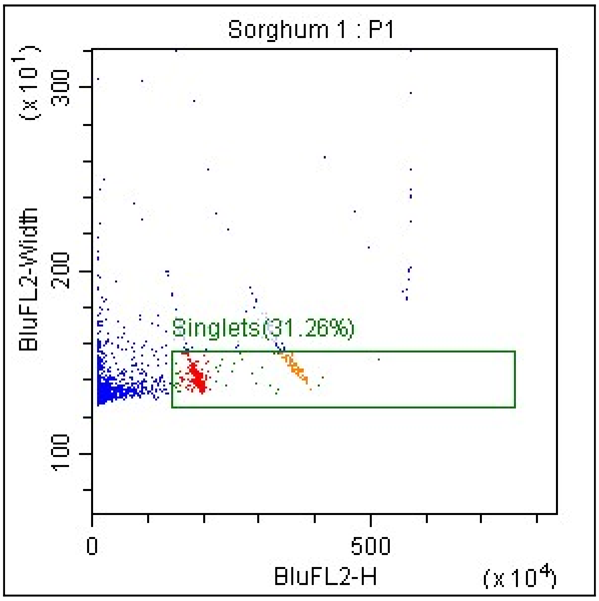

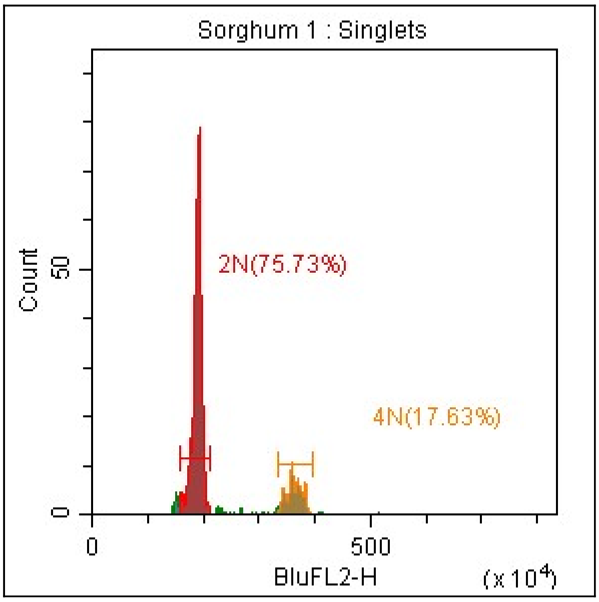

Tube Name: Sorghum 1

Sample ID:

Population	Events	% Total	% Parent	Median BluFL2-A
All Events	2392	100.00%	100.00%	626292.6
P1	1542	64.46%	64.46%	763938.5
Singlets	482	20.15%	31.26%	2617686.3
2N	365	15.26%	75.73%	2603848.5
4N	85	3.55%	17.63%	5209476.5

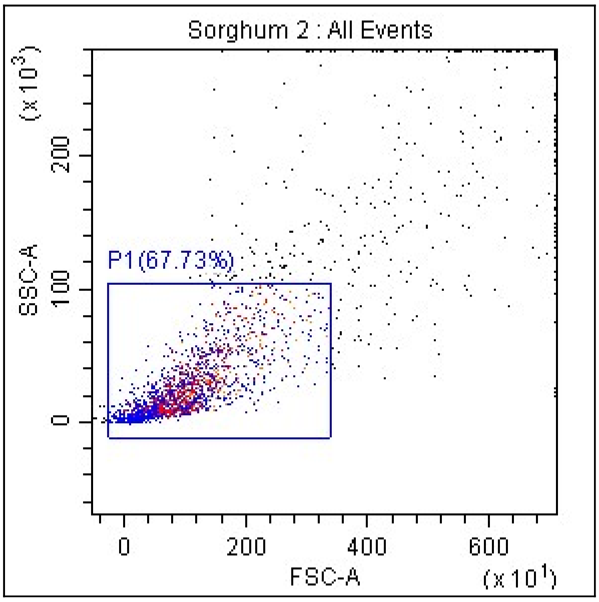

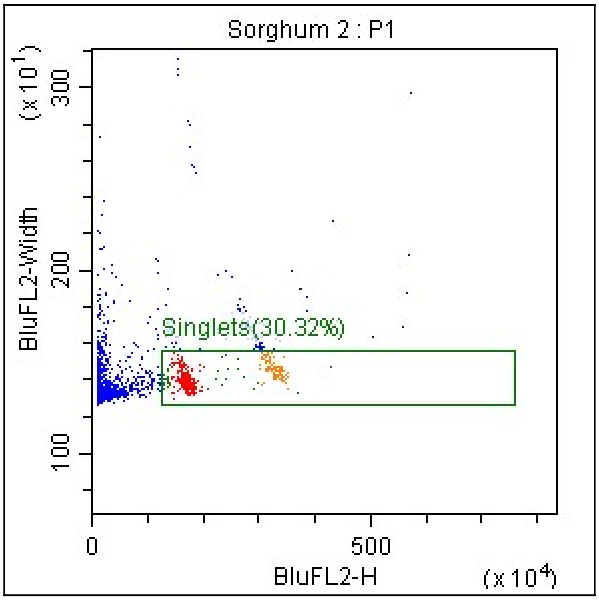

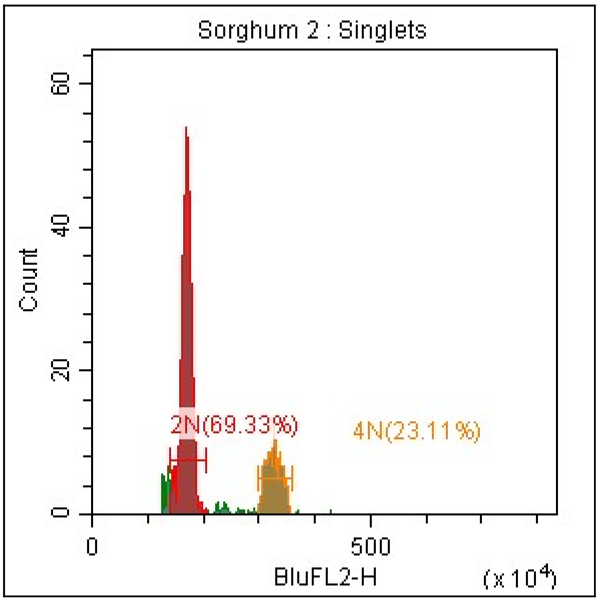

Tube Name: Sorghum 2

Sample ID:

Population	Events	% Total	% Parent	Median BluFL2-A
All Events	2318	100.00%	100.00%	688558.0
P1	1570	67.73%	67.73%	586642.3
Singlets	476	20.53%	30.32%	2362474.5
2N	330	14.24%	69.33%	2329966.3
4N	110	4.75%	23.11%	4673319.0

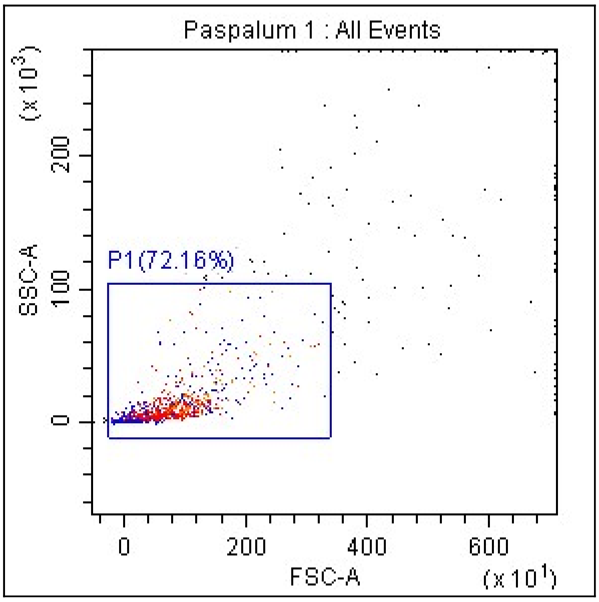

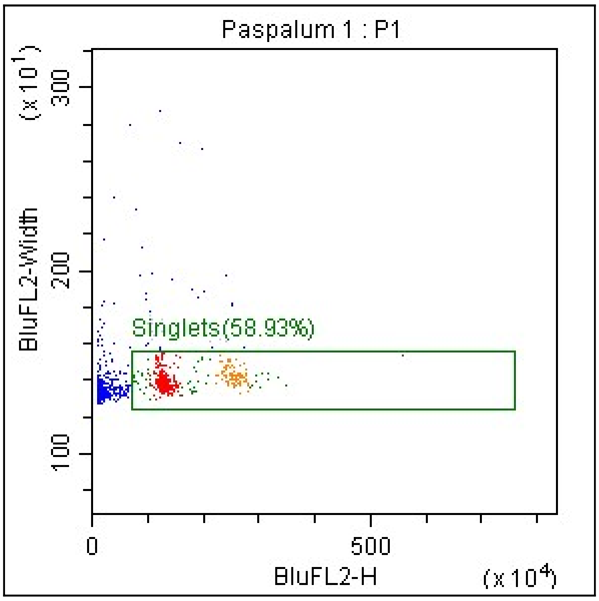

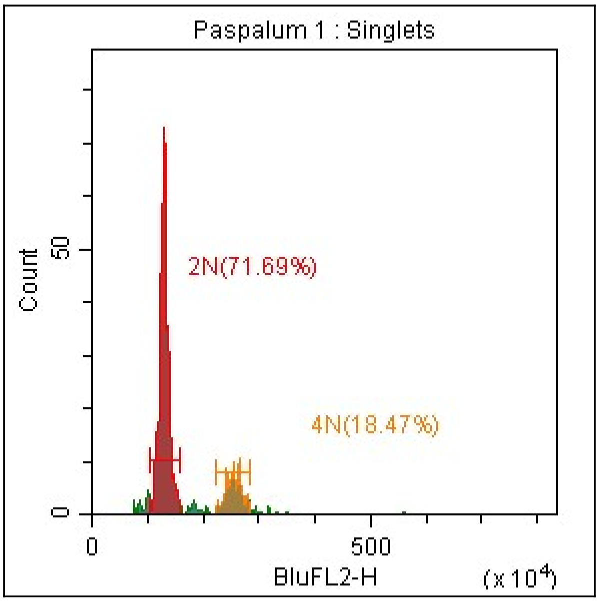

Tube Name: Paspalum 1

Sample ID:

Population	Events	% Total	% Parent	Median BluFL2-A
All Events	1171	100.00%	100.00%	1634971.3
P1	845	72.16%	72.16%	1708328.4
Singlets	498	42.53%	58.93%	1786378.6
2N	357	30.49%	71.69%	1755360.3
4N	92	7.86%	18.47%	3562112.0

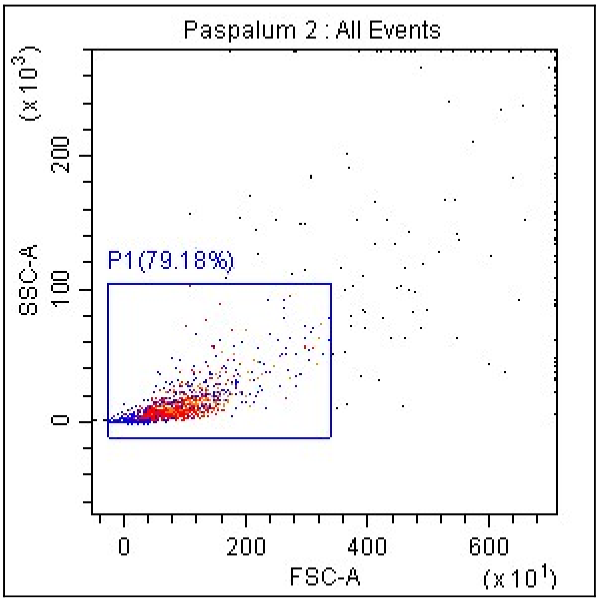

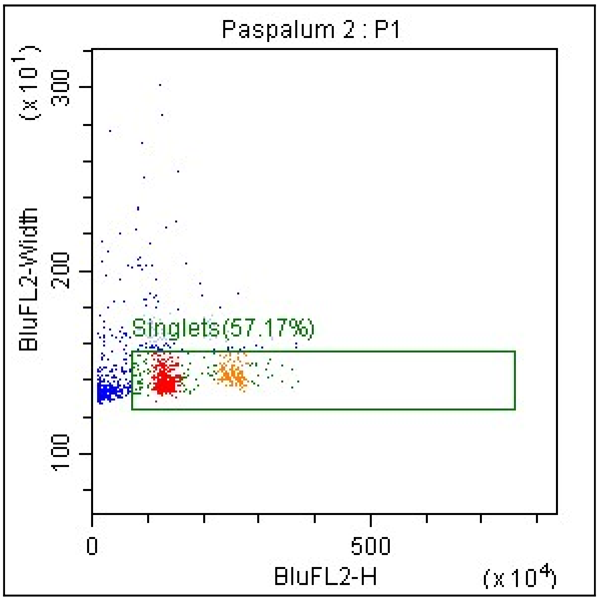

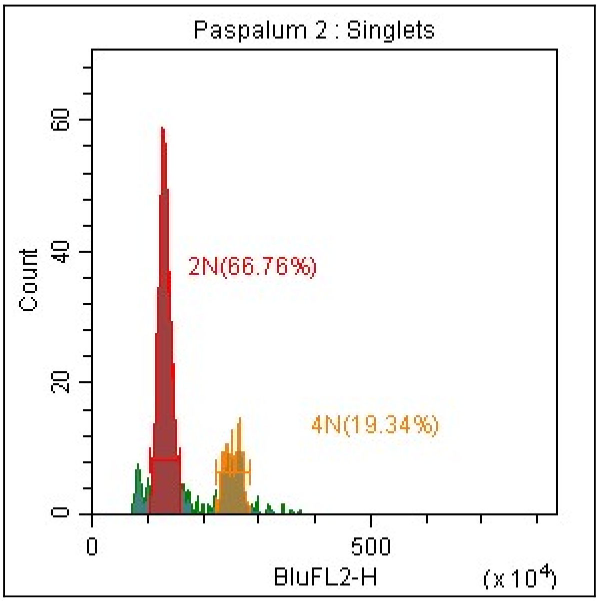

Tube Name: Paspalum 2

Sample ID:

Population	Events	% Total	% Parent	Median BluFL2-A
All Events	1542	100.00%	100.00%	1671738.8
P1	1221	79.18%	79.18%	1702893.1
Singlets	698	45.27%	57.17%	1831275.5
2N	466	30.22%	66.76%	1778143.3
4N	135	8.75%	19.34%	3576831.0
