# Supplementary material for: Genome of Paspalum vaginatum and the role of trehalose mediated autophagy in increasing maize biomass
Source: Nat Commun. 2022 Dec 13;13:7731. doi: 10.1038/s41467-022-35507-8 (PMC9747981; doi:10.1038/s41467-022-35507-8)
Supplement: Supplementary file 9 — Source Data [file 41467_2022_35507_MOESM9_ESM.zip › Supplementary Figure 1_2.pdf]

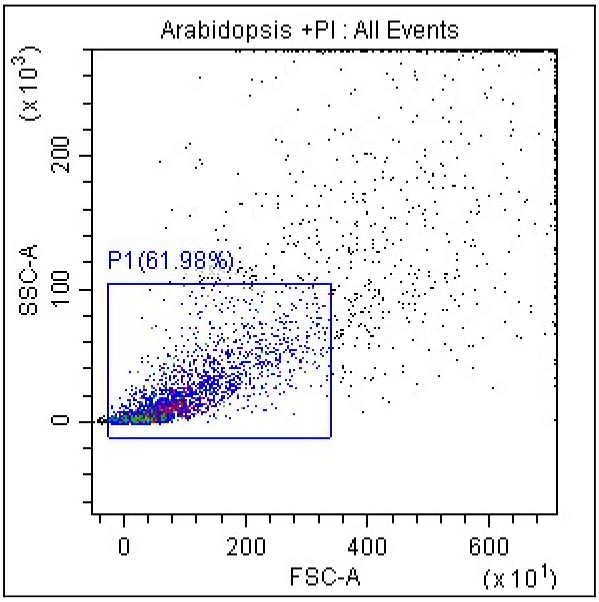

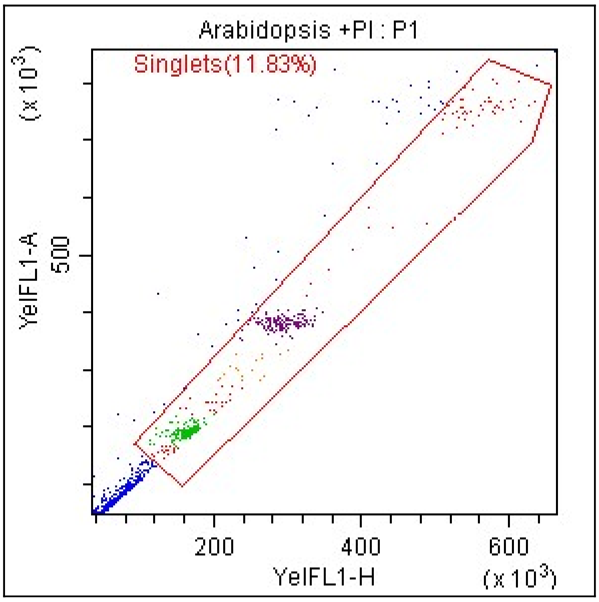

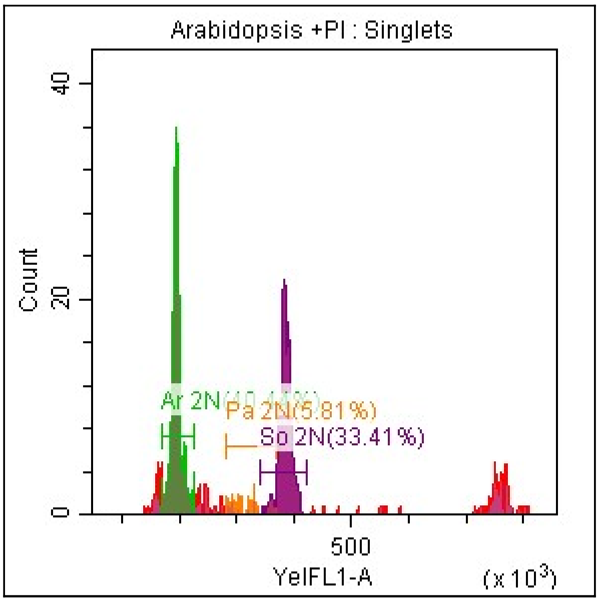

Tube Name: Arabidopsis +PI

Sample ID:

Population	Events	% Total	% Parent	Median YelFL1-A
All Events	5631	100.00%	100.00%	26251.7
P1	3490	61.98%	61.98%	20235.9
Singlets	413	7.33%	11.83%	282833.0
Ar 2N	167	2.97%	40.44%	194230.7
Pa 2N	24	0.43%	5.81%	320676.8
So 2N	138	2.45%	33.41%	383943.9

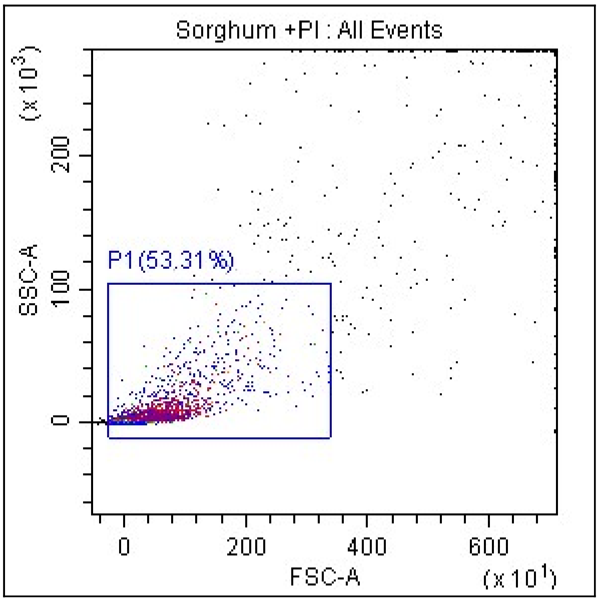

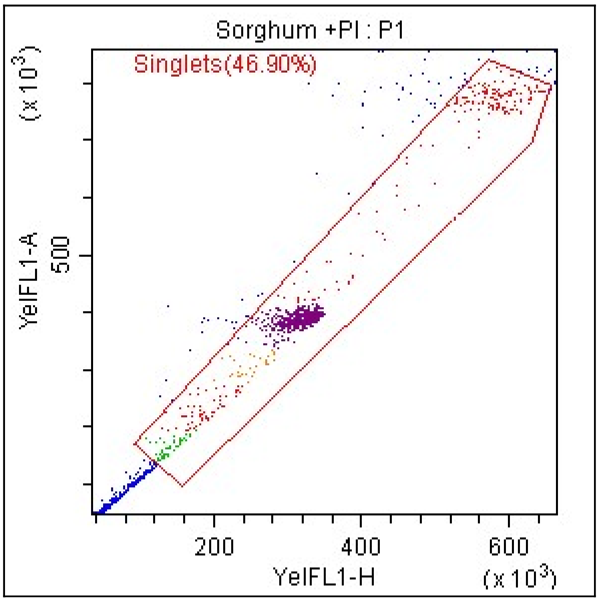

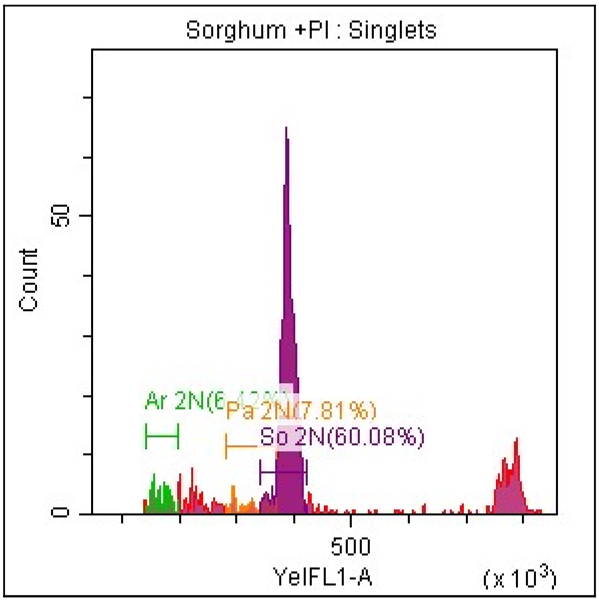

Tube Name: Sorghum +PI

Sample ID:

Population	Events	% Total	% Parent	Median BluFL2-A
All Events	3176	100.00%	100.00%	812185.0
P1	1693	53.31%	53.31%	1461838.5
Singlets	794	25.00%	46.90%	3446594.3
Ar 2N	51	1.61%	6.42%	1480420.1
Pa 2N	62	1.95%	7.81%	2991106.0
So 2N	477	15.02%	60.08%	3440455.0

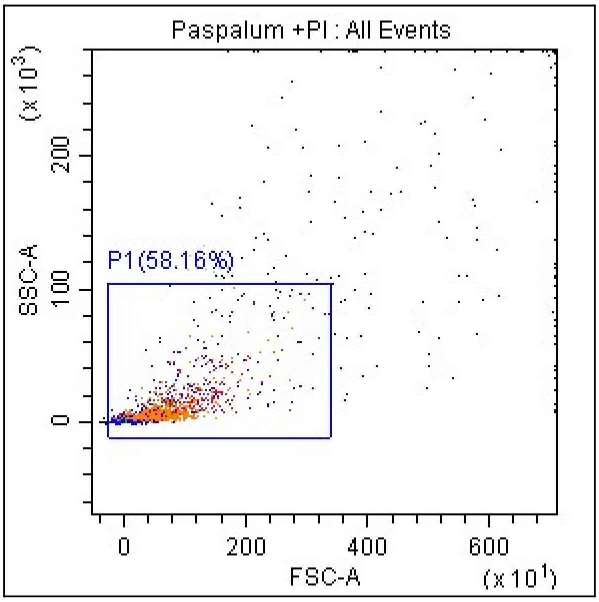

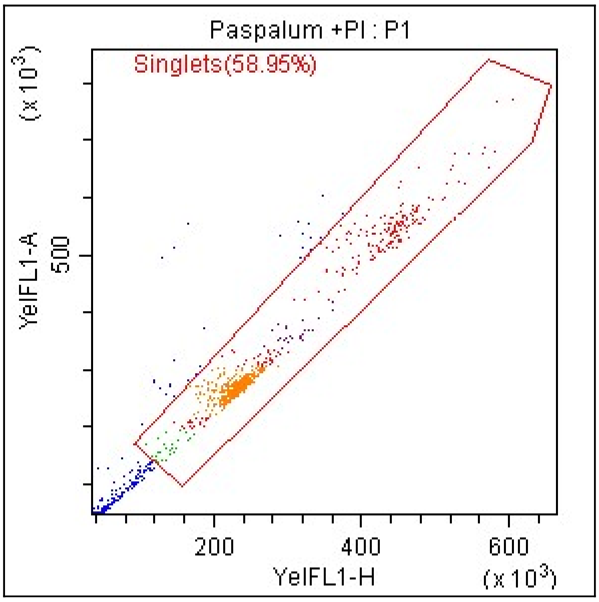

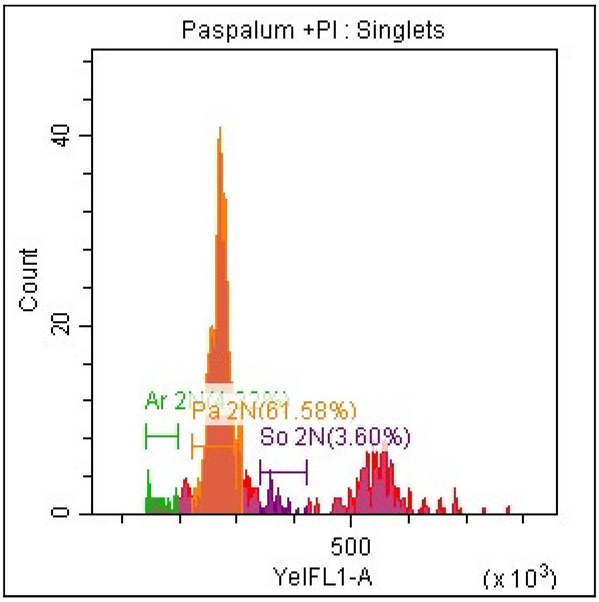

Tube Name: Paspalum +PI

Sample ID:

Population	Events	% Total	% Parent	Median BluFL2-A
All Events	2027	100.00%	100.00%	1079163.4
P1	1179	58.16%	58.16%	2288780.3
Singlets	695	34.29%	58.95%	2481352.0
Ar 2N	30	1.48%	4.32%	1461513.3
Pa 2N	428	21.11%	61.58%	2411919.0
So 2N	25	1.23%	3.60%	3197144.5

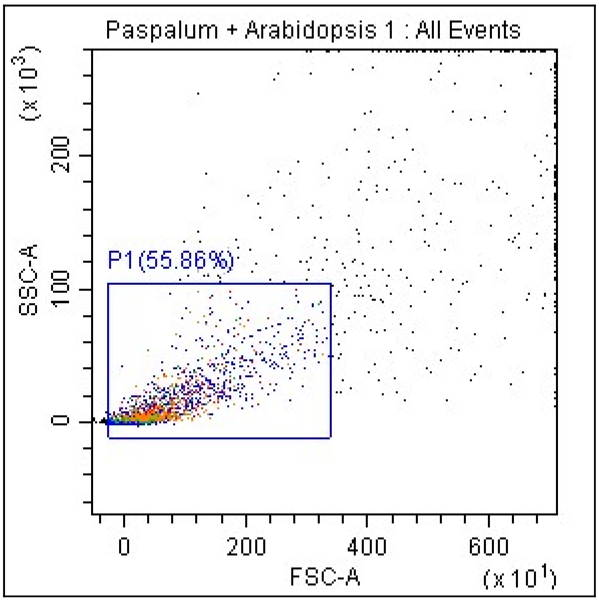

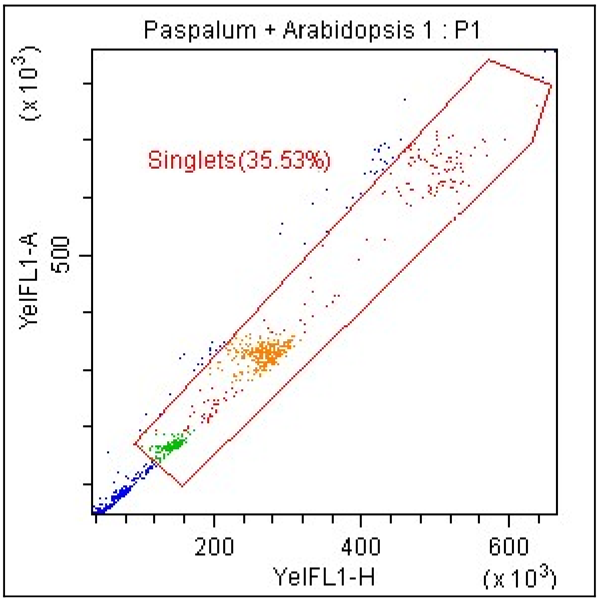

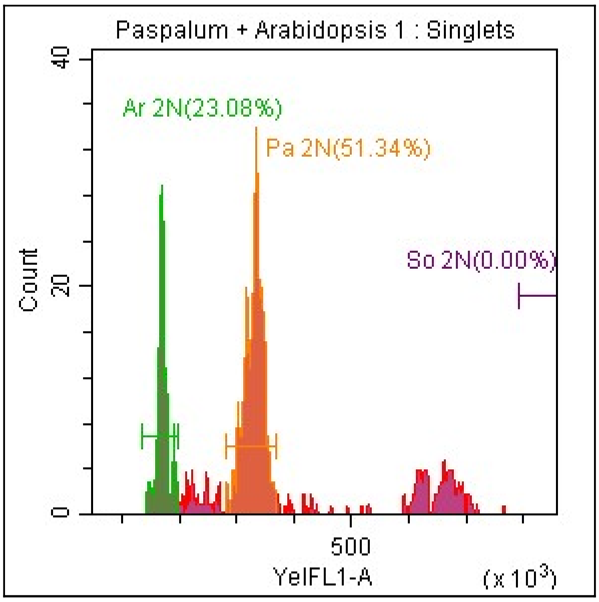

Tube Name: Paspalum + Arabidopsis 1

Sample ID:

Population	Events	% Total	% Parent	Median BluFL2-A
All Events	3013	100.00%	100.00%	803230.2
P1	1683	55.86%	55.86%	706539.9
Singlets	598	19.85%	35.53%	2897224.0
Ar 2N	138	4.58%	23.08%	1503482.0
Pa 2N	307	10.19%	51.34%	2935776.3
So 2N	0	0.00%	0.00%	####

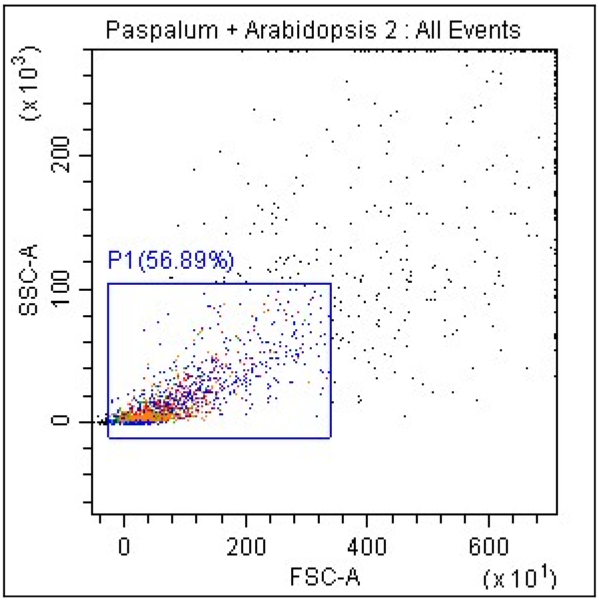

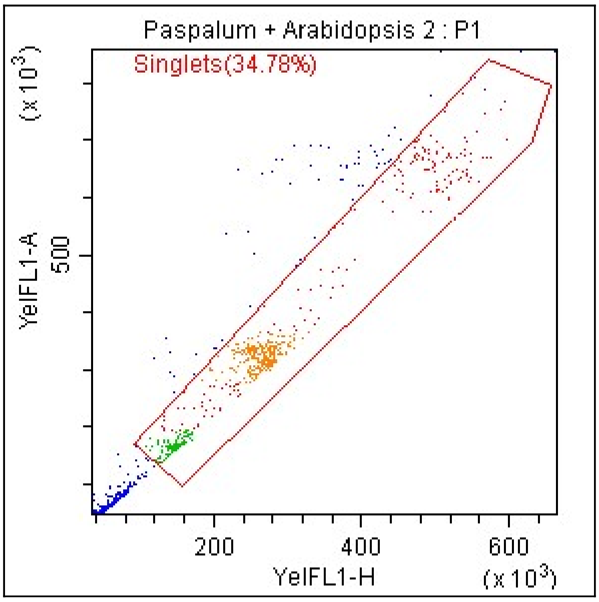

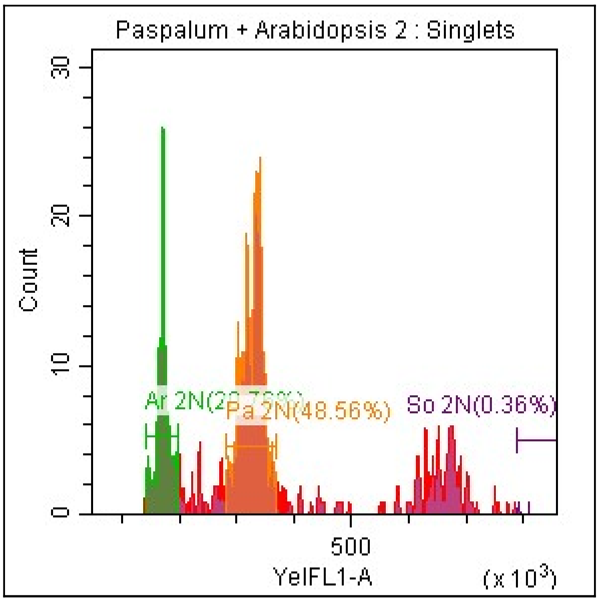

Tube Name: Paspalum + Arabidopsis 2

Sample ID:

Population	Events	% Total	% Parent	Median BluFL2-A
All Events	2800	100.00%	100.00%	784532.9
P1	1593	56.89%	56.89%	599498.5
Singlets	554	19.79%	34.78%	2903117.5
Ar 2N	115	4.11%	20.76%	1497832.1
Pa 2N	269	9.61%	48.56%	2921662.0
So 2N	2	0.07%	0.36%	7060511.0

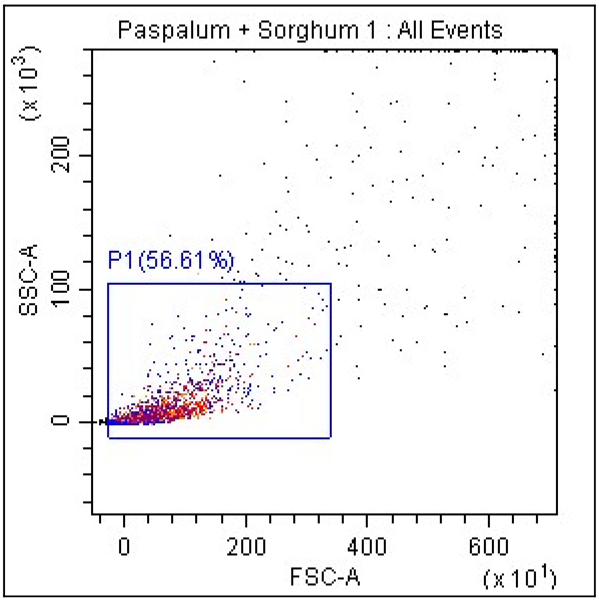

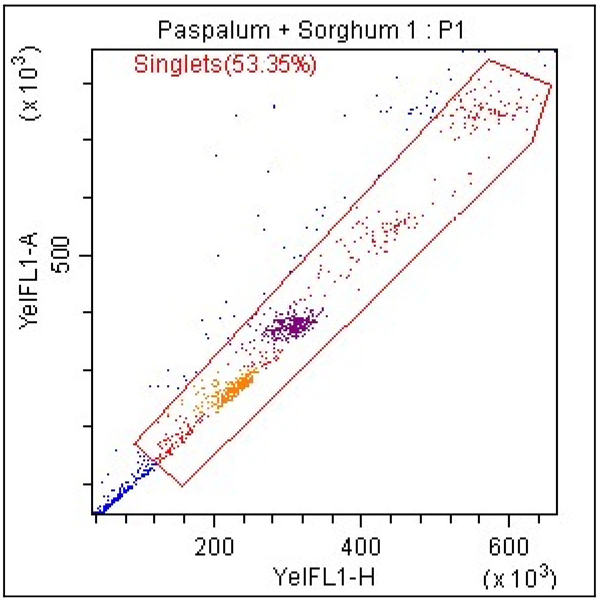

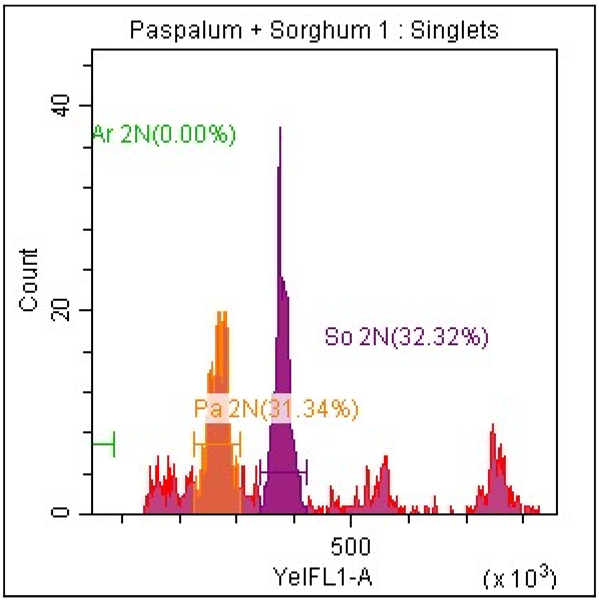

Tube Name: Paspalum + Sorghum 1
Sample ID:

Population	Events	% Total	% Parent	Median BluFL2-A
All Events	2715	100.00%	100.00%	1018371.9
P1	1537	56.61%	56.61%	2209777.3
Singlets	820	30.20%	53.35%	3228834.8
Ar 2N	0	0.00%	0.00%	####
Pa 2N	257	9.47%	31.34%	2379110.0
So 2N	265	9.76%	32.32%	3355933.0

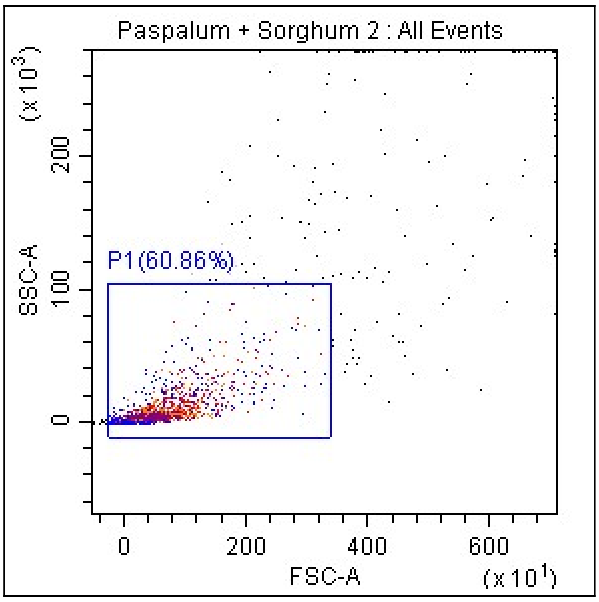

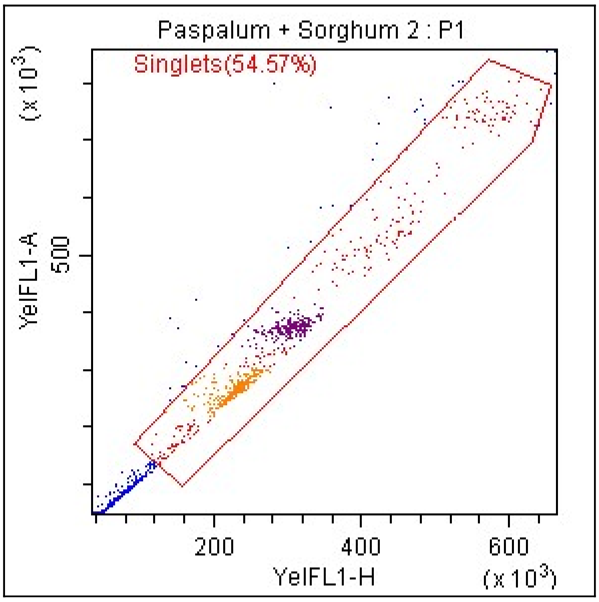

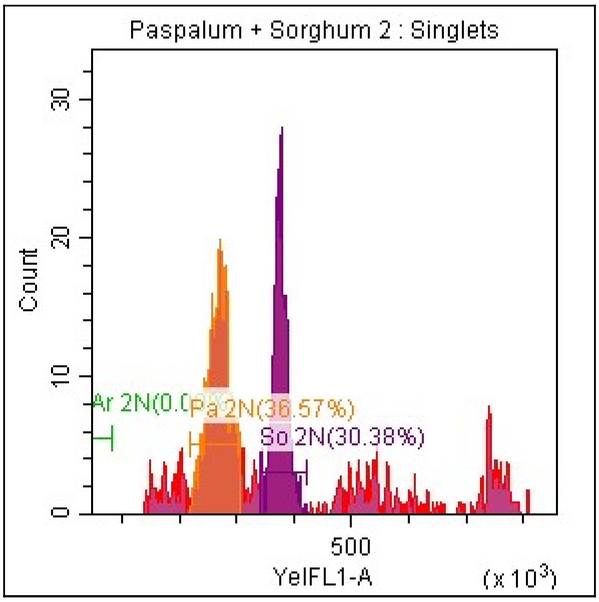

Tube Name: Paspalum + Sorghum 2
Sample ID:

Population	Events	% Total	% Parent	Median BluFL2-A
All Events	2141	100.00%	100.00%	987668.8
P1	1303	60.86%	60.86%	2216907.0
Singlets	711	33.21%	54.57%	3155066.3
Ar 2N	0	0.00%	0.00%	####
Pa 2N	260	12.14%	36.57%	2385221.0
So 2N	216	10.09%	30.38%	3316790.0
