# Supplementary material for: Genome of Paspalum vaginatum and the role of trehalose mediated autophagy in increasing maize biomass
Source: Nat Commun. 2022 Dec 13;13:7731. doi: 10.1038/s41467-022-35507-8 (PMC9747981; doi:10.1038/s41467-022-35507-8)
Supplement: Supplementary file 9 — Source Data [file 41467_2022_35507_MOESM9_ESM.zip › Uncropped Western Gel Image Scans.pptx]

## Slide 1
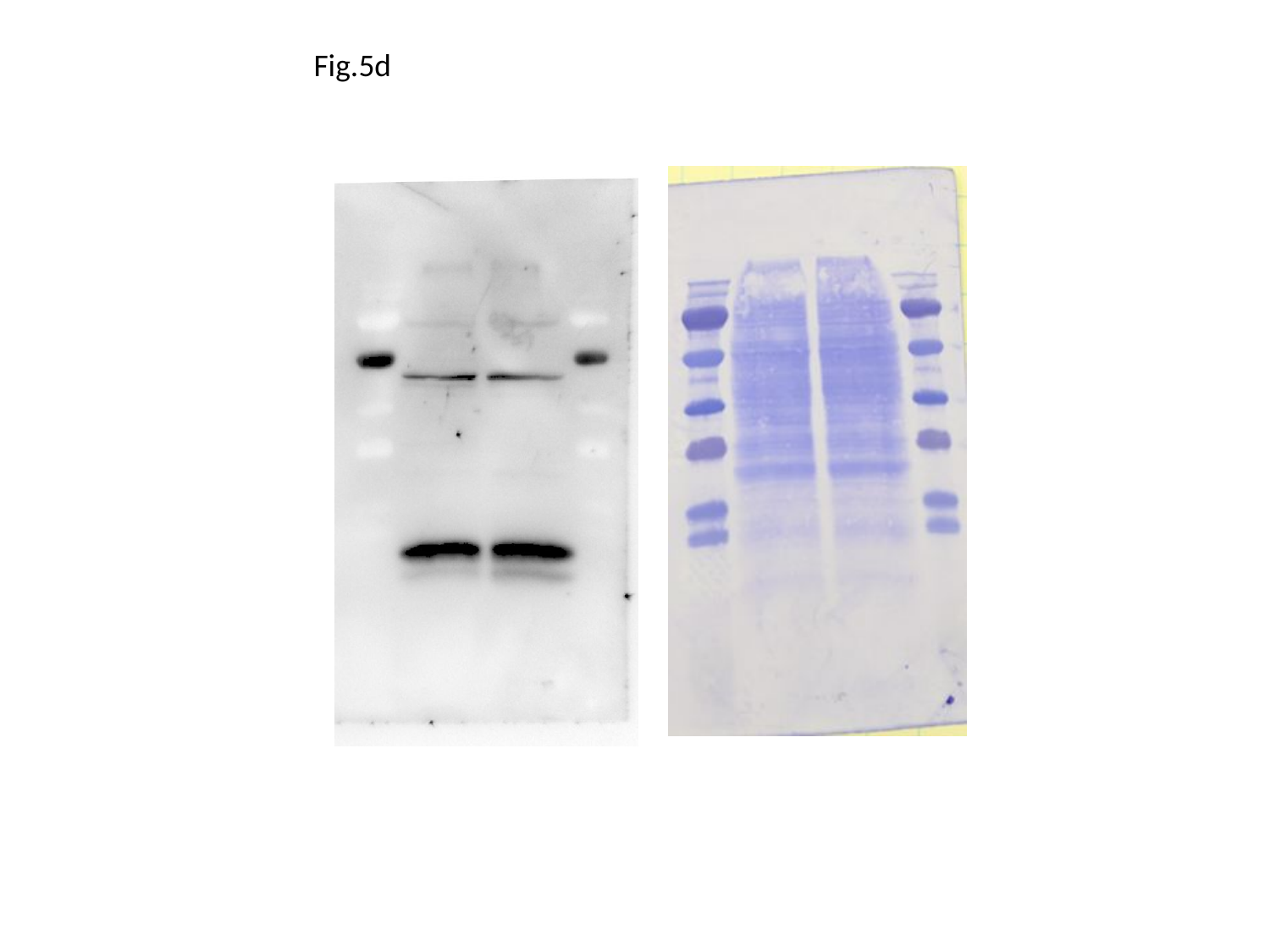

Fig.5d

## Slide 2
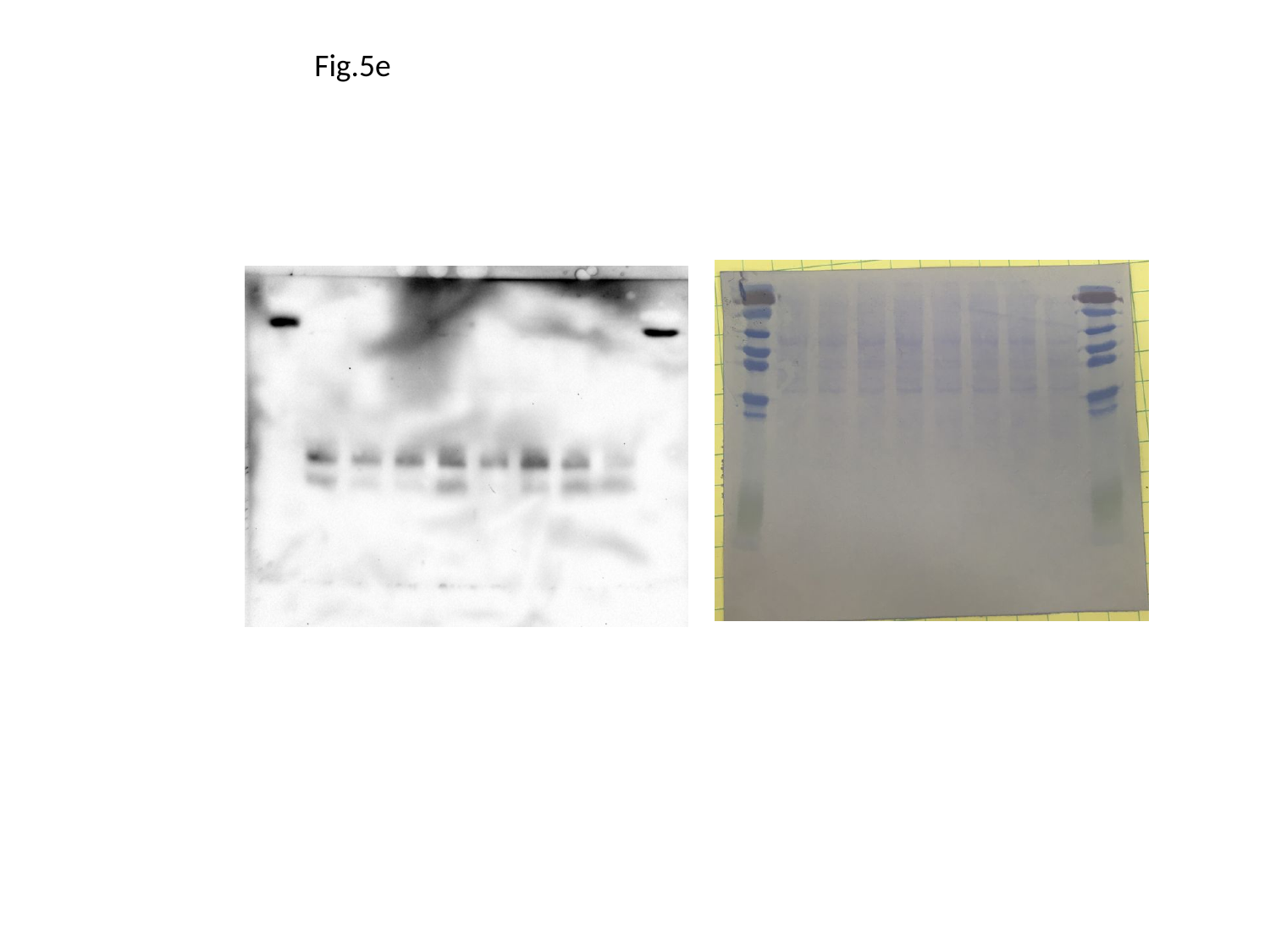

Fig.5e

## Slide 3
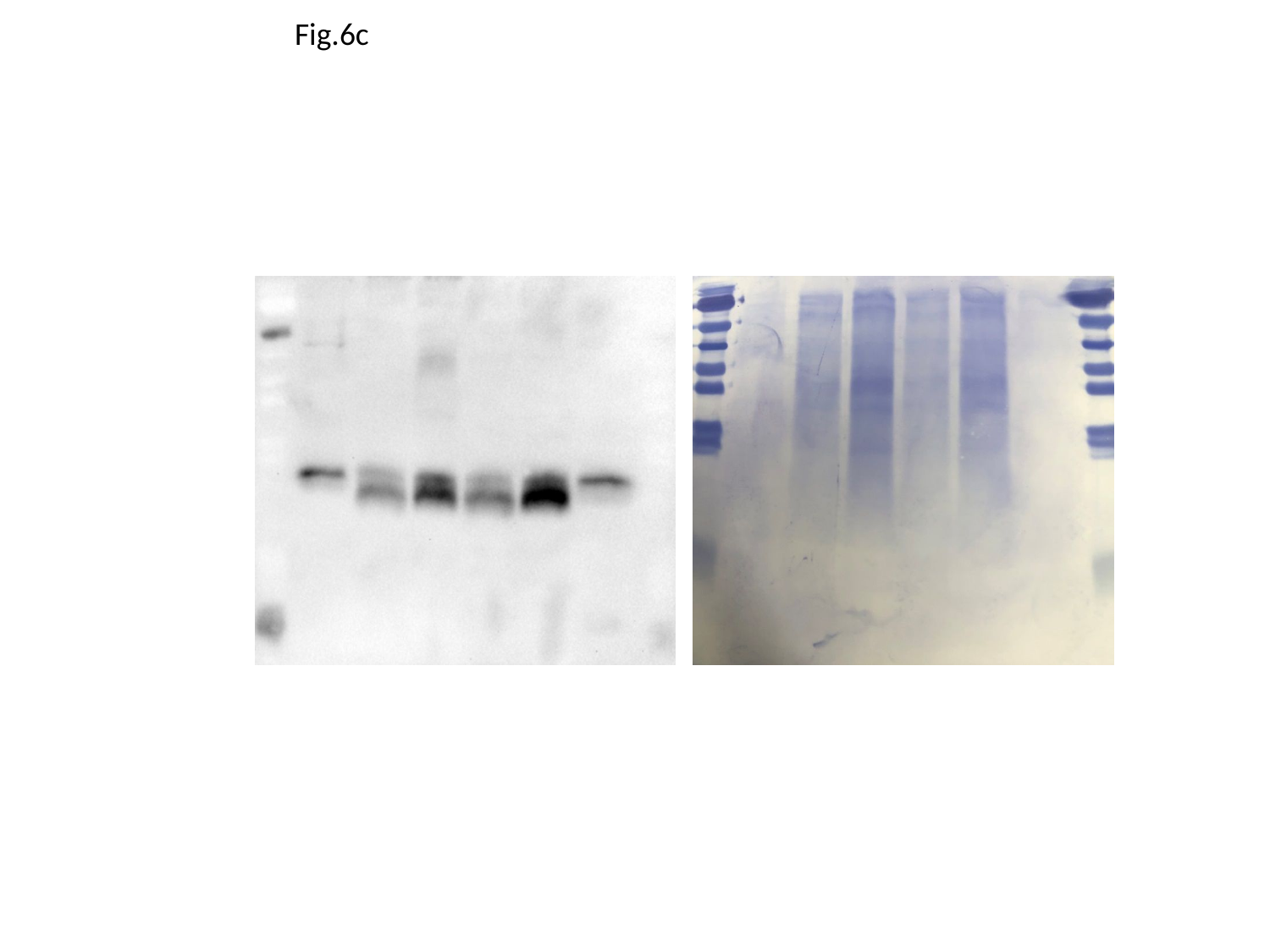

Fig.6c

## Slide 4
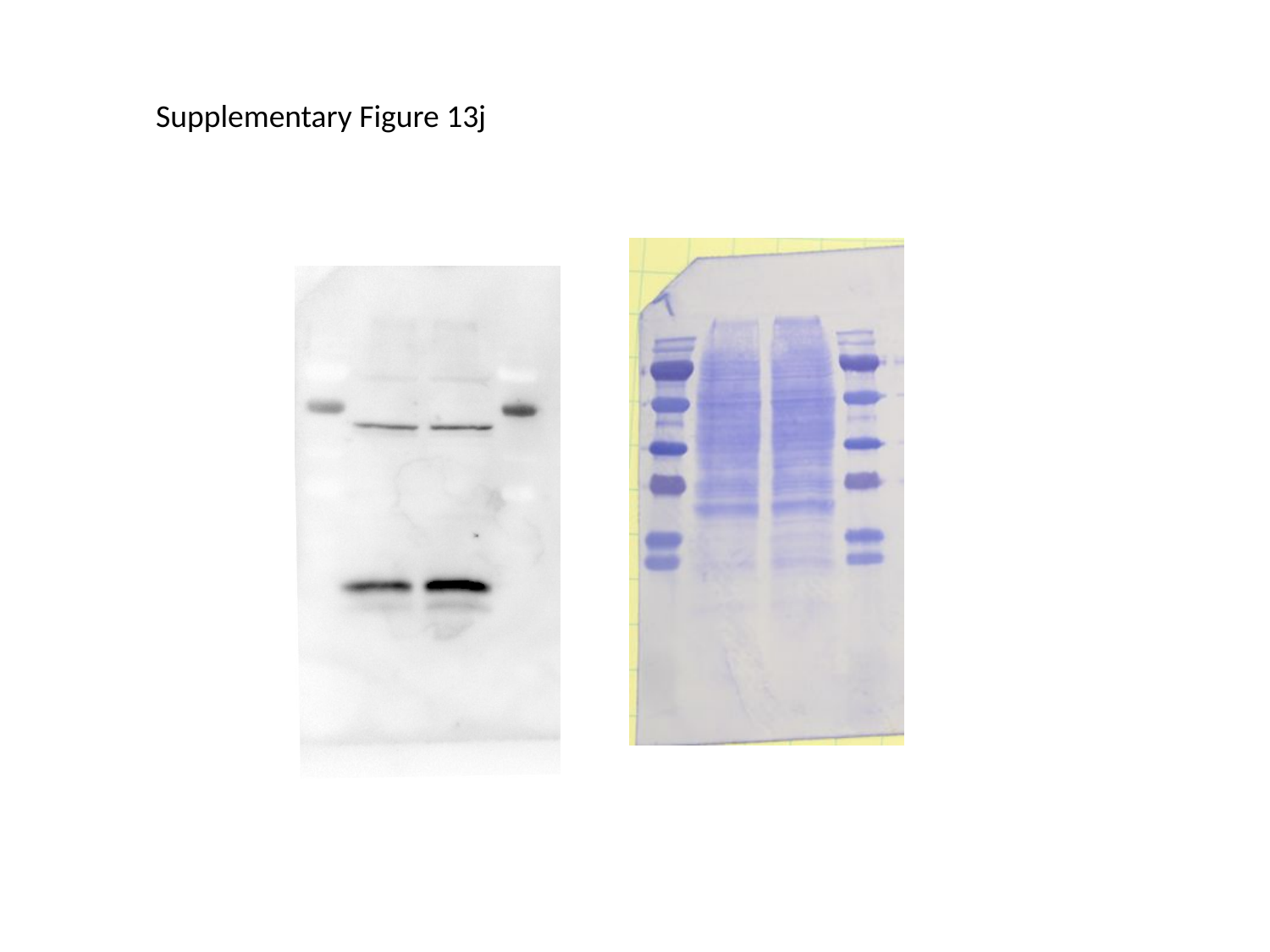

Supplementary Figure 13j

## Slide 5
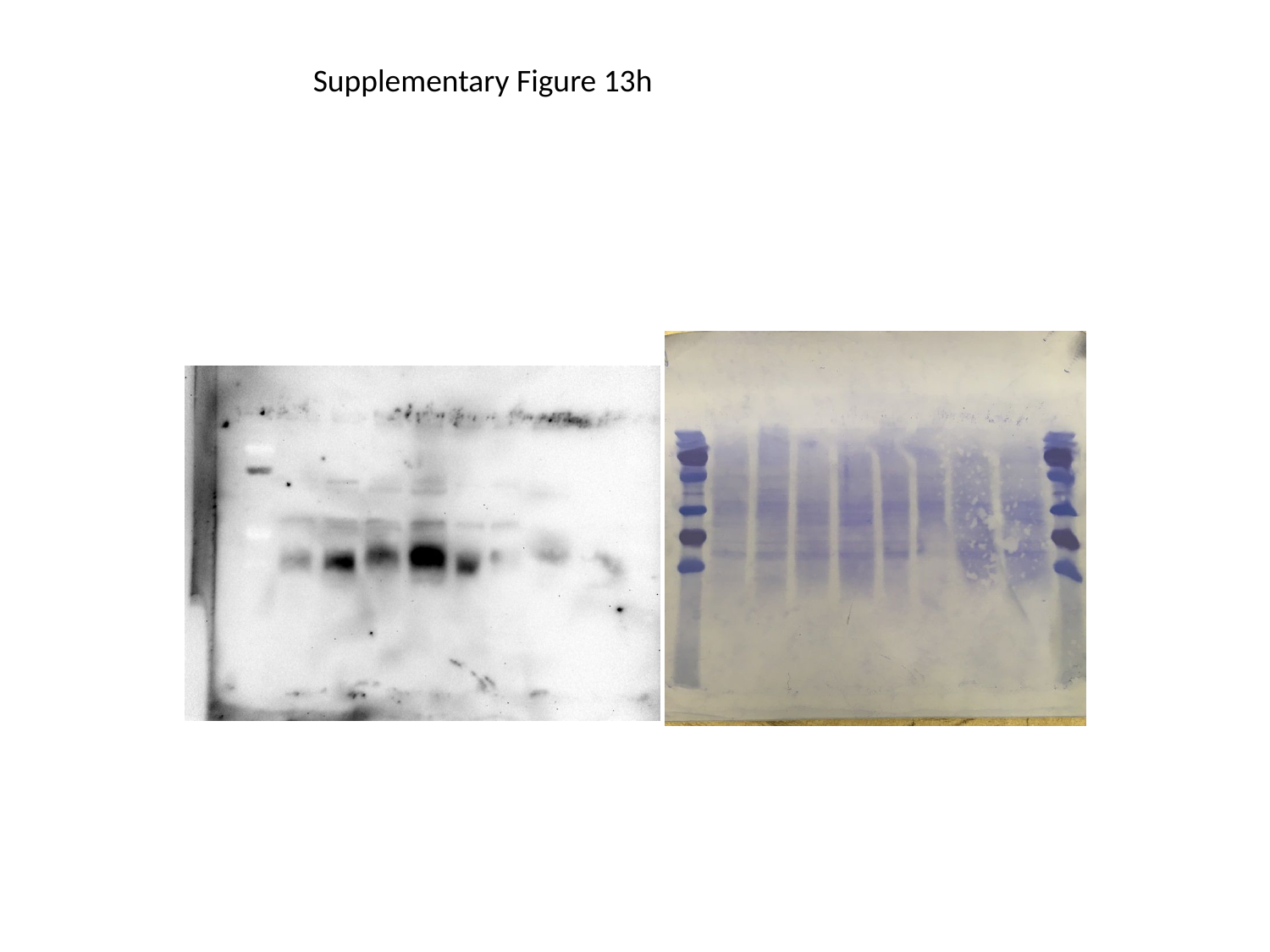

Supplementary Figure 13h

## Slide 6
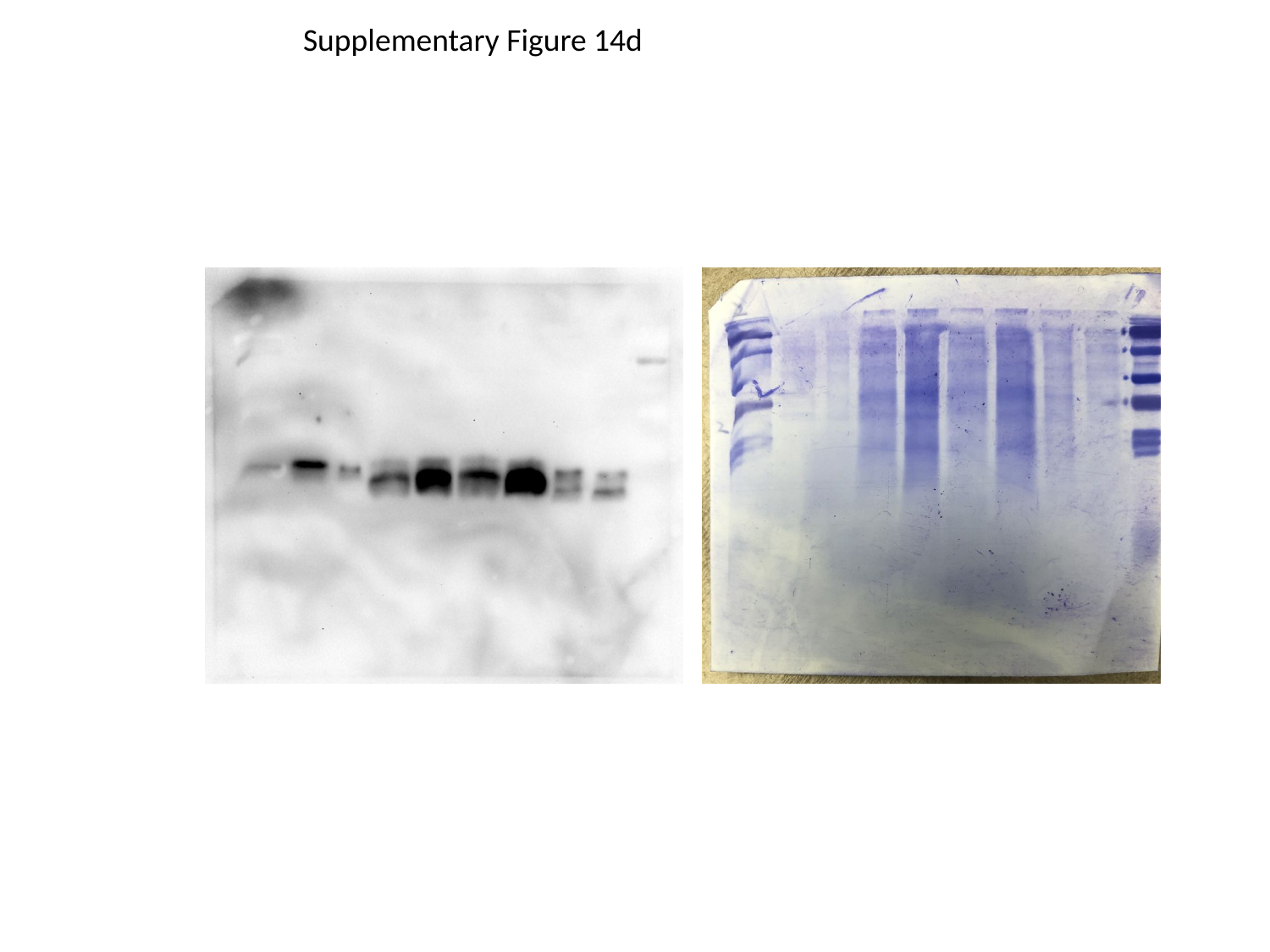

Supplementary Figure 14d
