# Supplementary material for: Genome of Paspalum vaginatum and the role of trehalose mediated autophagy in increasing maize biomass
Source: Nat Commun. 2022 Dec 13;13:7731. doi: 10.1038/s41467-022-35507-8 (PMC9747981; doi:10.1038/s41467-022-35507-8)
Supplement: Supplementary file 2 — Peer Review File [file 41467_2022_35507_MOESM2_ESM.pdf]

nature portfolio

# Peer Review File

## Genome of Paspalum vaginatum and the role of trehalose mediated autophagy in increasing maize biomass

**Open Access** This file is licensed under a Creative Commons Attribution 4.0 International License, which permits use, sharing, adaptation, distribution and reproduction in any medium or format, as long as you give appropriate credit to the original author(s) and the source, provide a link to the Creative Commons license, and indicate if changes were made. In the cases where the authors are anonymous, such as is the case for the reports of anonymous peer reviewers, author attribution should be to 'Anonymous Referee' followed by a clear attribution to the source work. The images or other third party material in this file are included in the article's Creative Commons license, unless indicated otherwise in a credit line to the material. If material is not included in the article's Creative Commons license and your intended use is not permitted by statutory regulation or exceeds the permitted use, you will need to obtain permission directly from the copyright holder. To view a copy of this license, visit http://creativecommons.org/licenses/by/4.0/.

Reviewers' Comments:

Reviewer #1:
Remarks to the Author:
The manuscript "The genome of stress tolerant crop wild relative Paspalum vaginatum leads to increased biomass productivity in the crop Zea mays" by Sun et al. presents genomics, transcriptomics and metabolomics data characterizing a highly abiotic stress tolerant grass, seashore paspalum (Paspalum vaginatum) and its domesticated crop relatives, maize and sorghum to reveal molecular mechanisms underlying paspalum's adaptation to environmental adversity. The results indicated that high nutritional deficiency tolerance in seashore paspalum is associated with significantly increased accumulation of trehalose coinciding with altered expression of the genes involved in trehalose metabolism, which was not observed in maize and sorghum. The similar mechanism uniquely identified in paspalum could also be adopted in the closely related crops for performance enhancement against abiotic stress, i.e., trehalase inhibition by validamycin A (ValA) in nutrition stressed maize and sorghum, the domesticated relatives of paspalum, led to increased trehalose accumulation and significantly reduced biomass loss recapitulating the paspalum phenotype under nitrogen-deficit condition. Trehalose-mediated stress tolerance was demonstrated to be associated with enhanced autophagy in maize seedlings. The research addresses plant-environment interaction, one of the most important factors significantly impacting agriculture production. The research provides new insight into the role of trehalose metabolism and the underlying mechanisms in plant abiotic stress response, which could lead to the development of novel molecular strategies for enhanced food crop performance under adverse environment conditions and therefore should be of general interest to related research community. The experiments were well-designed and the data well presented. I have a couple of questions that need to be addressed:

1. In both normal and nitrogen-deficit condition, ValA treatment of maize plants resulted in decreased ZmTPP (Fig. 5A) and ZmTPS (Fig. 5B) expression. The authors argued that a potential decline in trehalose-6-phosphate accumulation because of the decrease in ZmTPP and ZmTPS expression would lead to increased SNRK1 activity (Fig. 5C) and therefore, enhanced autophagy (Fig. 5E-H) and enhanced/un-affected biomass under normal/nitrogen-deficit condition (Fig. 5I, J). However, why did the expression of both ZmTPP and ZmTPS decline? One would otherwise expect a decrease in ZmTPS expression, but an increase in ZmTPP expression, which would surely result in a declined trehalose-6-phosphate accumulation. Any explanation for this? Can trehalose-6-phosphate content in both control and ValA-treated plants be measured to support your hypothesis?
2. Also, if trehalose-6-phosphate as a signaling molecule indeed triggers SNRK1-mediated autophagy accumulation leading to altered plant stress response (Fig. S11F), does an increase in trehalose accumulation upon ValA treatment (Fig. 4B) have anything to do with this change? what role trehalose itself plays in this process (Fig. 4B)?
3. L 290 - L 293: the authors stated that "A set of 27 genes associated with trehalose metabolism exhibited significantly more rapid rates of protein sequence evolution in paspalum than did the orthologs of these same genes in foxtail millet (S. italica, p = 0.002), sorghum (S. bicolor, p = 0.014) and Oropetium (O. thomaeum, p = 0.025) (Figure 4 E)." This has nothing to do with Fig. 4E. Where are the protein evolution data?
4. L 348 - L 350: the statement "The sequencing of a reference genome for this species allowed us to perform comparative evolutionary analyses, which identified accelerated protein sequence evolution of genes involved in trehalose metabolism in paspalum (Figure 4 J)." has nothing to do with Fig. 4J. Where are the data showing accelerated protein sequence evolution of genes involved in trehalose metabolism in paspalum?
5. Some typing and grammatical errors, for example:

- L 43: delete "were observed"
- L 179: Fig. 3B should be Fig. 3A
- L 230: "where" should be "were"
- L 277: delete one "for"

- L 401: delete “with”

Reviewer #2:
Remarks to the Author:
The paper makes a noble effort at making an impactful science story out of genome sequence of Paspalum vaginatum (a wild relative of maize and sorghum) and growth experiments on the three species. However, right from the title, the claims do not fit the data or make a novel, logical, coherent story.

The title is misleading. The genome of stress tolerant crop wild relative Paspalum vaginatum leads to increased biomass productivity in the crop Zea mays. The genome of Paspalum doesn’t lead to increased biomass productivity in maize. Feeding validamycin, an inhibitor of trehalase leads to increased biomass in maize. The title implies that the genome of Paspalum or part of it has been transferred to maize or is responsible for the growth effects which is not the case.
Genome sequencing of Paspalum vaginatum and comparison with crops is the starting point for the paper. As paspalum is a wild stress-resistant species related to maize and sorghum paspalum, sorghum and maize are grown under N and P deficiency and nutrient sufficiency, with a view that genes/ mechanisms from paspalum could be utilised in improvement of crop stress resistance/ resource use efficiency. Paspalum performs the same in terms of biomass under the three growth conditions whereas the other 2 species have divergent growth in the different nutrient conditions. Maize grows best in all 3 conditions. Metabolites are measured in roots together with transcriptomics. The rational for doing this in roots is not clear. Trehalose stands out as a metabolite that accumulates -N/ full and -P/ full in Paspalum. Trehalase expression is markedly lower in Paspalum and this could allow trehalose to accumulate. Trehalose phosphate synthase (TPS) and trehalose phosphate phosphatase (TPP) genes show altered expression pattern in the 3 species, with difference in the 3 species, some strong induction under low N in palspalum, but all 3 species show some increases in expression under low N. Based on trehalose, TPS, TPP data, experiments are performed to induce trehalose accumulation in maize and sorghum with validamycin A, an inhibitor of trehalase, to phenocopy paspalum. What happens is a stimulation of growth in both maize and sorghum under low N with validamycin A treatment that is attributed to increased autophagy in maize seedlings treated with validamycin A. This is an interesting finding, but there are issues with it that need to be resolved and it is hard to see how this relates back to the starting point of the genome sequence of palspalum.
1.How does stimulation of growth through trehalose accumulation under nutrient deficiency phenocopy paspalum, when palspalum hardly grows at all? A growth switch has been induced, the opposite of what is happening in palspalum.
2.Upregualtion of N metabolism is a more likely explanation than increased autophagy to explain the increased growth at low N by validamycin treatment and trehalose accumulation in maize. The current paper shows upregulation of N transporters (Fig. 5D) and a paper by Lin, Y., Zhang, J., Gao, W. et al. Exogenous trehalose improves growth under limiting nitrogen through upregulation of nitrogen metabolism. BMC Plant Biol 17, 247 (2017). https://doi.org/10.1186/s12870-017-1207-z in Nicotiana tabacum shows stimulation of growth under low N by trehalose application through upregulation of nitrogen metabolism. The current paper refers to this paper briefly but misquotes it by saying that the work was conducted in Nicotiana benthamiana leaves (line 379) when in fact in was whole plants of Nicotiana tabacum performed in quite some depth. The paper is also not the first to show trehalose accumulation under N deficiency as claimed, as Lin et al. (2017) also show increased trehalose under low N. The accumulation of trehalose 6-phosphate under low N in arabidopsis was shown by Nunes et al. 2013 Plant Physiology.
3.Autophagy comes out of the blue somewhat. It is unclear why the authors have gone after this as an explanation to the exclusion of other things. It may be involved in the response to validamycin feeding and trehalose accumulation, but to get such a large effect through validamycin feeding at low N, extra N must be coming from somewhere i.e. through upregulation of N transporters (shown by the authors) and upregulation of N metabolism (shown by Lin et al. 2017). How do these findings relate

back to the genome sequence of palspalum, the starting point for the study? Palspalum doesn't grow much at full nutrition and this may be the more interesting aspect to look at as it could be that a growth switch (activated by T6P/ trehalose) does not happen in paspalum but it does in maize and sorghum because they have been bred to be productive. Can you find evidence for this? How can the genetic sequence data of TPSs and TPPs show evidence for this? Maybe this underpins salt tolerance too, a more likely driver of genome evolution in paspalum. A coherent valid study does not emerge from the current work that would be of value to the scientific community.

Reviewer #3:
Remarks to the Author:
This study starts with the comparison of the genomes Paspalum vaginatum, maize and sorghum with the aim to investigate why P vaginatum is more tolerant to stresses than its maize and sorghum relatives.
Tolerances to nitrogen and phosphate limitations were monitored showing the negative effects on both root and shoot biomass in maize and sorghum, but the absence of any effect on P. vaginatum. Nevertheless, in P. vaginatum, N concentrations was lower under low nitrate than in control condition, and P concentrations were lower under P deficiency compared to control conditions. This showed that decreased N and P resources did not affect plant biomass in P. vaginatum, at least at 21 days after planting.
Then the physiological response to N and P deficits in P. vaginatum was characterized using metabolite profiling and transcriptome analyses in the three species, comparing N or P limited conditions to ideal condition. These two approaches showed that the main difference between P vaginatum on one side and maize and sorghum on the other side, was related to trehalose metabolism. Trehalose relative concentrations in –N and –P were increased and the expression of the TRE1 gene, coding trehalase enzyme involved in trehalose catabolism, was enhanced under stress in P. vaginatum, and unchanged in maize and sorghum. Authors then hypothesized that tolerance of P. vaginatum to N and P stresses was due to trehalose signalling. The inhibition of trehalase activity using the ValA drug recapitulated P. vaginatum phenotype, increasing plant biomass in maize and in lesser extend in sorghum under both ideal and N deficit conditions. Further, authors showed that ValA induced ammonium and nitrate transporters in maize and increased ATG8-PE/ATG8 suggesting increased uptake and autophagic activity.
In order to demonstrate that autophagy was the causal effect of the ValA-induced plant performances, ValA treatment was combined to the 3-MA inhibitor of PI3K, that is commonly used to inhibit autophagy (short term treatments usually).
Although autophagy could in part explain the better growth of maize in response to ValA, there may be other effects of ValA contributing to enhanced plant biomass.

Comments:

Experiments seems to have been performed properly, however it is not clear how many time they have been fully repeated. Several independent assays are needed.

This study proposes the trehalase enzyme as a master player in plant tolerance to low nitrate and phosphate. This is certainly the most interesting point. The discussion of the paper is quite short and mainly focused on the role of trehalose as a signal for autophagy induction. Trehalose signalling may have effects other than on autophagy, that could be considered and discussed. In addition, it may also be discussed whether there are other reported evidence, that trehalose is important for plant response to N or P. Discussion could be enriched.

As said in lines 200 and 303, trehalose signalling mainly involves threhalose-6P. Thre-6P should then be measured here.
Also in lines 151->154, authors measured the expression of starch related genes and deduced from

transcript abundances that starch concentration is increased under low N. Measuring starch content enzymatically and staining tissues with Lugol would be a better demonstration.

Line 319: what is the rationale of the higher expression of ammonium and nitrate transporters in maize under ValA treatment?
If we can understand that increasing N uptake is positive for plant growth, this is true under full N condition, but difficult to consider such an effect in N starvation. Authors must clarify their point.

The abundance of ATG-PE versus ATG8 was used to demonstrate the increase in autophagy activity upon ValA treatment. It is highly complicated to detect ATG8-PE in western blots without performing ultracentrifugation (See Vierstra papers). In addition it is known that blotting with ATG8 antibodies produce several unspecific bands. Then to be convincing authors must:
1- Present in supplemental the entire membrane to show molecular mass markers and unspecific bands
2- Have a negative control: Zmatg12 mutants (Li et al. 2015) in which there is no ATG8 lipidation
3- Show PLD treated extracts in which PE was dissociated in vitro from ATG8

MDC staining is not specific for autophagic bodies or autophagosomes. Authors must use the YFP-ZmATG8 plants produced and published by Vierstra group (Li et al. 2015) to monitor autophagic body abundance. In addition the detection on free YFP (YFP-clivage assay) on western blot may attest changes in autophagy activity.

3-MA by itself may have negative effect on plant growth as it blocks the TOR kinase pathway. Its effect is broader than just inhibiting autophagy, and the effect observed in this study might be autophagy independent.
Effect of MA alone should be presented in figure 5J showing significance between control and 3-MA. This point must be discussed.

To be convincing, the dependence on autophagy of the ValA effects should be demonstrated using autophagy mutant. The question is: do autophagy mutants (Zmatg12 for instance) show enhanced biomass /tolerance to N starvation when treated with ValA under both full or –N /-P conditions. If so is the ValA effect as strong as in wild type.
Then authors must apply ValA treatment on the Zmatg12 autophagy mutants under full, -N and –P conditions and compare biomass changes to that of wild type.

Figure 5K shows that genes of TPS are down regulated under –N in Zmatg12 mutants. What is the rationale of this result? Does it means that autophagy controls trehalose metabolism. This could actually make sense as several studies in Arabidopsis showed that carbon metabolism is globally down regulated in authophagy mutants (see Masclaux-Daubresse etal 2014). This could be confirmed measuring trehalose in Zmatg12 mutants. This point needs better discussion in the text.

Authors suggest that ValA stimulates N remobilization. What is the effect of ValA on the expression of N remobilization genes (cytosolic glutamine synthetases, proteases, autophagy genes)?

In material and method, 3-MA treatment is not explained. It is said in Material and Method that ATG8-PE signal was verified using phospholipase D but it is not shown in results.

Figure 2 and in the text: N or P contents per dry weigh are usually named N and P concentrations.

There are minor typing errors. For example, in Abstract "resulting", line 43 "were observed" (twice)….

Reviewer #4:

Remarks to the Author:
Sun et al report a chromosome scale assembly of Paspalum vaginatum, a wild grass that is related to the Andropogoneae cereals maize and sorghum with tolerance to various abiotic stresses. The authors collected parallel phenotypic, metabolomic, and transcriptomic datasets under P and N stress and identified the accumulation of trehalose as a distinguishing feature in Paspalum that contributed to increased biomass under nutrient limited conditions. The authors used VMA to increase trehalose accumulation in maize and found an autophagy dependent increase in biomass accumulation under nutrient stress. I read this paper with interest and see no major flaws in the methodology, approaches, or experimental designs. Creating a transgenic line of maize with increased trehalose would help validate these findings, but I don't think this is necessary here. I have a few relatively minor comments.

1. VMA can have pleotropic effects in addition to causing an accumulation of trehalose. VMA is used to control various diseases including rise sheath blight and it has also been shown to induce plant defense responses. Was any phytohormone data collected or other metabolite or gene expression data for +- VMA? The autophagy results are really interesting, but I am wondering if you can draw any other conclusions about what is happening physiologically here. I don't see this as a flaw of the study, but I wonder if this could be elaborated on in the discussion.

2. I'm not sure the title of the paper accurately reflects the findings. The role of trehalose in alleviating nutrient stress was not discovered based on findings from the genome, it was from metabolomics data and careful follow up experiments that could have been done independently of the genome sequence. The title makes it seem like some genetic elements from Paspalum were used to increase productivity in maize. I wonder if an alternative title would better reflect the work shown here.

Line 263 This line should reference Supplemental Figure 9.

Line 455. It is unclear how scaffolds orientation and ordering could be verified using sorghum cDNAs. I'm not sure if this is a typo or some unusual approach I am unfamiliar with.

Line 459. I think this note could just be moved to the methods.

Reviewers Text in Black
Final Responses in Blue
Reviewer #1 (Remarks to the Author):

The manuscript “The genome of stress tolerant crop wild relative Paspalum vaginatum leads to increased biomass productivity in the crop Zea mays” by Sun et al. presents genomics, transcriptomics and metabolomics data characterizing a highly abiotic stress tolerant grass, seashore paspalum (Paspalum vaginatum) and its domesticated crop relatives, maize and sorghum to reveal molecular mechanisms underlying paspalum’s adaptation to environmental adversity. The results indicated that high nutritional deficiency tolerance in seashore paspalum is associated with significantly increased accumulation of trehalose coinciding with altered expression of the genes involved in trehalose metabolism, which was not observed in maize and sorghum. The similar mechanism uniquely identified in paspalum could also be adopted in the closely related crops for performance enhancement against abiotic stress, i.e., trehalase inhibition by validamycin A (ValA) in nutrition stressed maize and sorghum, the domesticated relatives of paspalum, led to increased trehalose accumulation and significantly reduced biomass loss recapitulating the paspalum phenotype under nitrogen-deficit condition. Trehalose-mediated stress tolerance was demonstrated to be associated with enhanced autophagy in maize seedlings. The research addresses plant-environment interaction, one of the most important factors significantly impacting agriculture production. The research provides new insight into the role of trehalose metabolism and the underlying mechanisms in plant abiotic stress response, which could lead to the development of novel molecular strategies for enhanced food crop performance under adverse environment conditions and therefore should be of general interest to related research community. The experiments were well-designed and the data well presented. I have a couple of questions that need to be addressed:

1. In both normal and nitrogen-deficit condition, ValA treatment of maize plants resulted in decreased ZmTPP (Fig. 5A) and ZmTPS (Fig. 5B) expression. The authors argued that a potential decline in trehalose-6-phosphate accumulation because of the decrease in ZmTPP and ZmTPS expression would lead to increased SNRK1 activity (Fig. 5C) and therefore, enhanced autophagy (Fig. 5E-H) and enhanced/un-affected biomass under normal/nitrogen-deficit condition (Fig. 5I, J). However, why did the expression of both ZmTPP and ZmTPS decline? One would otherwise expect a decrease in ZmTPS expression, but an increase in ZmTPP expression, which would surely result in a declined trehalose-6-phosphate accumulation. Any explanation for this? Can trehalose-6-phosphate content in both control and ValA-treated plants be measured to support your hypothesis?

We apologize for the confusion caused by the display of expression data in the last version of the manuscript. We do agree that a substantial and consistent decrease in ZmTPS and increase in ZmTPP would be consistent with a decline in

trehalose-6-phosphate content. And indeed both ZmTPP1 and ZmTPS1 exhibited decreased expressed expression in response to ValA treatment.

However, as discussed in the manuscript, in maize both TPS and TPP are encoded by multi-member gene families with different patterns of response to both nitrogen and Val treatments. In this revised manuscript we include a more detailed examination and visualization of expression patterns of all members of the ZmTPP and ZmTPS gene families as Figure S12A. A recent paper in wheat also reported this pattern of genes encoding members of the TPP family exhibiting different and sometimes opposite patterns of expression change in response to environmental stimuli (Du et al, 2022).

Unfortunately we do not have access to facilities that would allow us to accurately quantify changes in the abundance of T6P at the low levels of abundance it is found in out samples. Given this gap in our data, in this revised draft we have de-emphasized our interpretation of T6P based signaling and also discuss potential alternative models including activation of autophagy via SNRK1/AMPK as a result of the inhibition of glucose transporters by trehalose accumulation (DeBosch et al, 2016)  (Line 335-341; 447-452)

2. Also, if trehalose-6-phosphate as a signaling molecule indeed triggers SNRK1-mediated autophagy accumulation leading to altered plant stress response (Fig. S11F), does an increase in trehalose accumulation upon ValA treatment (Fig. 4B) have anything to do with this change? what role trehalose itself plays in this process (Fig. 4B)?

As mentioned more briefly above, in mammalian system, trehalose has been shown to inhibit solute carrier family glucose transporters resulting in the activation of SNKR1/AMPK and thus autophagy.

In this revised manuscript we examined the expression of all four annotated non-chroloplast SLC glucose transporters in maize treated ValA. Three of the four non-plastid SLC glucose transporters were significantly downregulated in maize plants treated with ValA relative to untreated plants (Figure S12F&G). This would be consistent with, but is not strong proof, of a similar mechanism at play in plants . These results and potential interpretations are discussed in the revised manuscript (Line 335-341; 447-452). It is certainly also possible that prolonged trehalose accumulation as a result of ValA treatment produces a decline in T6P but, as discussed, we were unable to quantify T6P one way or another.

3. L 290 - L 293: the authors stated that “A set of 27 genes associated with trehalose metabolism exhibited significantly more rapid rates of protein sequence evolution in paspalum than did the orthologs of these same genes in foxtail millet (S. italica, p =

0.002), sorghum (S. bicolor, p = 0.014) and Oropetium (O. thomaeum, p = 0.025) (Figure 4 E).” This has nothing to do with Fig. 4E. Where are the protein evolution data?

We apologize for the inconsistency in the previous draft. We have added the figure related to protein evolution referred to in the excerpt the reviewer has highlighted as Figure 4G in the revised text. Results are referred and discussed on lines 295-301; 389-391 (previous draft lines 290-293;348-350 ).

4. L 348 - L 350: the statement “The sequencing of a reference genome for this species allowed us to perform comparative evolutionary analyses, which identified accelerated protein sequence evolution of genes involved in trehalose metabolism in paspalum (Figure 4 J).” has nothing to do with Fig. 4J. Where are the data showing accelerated protein sequence evolution of genes involved in trehalose metabolism in paspalum?

This concern is the result of the same error in the previous draft highlighted by the reviewer #1 in comment #3. The protein sequence evolution results referred to as Figure 4J in the previous draft are now included as Figure 4G in our revised draft. Revised text referring to this figure is present on lines 290-293;348-350 of the new draft (290-293 and 348-350 of the previous draft)

5. Some typing and grammatical errors, for example:

- L 43: delete “were observed”
Deleted
- L 179: Fig. 3B should be Fig. 3A
Corrected
- L 230: “where” should be “were”
Corrected
- L 277: delete one “for”
Corrected
- L 401: delete “with”
Corrected

Reviewer #2 (Remarks to the Author):

The paper makes a noble effort at making an impactful science story out of genome sequence of Paspalum vaginatum (a wild relative of maize and sorghum) and growth experiments on the three species. However, right from the title, the claims do not fit the data or make a novel, logical, coherent story.

The title is misleading. The genome of stress tolerant crop wild relative Paspalum vaginatum leads to increased biomass productivity in the crop Zea mays. The genome of Paspalum doesn’t lead to increased biomass productivity in maize. Feeding validamycin,

an inhibitor of trehalase leads to increased biomass in maize. The title implies that the genome of Paspalum or part of it has been transferred to maize or is responsible for the growth effects which is not the case.

In response to reviewer #2's concern -- as well as reviewer #4's below -- we have revised the title of this resubmission to read "Genome assembly and multi-omics of *Paspalum vaginatum* reveal a role for trehalose mediated autophagy in increased biomass productivity of the related crop *Zea mays*"

Genome sequencing of Paspalum vaginatum and comparison with crops is the starting point for the paper. As paspalum is a wild stress-resistant species related to maize and sorghum paspalum, sorghum and maize are grown under N and P deficiency and nutrient sufficiency, with a view that genes/ mechanisms from paspalum could be utilised in improvement of crop stress resistance/ resource use efficiency. Paspalum performs the same in terms of biomass under the three growth conditions whereas the other 2 species have divergent growth in the different nutrient conditions. Maize grows best in all 3 conditions. Metabolites are measured in roots together with transcriptomics. The rational for doing this in roots is not clear.

We apologize for not stating this clearly in the previous version of the manuscript. The greater accumulation of paspalum biomass under nutrient limited conditions (where it accumulated as much biomass under nitrogen limited conditions as sorghum accumulated under nutrient replete conditions) led us to initially hypothesis that paspalum might be superior in nutrient uptake and nutrient scavenging. We therefore designed a set of data collection experiments focused on root tissue. This point is discussed in our revised introduction (Lines 47-49)

Trehalose stands out as a metabolite that accumulates -N/ full and -P/ full in Paspalum. Trehalase expression is markedly lower in Paspalum and this could allow trehalose to accumulate. Trehalose phosphate synthase (TPS) and trehalose phosphate phosphatase (TPP) genes show altered expression pattern in the 3 species, with difference in the 3 species, some strong induction under low N in palspalum, but all 3 species show some increases in expression under low N. Based on trehalose, TPS, TPP data, experiments are performed to induce trehalose accumulation in maize and sorghum with validamycin A, an inhibitor of trehalase, to phenocopy paspalum.

What happens is a stimulation of growth in both maize and sorghum under low N with validamycin A treatment that is attributed to increased autophagy in maize seedlings treated with validamycin A. This is an interesting finding, but there are issues with it that need to be resolved and it is hard to see how this relates back to the starting point of the genome sequence of palspalum.

Thank you for your complimentary words about the interest of our findings.

1.How does stimulation of growth through trehalose accumulation under nutrient deficiency phenocopy paspalum, when palspalum hardly grows at all? A growth switch has been induced, the opposite of what is happening in palspalum.

Upon re-examination of our previous draft we understand how the reviewer came to the conclusion that paspalum plants do not grow.  appear to be smaller because of the higher level of bifurcation and thin stems (Figure 2A) even though paspalum accumulated a similar amount of fresh biomass as sorghum (paspalum median fresh weight accumulation 1.13 g; sorghum median fresh weight accumulation 1.18 g ) over an equivalent length of time after planting (Figure 2B), in addition, we examined the dry weight of the above ground tissue 21 days after planting and we observed two fold more biomass accumulation of paspalum (median 0.19 g) than that of sorghum (median 0.08) under Full condition (Figure 4E & Figure S9G) . We now explicitly discuss the sorghum/paspalum comparison and the potential to be misled by the visual appearance of paspalum ramets and have enlarged the photos to aid visual analysis. (Lines 134-136)

2.Upregualtion of N metabolism is a more likely explanation than increased autophagy to explain the increased growth at low N by validamycin treatment and trehalose accumulation in maize. The current paper shows upregulation of N transporters (Fig. 5D) and a paper by Lin, Y., Zhang, J., Gao, W. et al. Exogenous trehalose improves growth under limiting nitrogen through upregulation of nitrogen metabolism. BMC Plant Biol 17, 247 (2017). https://doi.org/10.1186/s12870-017-1207-z in Nicotiana tabacum shows stimulation of growth under low N by trehalose application through upregulation of nitrogen metabolism. The current paper refers to this paper briefly but misquotes it by saying that the work was conducted in Nicotiana benthamiana leaves (line 379) when in fact it was whole plants of Nicotiana tabacum performed in quite some depth. The paper is also not the first to show trehalose accumulation under N deficiency as claimed, as Lin et al. (2017) also show increased trehalose under low N. The accumulation of trehalose 6-phosphate under low N in arabidopsis was shown by Nunes et al. 2013 Plant Physiology.

The reviewer is correct, and we have removed the statement regarding the novelty of paspalum accumulating trehalose in response to nitrogen deficit stress.

We have re-written the text citing Lin et al in this revised manuscript (line 421 to 423) to make clear that, while trehalose was applied to leaves, data was collected from whole plants.

Based on the feedback from the reviewer we analyzed the expression of genes related to N metabolism, and did indeed observe upregulation, consistent with the reports of Lin et al. (2017). We include these new analyses in Figure 5A&B.

However, based on feedback from reviewer #3, in this revised manuscript we include data from a mutant impaired in autophagy but not in nitrogen metabolism. In this mutant the increased growth observed in wild type plants in response to ValA treatment is abolished (Figure 6). Based on this new data we conclude that, while upregulation of nitrogen metabolism may play some role in increased growth, autophagy function is necessary for the increased growth we observed under low N in response to ValA treatment (Lines 306-309)

3.Autophagy comes out of the blue somewhat. It is unclear why the authors have gone after this as an explanation to the exclusion of other things. It may be involved in the response to validamycin feeding and trehalose accumulation, but to get such a large effect through validamycin feeding at low N, extra N must be coming from somewhere i.e. through upregulation of N transporters (shown by the authors) and upregulation of N metabolism (shown by Lin et al. 2017).
How do these findings relate back to the genome sequence of palspalum, the starting point for the study?

As discussed above, we now include additional analysis of nitrogen metabolism related genes as a potential alternative hypothesis (Lines 309-316, Figure 5A & B) and additional experiments demonstrating that autophagy is indeed necessary for the large effect observed from treating maize plants with ValA under low N conditions (Figure 6, Figure S11).

We have made edits to the narrative that we hope will allow readers to more comfortably follow the progression of this manuscript from comparative growth of two crops and one crop wild relative, to the genome sequencing and annotation of that crop wild relative, to hypothesis generating ‘omics work in the crop wild relative and back to hypothesis testing work in a crop species itself.

Palspalum doesn’t grow much at full nutrition and this may be the more interesting aspect to look at as it could be that a growth switch (activated by T6P/ trehalose) does not happen in paspalum but it does in maize and sorghum because they have been bred to be productive. Can you find evidence for this?

As discussed above, the previous version of our manuscript left the reviewer with a mistaken impression that paspalum ramets didn’t grow under these experimental conditions. Paspalum accumulated as much as sorghum under full nutrient conditions. We have revised the text and figures in ways that we hope will reduce the likelihood of this misunderstanding for future readers.

How can the genetic sequence data of TPSs and TPPs show evidence for this? Maybe this underpins salt tolerance too, a more likely driver of genome evolution in paspalum. A

coherent valid study does not emerge from the current work that would be of value to the scientific community.

In this revised manuscript we now address the potential alternative explanation that increased protein sequence evolution of genes related to trehalose in the paspalum genome may result from selection salt tolerance (lines 290-293;348-350)

Reviewer #3 (Remarks to the Author):

This study starts with the comparison of the genomes Paspalum vaginatum, maize and sorghum with the aim to investigate why P vaginatum is more tolerant to stresses than its maize and sorghum relatives.
Tolerances to nitrogen and phosphate limitations were monitored showing the negative effects on both root and shoot biomass in maize and sorghum, but the absence of any effect on P. vaginatum. Nevertheless, in P. vaginatum, N concentrations was lower under low nitrate than in control condition, and P concentrations were lower under P deficiency compared to control conditions. This showed that decreased N and P resources did not affect plant biomass in P. vaginatum, at least at 21 days after planting.
Then the physiological response to N and P deficits in P. vaginatum was characterized using metabolite profiling and transcriptome analyses in the three species, comparing N or P limited conditions to ideal condition. These two approaches showed that the main difference between P vaginatum on one side and maize and sorghum on the other side, was related to trehalose metabolism. Trehalose relative concentrations in –N and –P were increased and the expression of the TRE1 gene, coding trehalase enzyme involved in trehalose catabolism, was enhanced under stress in P. vaginatum, and unchanged in maize and sorghum. Authors then hypothesized that tolerance of P. vaginatum to N and P stresses was due to trehalose signalling. The inhibition of trehalase activity using the ValA drug recapitulated P. vaginatum phenotype, increasing plant biomass in maize and in lesser extend in sorghum under both ideal and N deficit conditions. Further, authors showed that ValA induced ammonium and nitrate transporters in maize and increased ATG8-PE/ATG8 suggesting increased uptake and autophagic activity.

In order to demonstrate that autophagy was the causal effect of the ValA-induced plant performances, ValA treatment was combined to the 3-MA inhibitor of PI3K, that is commonly used to inhibit autophagy (short term treatments usually).
Although autophagy could in part explain the better growth of maize in response to ValA, there may be other effects of ValA contributing to enhanced plant biomass.

Comments:

Experiments seems to have been performed properly, however it is not clear how many time they have been fully repeated. Several independent assays are needed.

Thank you for pointing out this omission from the previous draft of the manuscript. We have revised our methods section, figure legends, and figure design to to make the extent of replication for different results clear (Legends of Figure2; legends of  Figure 3; legends of Figure4; legends of Figure 5; legends of Figure S9; legends of Figure S10; Methods section line 602-606)

This study proposes the trehalase enzyme as a master player in plant tolerance to low nitrate and phosphate. This is certainly the most interesting point. The discussion of the paper is quite short and mainly focused on the role of trehalose as a signal for autophagy induction. Trehalose signaling may have effects other than on autophagy, that could be considered and discussed. In addition, it may also be discussed whether there are other reported evidence, that trehalose is important for plant response to N or P. Discussion could be enriched.

We substantially expanded the discussion, and cited additional papers on what is known about trehalose in plants under abiotic stress (Lines 410-419) . We then include a paragraph of what is known between the association of trehalose and N/P deficiency (Lines 420-425). In addition we do agree that the application of ValA and the posterior accumulation of trehalose can have an impact on different biological processes within plants. We have expanded the discussion by including some biological processes enriched when ValA is added (Line 428-432), we also have included new analyses of gene expression data from ValA treated and untreated plants to identify other potential biological pathways or processes which may be affected by trehalose signaling (Supplementary Figure 11).

As said in lines 200 and 303, trehalose signalling mainly involves threhalose-6P. Thre-6P should then be measured here.

We regret that the request for direct quantification of Thre-6P was one requested experiment we did not have the capability to provide for this revised manuscript. Precise measurement of Thre-6P requires MS/LC via ion-exchange which can permanently contaminate the MS/LC instrument. Lacking a dedicated MS/LC for Thre-6P quantification we have revised our manuscript to tone down or remove claims and hypotheses related to potential changes in Thre-6P abundance.

Also in lines 151->154, authors measured the expression of starch related genes and deduced from transcript abundances that starch concentration is increased under low N. Measuring starch content enzymatically and staining tissues with Lugol would be a better demonstration.

In this revised manuscript, we have removed our attempt to infer changes in starch abundance from gene expression patterns.

Line 319: what is the rationale of the higher expression of ammonium and nitrate transporters in maize under ValA treatment?
If we can understand that increasing N uptake is positive for plant growth, this is true under full N condition, but difficult to consider such an effect in N starvation. Authors must clarify their point.

While we are unsure whether the response is adaptive or not, it is consistent with reports from the literature of other species under nitrogen starvation (Giehl et al, 2017; Loque et al, 2006), and one could plausibly imagine that in nitrogen constrained but not nitrogen starved conditions upregulation of ammonium and nitrate transporters could allow plants to obtain sufficient N from lower abundance soil substrates. We now discuss this point in the revised manuscript (Lines 313-316)

The abundance of ATG-PE versus ATG8 was used to demonstrate the increase in autophagy activity upon ValA treatment. It is highly complicated to detect ATG8-PE in western blots without performing ultracentrifugation (See Vierstra papers). In addition it is known that blotting with ATG8 antibodies produce several unspecific bands. Then to be convincing authors must:
1- Present in supplemental the entire membrane to show molecular mass markers and unspecific bands
2- Have a negative control: Zmatg12 mutants (Li et al. 2015) in which there is no ATG8 lipidation
3- Show PLD treated extracts in which PE was dissociated in vitro from ATG8

In this revised manuscript we present new western blots from additional experiments that include ultracentrifugation to isolate membranes, cellular solute controls, and PLD treated extracts to confirm the identity of the ATG8-PE band in WT and atg12-2 plants (Figures 6C & S13C). In addition we include an entire membrane, including molecular mass markers as our new figure 13E.

MDC staining is not specific for autophagic bodies or autophagosomes. Authors must use the YFP-ZmATG8 plants produced and published by Vierstra group (Li et al. 2015) to monitor autophagic body abundance. In addition the detection on free YFP (YFP-clivage assay) on western blot may attest changes in autophagy activity.

After receiving the reviewer comments on this manuscript we made multiple attempts to obtain the YFP-ZmATG8 line published by the Vierstra group in Li et al 2015, including traveling in person to St. Louis. We were unsuccessful.

We agree that MDC staining can detect signals other than autophagic bodies. Given our inability to obtain the published Li et al 2015 YFP-ZmATG8 line and the concern expressed above by reviewer #3, we have removed the data resulting from MDC staining from our revised manuscript.

3-MA by itself may have negative effect on plant growth as it blocks the TOR kinase pathway. Its effect is broader than just inhibiting autophagy, and the effect observed in this study might be autophagy independent.
Effect of MA alone should be presented in figure 5J showing significance between control and 3-MA. This point must be discussed.

In this revised manuscript we include the requested statistical test in Figure 6 and discuss (line 473-476).

To be convincing, the dependence on autophagy of the ValA effects should be demonstrated using autophagy mutant. The question is: do autophagy mutants (Zmatg12 for instance) show enhanced biomass /tolerance to N starvation when treated with ValA under both full or –N /-P conditions. If so is the ValA effect as strong as in wild type.
Then authors must apply ValA treatment on the Zmatg12 autophagy mutants under full, -N and –P conditions and compare biomass changes to that of wild type.

As requested by reviewer #3, we first obtained and then increased seed of the ZmATG12-2 mutant (in a W22 genetic background) and then used this seed to perform triplicated experiments comparing mutant and wild type plants. Mutant and wild type plants were evaluated under full and -N conditions and with and without ValA treatments. The results obtained were consistent with the increased biomass accumulation produced by ValA treatment in maize being dependent on autophagy function. New results related to ZmATG12-2 are shown in Figure 6 and Figure S13.

Figure 5K shows that genes of TPS are down regulated under –N in Zmatg12 mutants. What is the rationale of this result? Does it means that autophagy controls trehalose metabolism. This could actually make sense as several studies in Arabidopsis showed that carbon metabolism is globally down regulated in authophagy mutants (see Masclaux-Daubresse etal 2014). This could be confirmed measuring trehalose in Zmatg12 mutants. This point needs better discussion in the text.

As requested by reviewer #3 we quantified trehalose in both wild type W22 and the ZmATG12-2 mutant line and observed an overall lower trehalose abundance in ZmATG12-2 seedlings which indicate carbon metabolism is globally repressed in autophagy mutants (Figure 6B). We discuss these new results on lines (371-378).

Authors suggest that ValA stimulates N remobilization. What is the effect of ValA on the expression of N remobilization genes (cytosolic glutamine synthetases, proteases, autophagy genes)?

As requested by reviewer #3, we examined the expression change of cytosolic glutamine syntheaase genes in response to ValA treatment, Of the five cytosolic glutamine synthetase genes, four (excluding gln2) exhibited increased expression in response to ValA treatment

under full nutrient conditions. Under N starvation only  GLN3 and GLN6 exhibited increased expression in response to ValA treatment. The expression of gln1, which is localized to the chloroplast, decreases in response to ValA treatment, as is gln2 (see Figure 5B in this revised manuscript).  In addition, we also observed an overall upregulation of autophagy related genes and ATG8 interacting genes (see Figure 5C in this revised manuscript).

In material and method, 3-MA treatment is not explained. It is said in Material and Method that ATG8-PE signal was verified using phospholipase D but it is not shown in results.

Thank you for catching these inconsistencies. We have added details of our 3-MA treatment to methods (Lines 601-602), and in this revised manuscript include a western blot showing the PLD control and cellular solute fraction after ultra-centrifugation for the identity of the ATG8-PE signal (Figure 6, Figure S13)

Figure 2 and in the text: N or P contents per dry weigh are usually named N and P concentrations.

We now define N (or P) concentration as N (or P) content per dry weight at first usage and then subsequently use the term N (or P) concentration.

There are minor typing errors. For example, in Abstract "resulting", line 43 "were observed" (twice)….

Thank you for catching this, fixed. We also tried to minimize this type of error by carefully reading the manuscript several times.

Reviewer #4 (Remarks to the Author):

Sun et al report a chromosome scale assembly of Paspalum vaginatum, a wild grass that is related to the Andropogoneae cereals maize and sorghum with tolerance to various abiotic stresses. The authors collected parallel phenotypic, metabolomic, and transcriptomic datasets under P and N stress and identified the accumulation of trehalose as a distinguishing feature in Paspalum that contributed to increased biomass under nutrient limited conditions. The authors used VMA to increase trehalose accumulation in maize and found an autophagy dependent increase in biomass accumulation under nutrient stress. I read this paper with interest and see no major flaws in the methodology, approaches, or experimental designs. Creating a transgenic line of maize with increased trehalose would help validate these findings, but I don't think this is necessary here. I have a few relatively minor comments.

Thank you.

1. VMA can have pleotropic effects in addition to causing an accumulation of trehalose. VMA is used to control various diseases including rise sheath blight and it has also been

shown to induce plant defense responses. Was any phytohormone data collected or other metabolite or gene expression data for +- VMA? The autophagy results are really interesting, but I am wondering if you can draw any other conclusions about what is happening physiologically here. I don't see this as a flaw of the study, but I wonder if this could be elaborated on in the discussion.

Unfortunately we did not collect any phytohomorone data in the present study, but, as you suggest, we now discuss the other effects of VMA in a revised and expanded discussion section.

2. I'm not sure the title of the paper accurately reflects the findings. The role of trehalose in alleviating nutrient stress was not discovered based on findings from the genome, it was from metabolomics data and careful follow up experiments that could have been done independently of the genome sequence. The title makes it seem like some genetic elements from Paspalum were used to increase productivity in maize. I wonder if an alternative title would better reflect the work shown here.

Thank you for pointing this out. Concerns about the title were also brought up by reviewer 2. In this revised manuscript we have changed the title to "The genome and multi-omics of Paspalum vaginatum reveal a role of trehalose mediated autophagy in biomass productivity in its crop relative Zea mays",

Line 263 This line should reference Supplemental Figure 9.
Corrected

Line 455. It is unclear how scaffolds orientation and ordering could be verified using sorghum cDNAs. I'm not sure if this is a typo or some unusual approach I am unfamiliar with.

We apologize for not stating this part clearly and thank you for pointing it out. We have revised the relevant section in methods. Briefly synteny was identified between the paspalum and sorghum genomes by first aligning annotated sorghum transcripts (not cDNAs) to the paspalum genome, and then identifying blocks of collinear alignments spanning multiple annotated sorghum genes between the two species. We have made changes to the sentence in the revised manuscript from line 551 to line 553.

Line 459. I think this note could just be moved to the methods.
Thank you for the suggestion, we have moved it to the methods section.

Reviewers' Comments:

Reviewer #1:
Remarks to the Author:
The authors have appropriately addressed my questions and I would recommend acceptance of the revised manuscript.

Reviewer #2:
Remarks to the Author:
Reviewer #2 (Remarks to the Author, first review): The paper makes a noble effort at making an impactful science story out of genome sequence of Paspalum vaginatum (a wild relative of maize and sorghum) and growth experiments on the three species. However, right from the title, the claims do not fit the data or make a novel, logical, coherent story. The title is misleading. The genome of stress tolerant crop wild relative Paspalum vaginatum leads to increased biomass productivity in the crop Zea mays. The genome of Paspalum doesn't lead to increased biomass productivity in maize. Feeding validamycin, an inhibitor of trehalase leads to increased biomass in maize. The title implies that the genome of Paspalum or part of it has been transferred to maize or is responsible for the growth effects which is not the case.

Authors' response. In response to reviewer #2's concern -- as well as reviewer #4's below -- we have revised the title of this resubmission to read "Genome assembly and multi-omics of Paspalum vaginatum reveal a role for trehalose mediated autophagy in increased biomass productivity of the related crop Zea mays"

Reviewer further comment (review 2). The title "Genome assembly and multiomics of Paspalum vaginatum reveal a role for trehalose mediated autophagy in increased biomass productivity of the related crop Zea mays" is still misleading because mulitomics of paspalum does not infer anything about autophagy. Autophagy is dealt with as a mechanism in maize not from multiomics of paspalum. It needs to be shown that autophagy is a trehalose-dependent mechanism in paspalum for the title to be correct. This isn't shown. In the final paragraph of the Discussion, the authors state that they wouldn't have tried the experiments in maize and sorghum with validamycin to induce trehalose if they hadn't seen trehalose accumulate in pasaplum, but the results of the validamycin experiments could be a completely different story to nutrient deficient growth of paspalum. Any amount of writing to try and connect the two and help the reader doesn't change the facts that the evidence still isn't there to connect the two. So the story is not coherent with not enough evidence to support it. The authors say that it is not practical to overexpress trehalase in paspalum the final paragraph of the discussion (I agree) yet they could look at evidence for an autophagy mechanism in paspalum from their genomic assembly and other data. A coherent story still does not emerge. There are two or three stories that are not well bridged. The paper starts from understanding tolerance of low nutrients in paspalum with a view to transferring this knowledge to crops. Paspalum does well in low P and low N compared to sorghum (but not maize), although this is from one growth time point only. Trehalose accumulates in paspalum but not maize and sorghum and this is proposed as a reason for the better performance of paspalum under nutrient deficient conditions. Gene expression data for the pathway in all 3 species could mean anything, despite claims to the contrary (e.g. line 42 introduction) that genes for the pathway change in paspalum only. They change in all 3 species in both directions according to Fig. S7 and FigS8, where some genes go down in paspalum under nutrient deficiency (why are such important data in the supplementary?). Validamycin is used to treat plants. This causes trehalose accumulation in maize and sorghum which grow better under full and depleted nutrition with autophagy implicated as a mechanism. However, better growth performance under both full and nutrient deficient conditions implicates an interesting role for autophagy in maize and sorghum but does not have anything to say on what is happening in paspalum or relate back to the opening questions. The lack of an effect of validamycin in paspalum does not meant that autophagy as a

mechanism in paspalum accounts for its better performance under low nutrients. Genome assembly in paspalum so far does not reveal anything about trehalose-mediated autophagy. How can superior growth under full nutrition in maize and sorghum with validamycin prove a mechanism of autophagy in paspalum? You need to show that trehalose and autophagy are the mechanism in paspalum then phenocopy in maize. This would then follow logically. What is the trehalose/ autophagy mechanism in paspalum that makes it more tolerant of nutrient deficiency than maize and sorghum? Then induce this mechanism in maize/ sorghum. Surely the starting point is to understand how paspalum is more nutrient use efficient than crops. We don't have this answer. This is how the abstract and Introduction start and is the justification of the study. We don't know the nutrient deficiency tolerance mechanism in paspalum. You must be able to look at autophagy genes in the genomic information you have from paspalum to see what is happening there?
I am concerned that statements in the last paragraph of the Introduction are misleading. First the one about expression of trehalose metabolism genes being observed in paspalum and not in sorghum and maize (Fig. S7 and Fig. S8). This is not true as genes go up and down in all three species and the pattern of changes is difficult to interpret. Then the next sentence starting "Replicating the pattern of trehalose metabolism… does not make sense. From what I think this sentence is meant to say about phenocopying paspalum with validamycin A is not true as the autophagy mechanism in paspalum is not known.
The final paragraph of the Discussion states that an increase in autophagy by the increased accumulation of trehalose in maize is likely to explain the low degree of phenotypic plasticity in paspalum line 491. Much more evidence is required for this. Increased growth under full and low nutrition are observed in maize, this does not phenocopy lack of phenotypic plasticity in paspalum. Additionally in the final paragraph of the Discussion, beginning line 494, it is already well documented that manipulation of the trehalose pathway (to increase trehalose or T6P) through chemical or genetic approach could improve yield per unit of nitrate or phosphate in maize and sorghum and indeed in other plants and crops. Yield improvements have been achieved already without extra N and P fertiliser hence increasing nutrient use efficiency.
Overall the Discussion speculates rather than backs claims with strong evidence.
There are potentially three strands from which papers could emerge which are currently not coherently connected. 1) genome sequencing of paspalum. 2) nutrient use efficiency mechanisms and how paspalum is more nutrient use efficient than maize and sorghum. If it is trehalose, then how does this operate? If it is autophagy then please show this through your analysis in paspalum. 3) Effects in maize with validamycin which may or may not be linked to observations and mechanism in paspalum. Validamycin increases growth in full nutrition in maize not just under nutrient deficiency. Validamycin induced trehalose accumulation does not explain lack of phenotypic plasticity in paspalum.

Reviewer first review: Genome sequencing of Paspalum vaginatum and comparison with crops is the starting point for the paper. As paspalum is a wild stress-resistant species related to maize and sorghum paspalum, sorghum and maize are grown under N and P deficiency and nutrient sufficiency, with a view that genes/ mechanisms from paspalum could be utilised in improvement of crop stress resistance/ resource use efficiency. Paspalum performs the same in terms of biomass under the three growth conditions whereas the other 2 species have divergent growth in the different nutrient conditions. Maize grows best in all 3 conditions. Metabolites are measured in roots together with transcriptomics. The rational for doing this in roots is not clear.

Author response: We apologize for not stating this clearly in the previous version of the manuscript. The greater accumulation of paspalum biomass under nutrient limited conditions (where it accumulated as much biomass under nitrogen limited conditions as sorghum accumulated under nutrient replete conditions) led us to initially hypothesis that paspalum might be superior in nutrient uptake and nutrient scavenging. We therefore designed a set of data collection experiments focused on root tissue. This point is discussed in our revised introduction (Lines 47-49)

Reviewer first review: Trehalose stands out as a metabolite that accumulates -N/ full and -P/ full in Paspalum. Trehalase expression is markedly lower in Paspalum and this could allow trehalose to

accumulate. Trehalose phosphate synthase (TPS) and trehalose phosphate phosphatase (TPP) genes show altered expression pattern in the 3 species, with difference in the 3 species, some strong induction under low N in palspalum, but all 3 species show some increases in expression under low N. Based on trehalose, TPS, TPP data, experiments are performed to induce trehalose accumulation in maize and sorghum with validamycin A, an inhibitor of trehalase, to phenocopy paspalum.

Reviewer further comment (second review). My point here, which has not been answered, is that expression of TPS and TPP in the 3 species does not confirm or deny a role for the pathway in nutrient deficiency (Fig. S7 and Fig. S8). The gene expression data could mean anything. Genes change and go up and down in all species (not what is stated in the paper e.g. line 43).

Reviewer first review: What happens is a stimulation of growth in both maize and sorghum under low N with validamycin A treatment that is attributed to increased autophagy in maize seedlings treated with validamycin A. This is an interesting finding, but there are issues with it that need to be resolved and it is hard to see how this relates back to the starting point of the genome sequence of palspalum.

Author response: Thank you for your complimentary words about the interest of our findings.

Reviewer further comment (second review). The data are interesting but need much more follow up with evidence before they can be published.

Reviewer first review: 1.How does stimulation of growth through trehalose accumulation under nutrient deficiency phenocopy paspalum, when palspalum hardly grows at all? A growth switch has been induced, the opposite of what is happening in palspalum.

Author response: Upon re-examination of our previous draft we understand how the reviewer came to the conclusion that paspalum plants do not grow. appear to be smaller because of the higher level of bifurcation and thin stems (Figure 2A) even though paspalum accumulated a similar amount of fresh biomass as sorghum (paspalum median fresh weight accumulation 1.13 g; sorghum median fresh weight accumulation 1.18 g ) over an equivalent length of time after planting (Figure 2B), in addition, we examined the dry weight of the above ground tissue 21 days after planting and we observed two fold more biomass accumulation of paspalum (median 0.19 g) than that of sorghum (median 0.08) under Full condition (Figure 4E & Figure S9G) . We now explicitly discuss the sorghum/paspalum comparison and the potential to be misled by the visual appearance of paspalum ramets and have enlarged the photos to aid visual analysis. (Lines 134-136)

Reviewer further comment (review 2). The pictures are still too small to see and biomass is presented at 21 days only. To make valid claims on growth more than one timepoint would normally be expected as standard practice in any paper about growth and nutrition.

Reviewer first review> 2.Upregualtion of N metabolism is a more likely explanation than increased autophagy to explain the increased growth at low N by validamycin treatment and trehalose accumulation in maize. The current paper shows upregulation of N transporters (Fig. 5D) and a paper by Lin, Y., Zhang, J., Gao, W. et al. Exogenous trehalose improves growth under limiting nitrogen through upregulation of nitrogen metabolism. BMC Plant Biol 17, 247 (2017). https://doi.org/10.1186/s12870-017-1207-z in Nicotiana tabacum shows stimulation of growth under low N by trehalose application through upregulation of nitrogen metabolism. The current paper refers to this paper briefly but misquotes it by saying that the work was conducted in Nicotiana benthamiana leaves (line 379) when in fact it was whole plants of Nicotiana tabacum performed in quite some depth. The paper is also not the first to show trehalose accumulation under N deficiency as claimed, as Lin et al. (2017) also show increased trehalose under low N. The accumulation of trehalose 6-phosphate under low N in arabidopsis was shown by Nunes et al. 2013 Plant Physiology.

Author response: The reviewer is correct, and we have removed the statement regarding the novelty

of paspalum accumulating trehalose in response to nitrogen deficit stress.
We have re-written the text citing Lin et al in this revised manuscript (line 421 to 423) to make clear that, while trehalose was applied to leaves, data was collected from whole plants.
Based on the feedback from the reviewer we analyzed the expression of genes related to N metabolism, and did indeed observe upregulation, consistent with the reports of Lin et al. (2017). We include these new analyses in Figure 5A&B.
However, based on feedback from reviewer #3, in this revised manuscript we include data from a mutant impaired in autophagy but not in nitrogen metabolism. In this mutant the increased growth observed in wild type plants in response to ValA treatment is abolished (Figure 6). Based on this new data we conclude that, while upregulation of nitrogen metabolism may play some role in increased growth, autophagy function is necessary for the increased growth we observed under low N in response to ValA treatment (Lines 306-309)

Reviewer first review. 3.Autophagy comes out of the blue somewhat. It is unclear why the authors have gone after this as an explanation to the exclusion of other things. It may be involved in the response to validamycin feeding and trehalose accumulation, but to get such a large effect through validamycin feeding at low N, extra N must be coming from somewhere i.e. through upregulation of N transporters (shown by the authors) and upregulation of N metabolism (shown by Lin et al. 2017). How do these findings relate back to the genome sequence of paspalum, the starting point for the study?

Author response: As discussed above, we now include additional analysis of nitrogen metabolism related genes as a potential alternative hypothesis (Lines 309-316, Figure 5A & B) and additional experiments demonstrating that autophagy is indeed necessary for the large effect observed from treating maize plants with ValA under low N conditions (Figure 6, Figure S11).
We have made edits to the narrative that we hope will allow readers to more comfortably follow the progression of this manuscript from comparative growth of two crops and one crop wild relative, to the genome sequencing and annotation of that crop wild relative, to hypothesis generating ‘omics work in the crop wild relative and back to hypothesis testing work in a crop species itself.

Reviewer further comment (review 2). You don’t answer about the role of trehalose and autophagy in paspalum which is necessary to make the link between these two strands in the paper.

Reviewer first review: Palspalum doesn’t grow much at full nutrition and this may be the more interesting aspect to look at as it could be that a growth switch (activated by T6P/ trehalose) does not happen in paspalum but it does in maize and sorghum because they have been bred to be productive. Can you find evidence for this?

Author response: As discussed above, the previous version of our manuscript left the reviewer with a mistaken impression that paspalum ramets didn’t grow under these experimental conditions. Paspalum accumulated as much as sorghum under full nutrient conditions. We have revised the text and figures in ways that we hope will reduce the likelihood of this misunderstanding for future readers.

Reviewer further comment. I don’t say that paspalum doesn’t grow I say that it grows not much under full nutrition and indeed the same under all 3 conditions which is the striking thing. Lack of phenotypic plasticity in paspalum is not explained by the data in the paper.

Reviewer first review: How can the genetic sequence data of TPSs and TPPs show evidence for this? Maybe this underpins salt tolerance too, a more likely driver of genome evolution in paspalum. A coherent valid study does not emerge from the current work that would be of value to the scientific community.

Author response: In this revised manuscript we now address the potential alternative explanation that increased protein sequence evolution of genes related to trehalose in the paspalum genome may

result from selection salt tolerance (lines 290-293;348-350

Reviewer further comment (review 2). Selection for salt tolerance is a possibility certainly but you do not have strong evidence that the trehalose pathway is definitively linked to either low N, low P or to salt tolerance in paspalum.
Overall the evidence for the conclusions and claims made in this paper are not strong enough. You have very interesting leads. Better to consolidate your leads (potentially in more than one paper) with solid data to support your conclusions.

According to criteria for this journal
Quality of data. This is good (but data do not support the conclusions made).
Support for conclusions. Much more evidence is required to support the conclusions made in this paper.
Significance of results. Trehalose pathway modification to improve yield in crops including maize and sorghum per unit of nutrition i.e. with no extra fertiliser is already known about and published.
What would be significant is 1) understanding a mechanism in paspalum that gives it less phenotypic plasticity than other species as would understanding how paspalum is more nutrient use efficient than other species. 2) Pursuing the autophagy mechanism in maize and/ or sorghum as a main focus. Trehalose x autophagy has not been implicated as a mechanism of crop yield improvement. If this can be linked back to paspalum, then even better.

Reviewer #3:
Remarks to the Author:
The manuscript has been significantly improved.
New experiments have been performed that strengthen the demonstration of the involvement of autophagy.
It is a pity that authors could not obtain YFP-ATG8 maize lines to better monitor autophagy activity. Nevertheless, they performed new western blots using ATG8 antibodies on plant extracts treated or not with PLD. The better identification of the ATG8 and ATG8-PE forms is convincing and show the increase of autophagy activity under ValA treatment. The measurement of the expression of autophagy genes is in line, and also shows that autophagy is enhanced in ValA treated plants.
The ValA treatments performed on WT and atg12 maize mutants provides an additional demonstration that the increase of biomass after ValA treatment is autophagy dependent.
Discussion has been significantly improved as suggested.

Minor comment: there are still typing errors in the text.
For example, lines 305-306: no verb in the sentence.

Reviewer #4:
Remarks to the Author:
The authors have addressed my previous comments/concerns in their detailed revision.

Reviewers' Comments:

Reviewer #2:
Remarks to the Author:
• What are the noteworthy results?
-Genome assembly of a halophytic grass species, paspalum (new). Genome assembly is of course a good achievement, but for Nature Communications I think that something extra needs to be found that makes the study noteworthy. I find the follow up to the genome assembly does not provide information that goes beyond speculation of possibilities. The final data in maize on an autophagy mechanism are interesting but do not link back at all well with the genome assembly information in paspalum. It is not clear at all whether the results in maize are applicable to crop improvement. Maize and other cereals have already been targeted successfully through interventions in the trehalose pathway. It is difficult to gain the take home message. For Nature Communications I would expect a major noteworthy finding. There is lots of suggestive and speculative data requiring further evidence and follow up.
- Genes involved in telomere maintenance and DNA repair have copy number expansion in paspalum as do gene families involved in N and P deficiency (interesting but not especially noteworthy)
-Accumulation of trehalose in paspalum under N deficiency (new information for paspalum but found in other spp. too e.g. tobacco Lin et al. 2017 doi: 10.1186/s12870-017-1207-z; T6P accumulation under low N in Arabidopsis Nunes et al. 2013 10.1104/pp.113.220657). Trehalase expression is the only gene expression change in the trehalose pathway that can really explain trehalose accumulation in paspalum. More rapid rate of protein sequence evolution for trehalose pathway in paspalum is new, but trehalose pathway may be an adaption to the environment in which paspalum found e.g. salinity. No causal link with nutrient deficiency shown. Rapid evolution may or may not relate to accumulation of trehalose under N deficiency. Transcription of genes associated with N and P deficiency are more likely explanations for traits related to N and P deficiency and potential enhanced nutrient use efficiency.
-Validamycin treatment of maize gives rise to elevated trehalose and greater growth under low N through autophagy but also altered N metabolism (as previously shown in tobacco Li et al. 2019 10.1111/pbi.129910 but this does not phenocopy paspalum as claimed. Yields per unit of fertiliser have already been increased through targeting trehalose in maize (Nuccio et al. 2015 10.1038/nbt.3277) and abiotic stress resilience generally in other crops, see below.

• Will the work be of significance to the field and related fields? How does it compare to the established literature? If the work is not original, please provide relevant references.
-No substantial originality in the work apart from the genome assembly of paspalum. Autophagy findings in maize interesting but may not be related to data in paspalum. Links of trehalose pathway to paspalum phenotype not substantiated.
-Maize and sorghum already improved through trehalose pathway (Nuccio et al. 2015 10.1038/nbt.3277; Li et al. 2019 10.1111/pbi.12991). Hence novelty for crops not clear. Many cases of trehalose being modified for crop improvement including more yield per unit of fertiliser and water (Garg et al. 2002 10.1073/pnas.252637799 Trehalose accumulation in rice plants confers high tolerance levels to different abiotic stresses) as just one further example.

• Does the work support the conclusions and claims, or is additional evidence needed?

-Current conclusions are all speculative requiring further substantiation. This is not a definitive study apart from providing genome assembly of paspalum.
-Genome assembly of paspalum, then a series of observations with not well demonstrated causal links. Speculation. Over interpretation of data.
-Validamycin treatment of maize phenocopies paspalum only with respect to trehalose accumulation, nothing else. Other maize responses do not phenocopy paspalum. More rapid growth is elicited in maize by validA induced trehalose accumulation (opposite of paspalum). Good evidence for autophagy involved in this response in maize is obtained also nitrate transporters and N metabolism (N

metabolism upregulation previously found in tobacco when treated with trehalose (Lin et al. 2017), but none of this links back to paspalum. Autophagy not shown in paspalum. Even if autophagy were shown in paspalum how does this explain lack of phenotypic plasticity in paspalum given the exaggerated phenotypic plasticity in maize with trehalose accumulation?

• Are there any flaws in the data analysis, interpretation and conclusions? Do these prohibit publication or require revision?
The paper over interprets preliminary observations. There are no sound conclusions apart from a likely role for autophagy in maize responses to trehalose. These data in maize do not link back to paspalum. Hence the work is not well connected and does not form a coherent whole. The Discussion is entirely suggestive and speculative. How would you convince biotech companies to fund this in crops?

• Is the methodology sound? Does the work meet the expected standards in your field?
• Is there enough detail provided in the methods for the work to be reproduced?

Other comments
The title indicates two studies: 1) genome assembly and omics study in paspalum and 2) results from maize which don't link back well to the initial questions posed regarding translation of stress resilience and fertiliser use efficiency from paspalum to crops. There is no evidence for a mechanistic link between any data in paspalum and results of validamycin treatment of maize apart from accumulation of trehalose in both.
Line 457, "prolonged trehalose accumulation eventually leads to a reduced T6P abundance and derepressed SnRK1 activity (refs 66, 68). There is nothing in the literature that shows that prolonged trehalose accumulation leads to less T6P. Hence the statement is wrong and the discussion that follows it is based on a false premise. In fact the opposite is the case see Delatte et al. 2011 10.1104/p.111.180422 where T6P accumulates with prolonged trehalose treatment. SnRK1 activity would be inhibited by elevated T6P not activated as claimed here. The cited refs 66, 68 in line 457 to back up the claim line 457 do not say anything about T6P levels after prolonged trehalose feeding. Ref 68 states that elevated T6P would block activation of autophagy through SnRK1.

Response to reviewers:

Summary of the first three rounds of peer review:

In the first round of peer review, four reviewers assessed our manuscript. They requested a wide range of additional experiments but were overall positive about the impact and broad interest of our manuscript. After reviewing the comments, the managing editor felt that the concerns were all potentially addressable and that the paper was likely to be of interest to Nature Communications if they were addressed. I and members of my lab spent six months conducting the requested experiments including the generation and characterization of new homozygous mutants, generation and analysis of new metabolomic datasets, additional molecular and greenhouse experiments and so on. With a single exception detailed below, we were able to complete all requested new experiments, substantially strengthening the paper. None of the new data generated or new analyses changed the key findings or impact of our manuscript.

In the second round of peer review, Reviewer #1 recommended acceptance, Reviewer #3 was satisfied with our revisions and alternative experiments given the one experiment he or she had requested which we were unable to include (quantifying autophagosomes in a specific maize YFP-ATG8 transgenic line for which we were unable to source seed). Reviewer #4 also stated all of his or her concerns had been addressed.

In sharp contrast to the other three reviewers, reviewer #2 requested new experiments (data on biomass accumulation at multiple time points as well as additional genomic and bioinformatic analyses) in the second round not connected to points he or she had previously raised. Reviewer #2 included the statement "The data are interesting but need much more follow up with evidence before they can be published." We conducted all follow up experiments requested by reviewer #2 in the second round of review. Again, none of the key findings or impact of our manuscript changed.

In the third round of peer review, all three of our prior advocates were gone and only reviewer #2 remained. This time reviewer number #2 did not raise ANY technical concerns. With the exception of pointing out a single formatting error, their entire 3rd review consists of writing over and over and over that he or she didn't think our study was impactful enough for Nature Communications including statements like: **"How would you convince biotech companies to fund this in crops?"**

This last review appears unsound, unprofessional, and constitutes a dramatic moving of the goal posts. The ultimate decision of whether or not a paper is impactful rests with the editorial office, with input from peer reviewers, and NOT with the scientific peer reviewers who assess technical merit and soundness. The handling editor found the conclusions of our paper potential broad interest suitable for Nature Communications after both the first and second rounds of review and our conclusions and findings have not changed in three rounds of review, during which we addressed all technical concerns raised by reviewers.

I include below a point by point response to the statements made by reviewer #2. You will note that only in a single case does the reviewer point is a single thing wrong with our paper (formatting of some citations to papers vs a supplemental figure). Everything else relates to impact, which, given that our key findings and results have not changed since our initial manuscript was submitted to Nature Communications last fall and was found to be of broad interest then, is not material in the third round of peer review.

Reviewer #2 (Remarks to the Author):

• What are the noteworthy results?

1) -Genome assembly of a halophytic grass species, paspalum (new). Genome assembly is of course a good achievement, but for Nature Communications I think that something extra needs to be found that makes the study noteworthy.

R1) It is our view that this decision about noteworthiness for publication in Nature Communication rather than scientific soundness ultimately rests with the editorial office. Three out of four peer reviewers and the handling editor after both the first and second rounds of peer review all found our study noteworthy and none of the key findings or results have changed from the initial submission.

2) I find the follow up to the genome assembly does not provide information that goes beyond speculation of possibilities. The final data in maize on an autophagy mechanism are interesting but do not link back at all well with the genome assembly information in paspalum. It is not clear at all whether the results in maize are applicable to crop improvement. Maize and other cereals have already been targeted successfully through interventions in the trehalose pathway. It is difficult to gain the take home message.

R2) The reviewer has brought up this issue of "linkage" between the different experiments conducted in this study repeatedly. The fact of the matter is that we did experiment X got a result and it lead us to decide to do experiment Y, which produced another result. The reviewer might not have made the same decisions about which lines of research to pursue, but the fact that they did not undermine the soundness of those results. In fact the reviewer does not question the soundness of our results, only whether our results are A) applicable to crop improvement (beyond the scope of this study) and B) novel given the reviewer feels our results have already been applied to crop improvement (which, if true, would seem to contradict his or her first concern).

3) For Nature Communications I would expect a major noteworthy finding. There is lots of suggestive and speculative data requiring further evidence and follow up.

- Genes involved in telomere maintenance and DNA repair have copy number expansion in paspalum as do gene families involved in N and P deficiency (interesting but not especially noteworthy)

-Accumulation of trehalose in paspalum under N deficiency (new information for paspalum but found in other spp. too e.g. tobacco Lin et al. 2017 doi: 10.1186/s12870-017-1207-z; T6P accumulation under low N in Arabidopsis Nunes et al. 2013 10.1104/pp.113.220657). Trehalase expression is the only gene expression change in the trehalose pathway that can really explain trehalose accumulation in paspalum. More rapid rate of protein sequence evolution for trehalose pathway in paspalum is new, but trehalose pathway may be an adaption to the environment in which paspalum found e.g. salinity. No causal link with nutrient deficiency shown. Rapid evolution may or may not relate to accumulation of trehalose under N deficiency. Transcription of genes associated with N and P deficiency are more likely explanations for traits related to N and P deficiency and potential enhanced nutrient use efficiency.

Minor: The reviewer argues that "trehalose pathway may be an adaption to the environment in which paspalum found e.g. salinity" and "Rapid evolution may or may not relate to accumulation of trehalose under N deficiency" in a way that might suggest to someone who hasn't read out paper that our manuscript is trying to assert otherwise. However both of these points come from text of our manuscript itself.

-Validamycin treatment of maize gives rise to elevated trehalose and greater growth under low N through autophagy but also altered N metabolism (as previously shown in tobacco Li et al. 2019 10.1111/pbi.129910 but this does not phenocopy paspalum as claimed. Yields per unit of fertiliser have already been increased through targeting trehalose in maize (Nuccio et al. 2015 10.1038/nbt.3277) and abiotic stress resilience generally in other crops, see below.

R3) Note the reviewer raises no concerns with the validity of the results presented in our manuscript, just his or her personal assessment of noteworthiness. His or her assessment is not in agreement with the views the three reviewers or the handling editor about the significance of our finds through two rounds of peer review and nothing has changed about our findings since that original submission, the newest version of the manuscript simply includes even more and stronger evidence to support those findings.

• Will the work be of significance to the field and related fields? How does it compare to the established literature? If the work is not original, please provide relevant references.

4) -No substantial originality in the work apart from the genome assembly of paspalum. Autophagy findings in maize interesting but may not be related to data in paspalum. Links of trehalose pathway to paspalum phenotype not substantiated.

R4) We disagree with reviewer #2 about the relatedness between different portions of our study. But even if the reviewer were correct, "relatedness" is not a criteria relevant to either scientific soundness (assessed by the reviewers) and broad interest (assessed by the editorial board in the first round of peer review with input from the reviewers). In no version of the manuscript did we claim to demonstrate the trehalose-> autophagy link in paspalum (a wild species), only to conclusively demonstrate it in maize (a crop and genetic model). The lack of evidence to support a claim that was never made is not relevant to assessing the impact of our work in the third round of peer review.

5) -Maize and sorghum already improved through trehalose pathway (Nuccio et al. 2015 10.1038/nbt.3277; Li et al. 2019 10.1111/pbi.12991). Hence novelty for crops not clear. Many cases of trehalose being modified for crop improvement including more yield per unit of fertiliser and water (Garg et al. 2002 10.1073/pnas.252637799 Trehalose accumulation in rice plants confers high tolerance levels to different abiotic stresses) as just one further example.

R5) We agree. As stated in the manuscript, the autophagy mechanism we observe in maize and linked to increased growth under nitrogen constrained conditions via both classical genetics and chemical genetics is the novelty here, not simply the impact of increased trehalose accumulation on growth under nitrogen constrained conditions.

Minor: Contrary to reviewer #2's claims, Garg et al 2002 does not assess yield per unit fertilizer. This is an easy assertion of fact for any member of the Nature Communications office to independently check to see whether or not they should have confidence in the factual nature of statements made by reviewer #2.

• Does the work support the conclusions and claims, or is additional evidence needed?

6) -Current conclusions are all speculative requiring further substantiation. This is not a definitive study apart from providing genome assembly of paspalum.

R6) This is a vague and general statement which 1) is repetitive with previous statements made by the reviewer and 2) lacks any detail that would enable us to respond in a substantive manner. In the absence of any actionable feedback from the reviewer, all we can do here is to reemphasize that our conclusions were reviewed and approved by three other scientists as part of the peer review process.

7) -Genome assembly of paspalum, then a series of observations with not well demonstrated causal links. Speculation. Over interpretation of data.

R7) Just writing the word "Speculation." as a complete sentence is not an informative or factually based review. Nor is "Over interpretation of data" without any specifics, details, or examples. Here again reviewer #2 omits sufficient detail from his or her review to enable a substantive response and simply repeats his or her opinion. Nature Communications cannot control the quality or professionalism of the comments submitted by the scientists they select for peer review, but I believe it is important to recognize handwaving and non-specific negative comments as a serious hindrance to the scientific process rather than a sound basis for conclusions.

8) -Validamycin treatment of maize phenocopies paspalum only with respect to trehalose accumulation, nothing else. Other maize responses do not phenocopy paspalum. More rapid growth is elicited in maize by validA induced trehalose accumulation (opposite of paspalum). Good evidence for autophagy involved in this response in maize is obtained also nitrate transporters and N metabolism (N metabolism upregulation previously found in tobacco when treated with trehalose (Lin et al. 2017), but none of this links back to paspalum. Autophagy not shown in paspalum. Even if autophagy were shown in paspalum how does this explain lack of phenotypic plasticity in paspalum given the exaggerated phenotypic plasticity in maize with trehalose accumulation?

R8) The reviewer agrees we present good evidence of a novel role for autophagy in increased growth of maize under nitrogen limited conditions and also agrees with us that ValA treatment of maize phenocopies paspalum at a biochemical level. Proving the mechanism out in paspalum (as opposed to maize) as never a finding or conclusion we asserted in any version of this manuscript, yet the findings (both the paspalum genome and the conclusive evidence from both chemical and classical genetics that autophagy was a necessary mechanism for the increased growth under nitrogen deficit conditions of maize induced by increased accumulation of autophagy) were still assessed to be of broad interest by the editor and three out of four reviewers.

• Are there any flaws in the data analysis, interpretation and conclusions? Do these prohibit publication or require revision?
9) The paper over interprets preliminary observations. There are no sound conclusions apart from a likely role for autophagy in maize responses to trehalose.

R9) If the reviewer cited specific interpretations we would be able to address any concerns he or she had. Based on the last round of review, he or she was upset about conclusions which were not present in our paper. We were happy to revise our paper to include explicit statements of NOT making the claims the reviewer felt we were making and in fact did so (see our previous response to reviewers' document).

However, after addressing every specific example raised by the reviewer in round 2 of the review, he or she simply repeats the same criticism but without citing any specific interpretations, making his or her comments impossible to address or refute. This is not how to effectively participate in the peer review process.

10) The Discussion is entirely suggestive and speculative. How would you convince biotech companies to fund this in crops?

R10) I've started companies and I can assure you that I wouldn't convince biotech companies to fund this research. Not that this stage anyway. That's why I work at a public university where, if I am successful, I can secure funding our society has made the decision to invest to continuing to develop and prove out ideas biotech companies are not yet willing to invest in to the point where they can have large scale societal impact. I think the editorial board of Nature Communications has to ask themselves whether or not they want to buy into reviewer #2 world view. I don't think the question of what VC firm would invest in is the right filter to identify the science that is likely to have a large, sustained, and positive scientific impact.

Please also note that again the reviewer does not point to any specific examples of points he or she feels are suggestive or speculative, preventing us from making any attempt to address this point.

11) These data in maize do not link back to paspalum. Hence the work is not well connected and does not form a coherent whole.

R11) This is a repeat of point #4. Unfortunately the reviewer repeats this statement, so it wasn't clear how to compose a point by point response without repeating our response:

Again, in no version of the manuscript did we claim to demonstrate the trehalose-> autophagy link in paspalum (a wild species), only in maize (a crop and genetic model). The lack of evidence to support a claim that was never made is not relevant to assessing the impact of our work in the third round of peer review.

• Is the methodology sound? Does the work meet the expected standards in your field?
• Is there enough detail provided in the methods for the work to be reproduced?

Other comments
12) The title indicates two studies: 1) genome assembly and omics study in paspalum and 2) results from maize which don't link back well to the initial questions posed regarding translation of stress resilience and fertiliser use efficiency from paspalum to crops. There is no evidence for a mechanistic link between any data in paspalum and results of validamycin treatment of maize apart from accumulation of trehalose in both.

R12) this is a repeat point #4 and point #11 Unfortunately the reviewer repeats this statement over and over, so it wasn't clear how to compose a point by point response without repeating our response over and over:

Again, in no version of the manuscript did we claim to demonstrate the trehalose-> autophagy link in paspalum (a wild species), only in maize (a crop and genetic model). The lack of evidence to support a claim that was never made is not relevant to assessing the impact of our work in the third round of peer review.

13) Line 457, "prolonged trehalose accumulation eventually leads to a reduced T6P abundance and derepressed SnRK1 activity (refs 66, 68). There is nothing in the literature that shows that prolonged trehalose accumulation leads to less T6P. Hence the statement is wrong and the discussion that follows it is based on a false premise. In fact the opposite is the case see Delatte et al. 2011 10.1104/p.111.180422 where T6P accumulates with prolonged trehalose treatment. SnRK1 activity would be inhibited by elevated T6P not activated as claimed here. The cited refs 66, 68 in line 457 to back up the claim line 457 do not say anything about T6P levels after prolonged trehalose feeding. Ref 68 states that elevated T6P would block activation of autophagy through SnRK1.

13) Thank you for catching this. This was an oversight in editing. The assertion in question should reference a model proposed in Figure S13 and the transcriptomic patterns we observe consistent with that proposed, not references 66, and 68. This has been revised in our resubmission.
